# Comparative impacts and cost-effectiveness of tuberculosis systematic screening strategies in prisons in Brazil, Colombia, and Peru: A mathematical modeling study

**DOI:** 10.1371/journal.pmed.1004739

**Published:** 2026-06-04

**Authors:** Yiran E. Liu, José Victor Bortolotto Bampi, Ronan F. Arthur, Argita D. Salindri, Caroline Busatto, Pedro Avedillo Jiménez, Daniele Maria Pelissari, Fernanda Dockhorn Costa Johansen, Robert Arana-Narvaez, Alvaro Fernando Moreno Roca, Wilfredo Santos Solís Tupes, Esther Mori Jiu, Christian Alfredo Moreno Roca, Erika Albertina Abregú Contreras, Valentina Antonieta Alarcón Guizado, Julián Trujillo Trujillo, Belkys Marcelino, Mónica Alonso Gonzalez, Mayra Cecilia Córdova Ayllon, Ted Cohen, Moises A. Huaman, Jeremy D. Goldhaber-Fiebert, Julio Croda, Jason R. Andrews

**Affiliations:** 1 Division of Infectious Diseases and Geographic Medicine, Department of Medicine, Stanford University, Stanford, California, United States of America; 2 SEICHE Center for Health and Justice, Yale University School of Medicine, New Haven, Connecticut, United States of America; 3 Federal University of Mato Grosso do Sul, Campo Grande, Brazil; 4 National Tuberculosis Program, Ministry of Health, Brasília, Brazil; 5 Pan American Health Organization, Communicable Diseases Prevention, Control and Elimination, Washington, District of Columbia, United States of America; 6 Dirección de Prevención y Control de la Tuberculosis, Ministerio de Salud, Lima, Peru; 7 Ministerio de Salud y Protección Social, Subdirección de Enfermedades Transmisibles, Bogotá, Colombia; 8 School of Public Health, Universidad Autónoma de Santo Domingo, Santo Domingo, Dominican Republic; 9 Instituto Penitenciário Nacional, Lima, Peru; 10 Department of Epidemiology of Microbial Diseases, School of Public Health, Yale University, New Haven, Connecticut, United States of America; 11 Division of Infectious Diseases, Department of Internal Medicine, University of Cincinnati College of Medicine, Cincinnati, Ohio, United States of America; 12 Department of Health Policy, Stanford University School of Medicine, Stanford, California, United States of America; 13 Center for Health Policy, Freeman Spogli Institute, Stanford University, Stanford, California, United States of America; 14 Fiocruz Mato Grosso do Sul, Campo Grande, Brazil; Boston University School of Public Health, UNITED STATES OF AMERICA

## Abstract

**Background:**

Incarceration is a leading driver of tuberculosis in Latin America. Systematic screening in prisons may reduce tuberculosis burden, but optimal strategies and cost-effectiveness remain uncertain. We examined the population-wide health impacts and cost-effectiveness of systematic screening in prisons in Brazil, Colombia, and Peru, comparing different timepoints, frequencies, and screening algorithms.

**Methods and findings:**

Using dynamic transmission models calibrated to Brazil, Colombia, and Peru, we simulated annual or biannual (twice-yearly) prison-wide screening, alone or combined with entry and exit screening from 2026 to 2035. We evaluated four algorithms: (1) symptom screening, (2) chest X-ray with computer-aided detection (CXR-CAD), (3) symptoms and CXR-CAD (follow-up testing if either is positive), and (4) GeneXpert Ultra (Xpert) with pooled sputum. Individuals screening positive then received individual Xpert. We projected impacts on within-prison and population-level tuberculosis incidence in 2035, along with discounted costs (2023 US dollars) and disability-adjusted life years (DALYs). Model projections showed that combined entry, exit, and biannual screening with CXR-CAD was highly impactful and cost-effective across countries, reducing tuberculosis incidence by 61%–87% in prisons and 18%–28% population-wide. Compared to only biannual CXR-CAD (the next best strategy), the incremental cost per DALY averted of adding entry and exit screening was $2,984 (Brazil), $2,925 (Colombia), and $645 (Peru). Adding symptom screening to CXR-CAD marginally increased benefit and was only cost-effective in Peru’s higher-incidence prisons. Biannual screening alone remained cost-effective at prison incidence levels well below national averages, as well as at far lower willingness-to-pay thresholds. In settings without CXR-CAD, pooled Xpert was an impactful, cost-effective alternative. Key limitations include the model’s simplified representation of tuberculosis disease states and lack of stratification by age, gender/sex, HIV, or drug resistance.

**Conclusions:**

These modeling results support immediate national-level adoption of prison-wide tuberculosis screening twice-yearly and at entry and exit, using CXR-CAD or pooled Xpert.

## Author summary

**Why was this study done?:**

- In Latin America, rising incarceration has fueled the tuberculosis epidemic, with extremely high infection rates among people deprived of liberty. These effects extend beyond prison walls, driving tuberculosis spread in outside communities.
- Interventions targeted to prisons may have an outsized impact on reducing tuberculosis in the broader population.
- The World Health Organization strongly recommends systematic screening for tuberculosis in prisons, but evidence is needed to inform how often to screen, which methods to use, and whether these approaches are cost-effective across different country contexts.

**What did the researchers do and find?:**

- We developed mathematical models using data from Brazil, Colombia, and Peru to simulate different prison-based tuberculosis screening strategies and project their health impacts and costs.
- We compared prison-wide screening once or twice a year, screening at prison entry or exit, and combinations of these approaches. We also compared different screening methods using symptoms, chest X-ray with computer-aided detection (CXR-CAD), or pooled molecular testing (GeneXpert Ultra).
- The models projected that combining entry, exit, and twice-yearly prison-wide screening with CXR-CAD would be highly impactful and cost-effective in all three countries. This strategy could substantially reduce tuberculosis in prisons and in the general population.
- Twice-yearly prison-wide screening was projected to remain cost-effective even in prisons with much lower tuberculosis rates than national averages, and in settings with lower willingness-to-pay thresholds.
- CXR-CAD was the optimal screening method, but pooled molecular testing was also impactful and cost-effective where CXR-CAD was not available.

**What do these findings mean?:**

- Systematic screening in prisons, twice-yearly and at entry and exit, is projected to be highly impactful and cost-effective across different settings in Latin America.
- These findings support urgent adoption of intensive prison-based tuberculosis screening throughout the region, alongside efforts to build capacity for CXR-CAD, the most cost-effective screening method.
- The main limitations of the study include a simplified representation of how tuberculosis develops in individuals and spreads across populations. Modeling results should be interpreted alongside empirical evidence that incorporates real-world complexities.

## Abstract 3

In a mathematical modeling study, Yiran Liu and colleagues demonstrate that screening for tuberculosis (TB) in prisons in Peru, Colombia and Brazil twice yearly and at entry and exit is predicted to have a substantial impact on TB incidence in both the prison and general populations.

## Introduction

Tuberculosis persists as a leading global health threat, with an estimated 10.8 million people falling ill and 1.25 million people dying from the disease in 2023 [1]. People deprived of liberty (PDL) experience substantially elevated risk of tuberculosis due to prison conditions like overcrowding, poor ventilation, malnutrition, and limited access to medical care [2]. Timely diagnosis and treatment are an essential component of tuberculosis control in prisons, but studies estimate that only about half of PDL with tuberculosis globally are diagnosed [3].

In Latin America, tuberculosis incidence and deaths have increased since 2015, and the worsening tuberculosis epidemic is increasingly driven by prisons [2]. Our recent modeling study estimated that, following decades of rising incarceration rates, 27% of new cases in the region were attributable to incarceration [4]. Interventions to address tuberculosis in prisons may have an outsized impact on the population-wide tuberculosis epidemic in Latin America.

Systematic screening for tuberculosis may improve early detection and reduce transmission in prisons [5]. Since 2021, the World Health Organization (WHO) has strongly recommended systematic screening in prisons, at minimum annually and upon entry and exit [6]. However, the guidelines cite a “very low certainty of evidence” and highlight the need for more research on the effectiveness and cost-effectiveness of different strategies. Recent empirical studies in prisons have demonstrated high yield from strategies using chest X-ray with computer-automated detection (CXR-CAD) or molecular rapid testing (i.e., GeneXpert) with sputum pooling [7–10]. However, their broader impact and cost-effectiveness remain uncertain, especially across settings with varying tuberculosis epidemiology and carceral characteristics [6]. Moreover, WHO has described biannual (twice-yearly) mass screening in prisons as “ideal,” but lacking economic evidence, cited resource constraints as potential barriers to sustained implementation [11]. Existing modeling studies in the region offer limited guidance: of two prior studies, both were conducted in Brazil only and used subnational data, and neither assessed cost-effectiveness nor modeled screening more than once per year [12,13].

In this study, we utilize a compartmental dynamic transmission model to project the population-level impact and cost-effectiveness of tuberculosis systematic screening interventions in prisons in Brazil, Colombia, and Peru. We evaluate strategies for screening across different timepoints (i.e., entry, exit, periodic), frequencies (annual or biannual), and algorithms (symptom screening, CXR-CAD, combined symptoms and CXR-CAD, or GeneXpert Ultra with sputum pooling). All algorithms include follow-up testing with individual GeneXpert Ultra. We also perform sensitivity analyses to identify preferred strategies in settings with lower prison incidence or without access to CXR-CAD technology.

## Methods

### Transmission model and key assumptions

**Fig 1:**
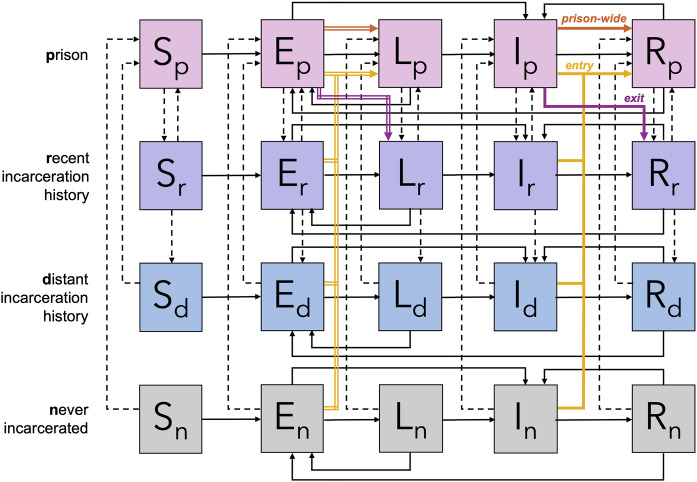
Compartmental transmission model schematic. The model includes five tuberculosis disease states: susceptible **(S)**, early latent infection **(E)**, late latent infection **(L)**, infectious active disease **(I)**, recovered **(R)**. These disease states are replicated across four incarceration-related population strata, denoted with subscripts: prison **(p)**, recent history of incarceration **(r)**, distant history of incarceration **(d)**, never incarcerated **(n)**. Solid black arrows represent tuberculosis natural history transitions (e.g., infection, progression to disease, recovery, relapse). Dashed black arrows represent transitions between population strata (e.g., incarceration, release, re-incarceration). Solid colored arrows indicate transitions resulting from systematic screening interventions, which move individuals from infectious active disease (I) to recovered **(R)**. Hollow (double-lined) colored arrows indicate additional transitions resulting from screening wherein individuals in early latent infection (E) who test false positive and receive treatment for infectious, active disease move to late latent infection **(L)**. Entry screening (including screening upon inter-prison transfer) is shown in gold; exit screening is shown in purple, and prison-wide screening (annual or biannual) is shown in orange. For entry and exit screening, recovery from disease occurs concurrently with incarceration-related transitions.

We extended a published compartmental transmission model simulating tuberculosis and incarceration dynamics (Fig 1) [4]. The model includes a simple representation of tuberculosis natural history for the population 15 years of age and older. Upon infection, **S**usceptible individuals transition to **E**arly latent infection, after which they activate to **I**nfectious disease or transition to **L**ate latent infection, with lower risk of active disease. Individuals in **I** transition to **R**ecovered through self-cure or diagnosis and successful treatment. Individuals in **L** and **R** can be re-infected to **E**, and individuals in **R** can relapse to **I**.

These natural history compartments are reproduced across four population strata, which individuals traverse upon incarceration, release, and re-incarceration: prison (**p)**, recent history of incarceration (**r)**, distant history of incarceration (**d)**, and never incarcerated (**n**). The model is not stratified by age, gender/sex, or HIV. We simplify the documented spectrum of TB disease states [14], assuming a single disease state (**I**) representing infectious, bacteriologically positive tuberculosis, of which 51% (range 30%–70%) is assumed to be symptomatic (Table 1). Multi-drug resistant (MDR) tuberculosis was not modeled explicitly but was accounted for by adjusting treatment outcomes and costs based on relative proportions of drug-sensitive and drug-resistant tuberculosis.

**Table 1:** Accuracy and costs of screening interventions.

	Sensitivity (%)	Specificity (%)	Per-unit cost (2023 USD)			Source
			Brazil	Colombia	Peru	
Symptom screen	51 (30–70)	70 (60–80)	2.27 (2.00–3.00)	1.58 (1.39–2.08)	1.76 (1.50–2.50)	[6,7,22,31,32]
CXR-CAD	85 (75–88.5)	81 (70–93)	10.48 (8.28–13.60)	9.10 (7.40–11.48)	9.34 (7.24–12.19)	[6,7,10,22]
Symptoms and CXR-CAD	89 (84–93)	52 (41–63)	10.48 (8.28–13.60)	9.10 (7.40–11.48)	9.34 (7.24–12.19)	[10,33]
Pooled Xpert	66.1 (55.3–78.2)	98.8 (97–99.5)	19.38 (13.23–31.12)	14.01 (9.46–21.61)	15.62 (10.47–24.49)	[7,34–36]
Follow-up testing	90.9 (86.2–94.7)	98 (96–99.5)	26.43 (19.90–38.38)	21.93 (16.81–30.86)	23.50 (17.57–33.06)	[36]

Values listed are the mode (range) from a triangle distribution or the median (range) when values are composites of individual parameters. All costs are in 2023 US dollars (USD) and include consumable and personnel costs. Listed costs of CXR-CAD alone and pooled GeneXpert Ultra (Xpert) also include costs of administering a symptom interview. Follow-up testing includes clinical evaluation and Xpert Ultra. Sensitivity and specificity estimates were derived from studies that used bacteriologically confirmed tuberculosis as the reference standard, consistent with the model’s representation of tuberculosis disease. For pooled Xpert, sensitivity is derived from the sensitivity of Xpert Ultra in a screening context, multiplied by the relative sensitivity of using pooled samples. For costs of pooled Xpert, cartridge savings vary based on TB prevalence; listed costs are the median (range) of costs over the 10-year intervention horizon for annual screening. Additional intervention and cost parameters, including costs of individual components, are provided in Table B in S1 Appendix.

Calibrated parameters were sourced directly from a prior study [4], wherein the model was separately calibrated for Brazil, Colombia, and Peru to country-specific data from 1990 to 2023 (Table A in S1 Appendix). Data targets included prevalence of incarceration, prison admission rates, recidivism, population-wide tuberculosis notifications and incidence, and within-prison tuberculosis notifications and incidence [4]. Calibration targets for prison incidence were based on a previous study using Bayesian meta-regression modeling to estimate within-prison case-detection ratios (S1 Appendix) [3]. Model fit to data is shown in Figs A and B in S1 Appendix. From 2023 on, we assume stable prison entry and release rates, with incarceration rates eventually leveling off. The elevated risk of tuberculosis in prisons is operationalized through higher effective contact rates, higher disease progression rates, and lower diagnosis rates in prisons than in the community [4]. Individuals with recent incarceration history are assumed to have similar contact rates as never-incarcerated individuals, but higher disease progression rates and lower diagnosis rates [4]. The transition from recent to distant incarceration history occurs on average at seven years following release, after which rates of contact, progression, and diagnosis match those of never-incarcerated individuals [12].

### Interventions and scenarios

In the base-case scenario, we assume only passive diagnosis in prisons. Under passive diagnosis, people with presumptive TB undergo clinical evaluation, with a proportion receiving bacteriologic testing using smear microscopy and/or GeneXpert Ultra. A proportion of confirmed cases additionally receive culture and drug susceptibility testing (DST). Coverage of each testing method is informed by country-specific programmatic data (Table B in S1 Appendix).

We simulated prison-based systematic screening interventions, in addition to passive diagnosis, implemented from the start of 2026 through the end of 2035. We modeled systematic screening at prison entry or exit, prison-wide screening conducted annually or biannually (twice yearly), or a combination of entry, exit, and annual or biannual screening (S1 Appendix). We assume entry screening applies to individuals entering prison from the community as well as individuals transferred between prison facilities. Screening of transferred individuals was modeled by adding a continual rate of screening to the prison stratum; movements between prisons were not explicitly modeled. Exit screening only applies to individuals exiting prisons to the community. Parameters governing screening coverage, duration, linkage to care post-release, treatment completion in prison and post-release, and costs are detailed in Table B in S1 Appendix. Individuals in **E** who test false positive for infectious tuberculosis disease through systematic screening and complete treatment transition to **L** (S1 Appendix). Other false positives do not undergo any model-based transitions.

### Screening algorithms and assumptions

For each intervention, we compared four screening algorithms: (1) symptom-based, (2) CXR-CAD, (3) combined symptoms and CXR-CAD, and (4) GeneXpert Ultra MTB/RIF (Xpert) with pooled sputum. We used empirical accuracy estimates from studies of systematic screening in prisons when available. Symptom screening was based on cough of any duration, the symptom for which the most data from prisons are available. For combined symptoms and CXR-CAD, individuals with either symptoms or an abnormal X-ray (or both) are considered screen-positive. For sputum pooling, samples from eight individuals (the most efficient pool size based on prior work) are combined and tested using a single Xpert cartridge; we assume that pooling reduces Xpert sensitivity but does not affect specificity [9]. Smaller pools may yield increased sensitivity at greater cost. If the pooled test is positive, each individual sample in the pool is tested separately to identify screen-positive individuals. Individuals who screen positive receive follow-up testing with individual GeneXpert Ultra on a new sputum sample and clinical evaluation. Accuracy and costs of screening methods and follow-up testing are shown in Table 1.

All individuals diagnosed through screening (including false positives) receive culture, DST, and treatment. Individuals diagnosed through exit screening initiate treatment only if they are linked to care. In any intervention, individuals who initiate but do not complete treatment are assumed to incur half the treatment costs.

### Costs and outcomes

We projected the percent reduction in prison and population tuberculosis incidence in 2035, the last year of the intervention period, compared to the base-case scenario. Across all population strata, we projected disability-adjusted life years (DALYs) due to tuberculosis and post-tuberculosis sequelae [15] and estimated costs from the health system perspective, including costs of screening, follow-up testing, diagnosis, and treatment for cases detected through passive diagnosis and systematic screening. We used a 10-year analytic horizon, accounting for lifetime DALY streams. Costs are reported in 2023 US dollars (USD), adjusted for inflation using the World Bank GDP deflator [16]. Costs and DALYS were standardized per 100,000 population and discounted at 3% annually [17].

DALY-related parameters are shown in Table 2. To project lifetime years of life lost (YLLs) for people who die from tuberculosis, we calculated the average age of people developing tuberculosis among the population 15 and older [1] (37 in Brazil, 40 in Colombia, and 37 in Peru) and retrieved remaining life expectancy at that age (Table 2). We assumed similar life expectancy across population strata. To project years lived with disability (YLDs) due to post-tuberculosis sequelae, we applied a post-tuberculosis disability weight over the remaining life expectancy of individuals who recover from tuberculosis (Table 2). At the end of the analytic horizon, individuals with tuberculosis were assumed to either recover or die immediately from tuberculosis with probabilities based on competing risks. We did not include elevated all-cause mortality for tuberculosis survivors given uncertainty around the true causal risk ratio. Treatment was assumed to have no disability weight for true and false positives.

**Table 2:** Parameters for DALY estimation.

Parameter	Country	Distribution	Source
Disability weight for infectious active TB disease	All	Triangle(0.33, 0.25, 0.4)	[37]
Post-TB disability weight	All	Triangle (0.038, 0.02, 0.05)	[15]
TB mortality rate	All	Uniform(0.061, 0.21)	[38]
Remaining life expectancy at average age of developing TB among population aged 15 and older (years)	Brazil	Triangle (44, 39, 48)	[39]
	Colombia	Triangle (42, 37, 47)	[39]
	Peru	Triangle (47, 42, 52)	[39]

The TB mortality rate is an annualized rate assuming a prevalence of smear-positive TB ranging between 10% and 50%. Triangle distributions are specified as Triangle(mode, minimum, maximum). TB, tuberculosis.

Cost parameters were sourced from costing studies conducted in the selected countries (S1 Appendix). Costs in the base-case scenario include costs of passive diagnosis and treatment. Intervention costs include screening and treatment. Costs for each screening algorithm include costs of equipment, consumables, and labor for screening and follow-up testing (including culture and DST). Costs of treatment were assumed to be the same for patients diagnosed through passive diagnosis and systematic screening and include costs of drugs, hospitalization, directly observed treatment (DOT), and follow-up evaluation and testing for the average patient. Intervention costs included treatment costs for false positives detected through systematic screening. False positives under passive diagnosis were not accounted for. We report costs relative to the base-case scenario as the net of intervention costs and cost savings from averted cases.

For strategies on the cost-effectiveness efficiency frontier, we calculated incremental cost-effectiveness ratios (ICERs), defined as the additional cost for each additional DALY averted compared to the next-best non-dominated strategy [17]. Strategies were considered cost-effective if their ICERs were within country-specific cost-effectiveness thresholds: $8,602 (Brazil), $5,618 (Colombia), and $5,731 (Peru) [18–20] (S1 Appendix). For each country, the optimal strategy was defined as the strategy that provided the greatest health benefit (DALYs averted) while remaining within the cost-effectiveness threshold.

This study is reported as per the Strengthening the Consolidated Health Economic Evaluation Reporting Standards 2022 (CHEERS 2022) Statement (S1 CHEERS Checklist). A health economic analysis plan was not developed prior to the study being conducted.

### Uncertainty and sensitivity analyses

We quantified uncertainty in data targets, epidemiologic parameters, intervention parameters, cost inputs, and model assumptions, but not in model structure. We sampled 1,000 sets of intervention parameters and cost inputs from uncertainty distributions using Latin Hypercube sampling. These sets were randomly paired with 1,000 sets of fitted model parameters, calibrated to data targets also sampled from uncertainty distributions [4]. We repeated the analysis for each set of parameters and inputs, generating a distribution for each outcome, from which we report summary values (mean or median) and 95% uncertainty intervals. For the cost-effectiveness analysis, we assessed joint uncertainty in additional costs (relative to the base case scenario) and DALYs averted for each strategy. We also conducted a probabilistic sensitivity analysis and generated cost-effectiveness acceptability curves (CEACs), depicting the probability each strategy yields the greatest net monetary benefit at varying willingness-to-pay thresholds. We additionally plot the cost-effectiveness acceptability frontier, indicating the optimal strategy across willingness-to-pay thresholds, which may differ from the strategy with the highest probability of being cost-effective [21].

We performed several additional sensitivity analyses. First, we examined how variation in within-prison tuberculosis incidence could affect the optimal screening strategy. We generated scenarios with lower prison incidence by varying within-prison transmission rates, representing variation in prison conditions (i.e., crowding, ventilation). We then conducted multinomial logistic regression and estimated the probability that each strategy would be optimal under varying prison incidence levels. Second, we re-examined the efficiency frontier and preferred strategy in a scenario without access to CXR-CAD technology, which remains the current reality for most prisons in low- and middle-income countries. Third, repeated intensive screening may lead to earlier detection and treatment, such that the clinical and bacteriological profile of TB in the screened population may shift over time toward less severe disease (i.e., higher proportions of asymptomatic and/or bacteriologically negative TB). To account for this, we gradually decreased test sensitivity for both screening and follow-up testing over the first half of the intervention period, assuming that after five years of intensive screening, test sensitivity converges to values consistent with asymptomatic TB. Details on sensitivity analyses are provided in the S1 Appendix.

## Results

### Carceral characteristics and status quo tuberculosis incidence

The median projected incarceration rate in 2026 among the population aged 15 and older was 286 per 100,000 in Colombia, 376 per 100,000 in Peru, and 476 per 100,000 in Brazil (Fig C in S1 Appendix). Carceral dynamics also varied across countries. The average duration of incarceration ranged from 1.3 years in Brazil to 4.9 years in Peru, and the proportion entering prison with prior incarceration history ranged from 18% in Colombia to 60% in Brazil.

In the base-case scenario without systematic screening in prisons, the model projected that by 2035, the incidence of tuberculosis in prisons would be approximately 2,000 (95% uncertainty interval UI [1,600,2,700]) per 100,000 person-years in Brazil, 1,500 (95% UI [1,000,2,500]) per 100,000 in Colombia, and 6,100 (95% UI [4,100,9,600]) per 100,000 in Peru (Fig C in S1 Appendix). At the population level, projected tuberculosis incidence in 2035 without screening in prisons was 33 (95% UI [28,41]) per 100,000 in Colombia, 42 (95% UI [38,47]) per 100,000 in Brazil, and 105 (95% UI [83,130]) per 100,000 in Peru.

### Intervention impacts on prison and population-wide tuberculosis incidence

**Fig 2:**
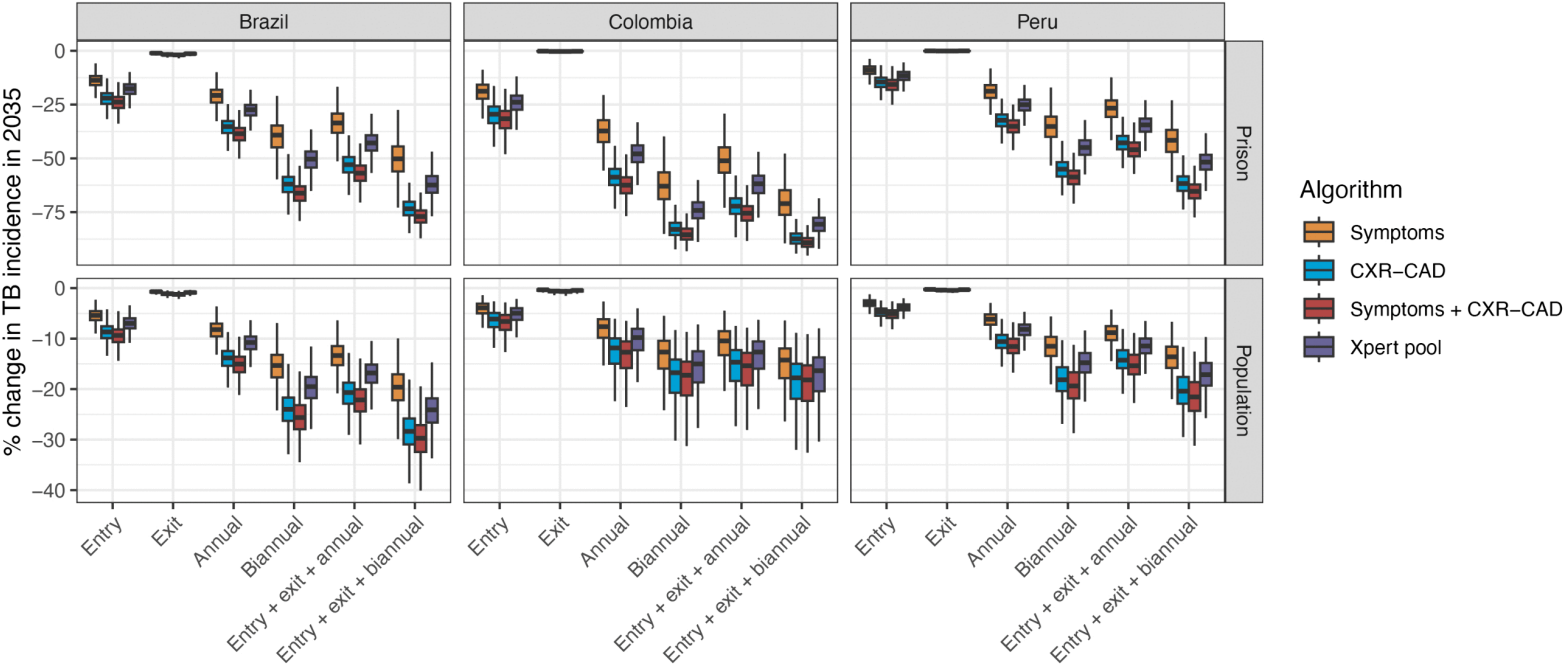
Impacts of screening interventions on within-prison and population-wide tuberculosis incidence in 2035. Boxplots show the median (center line), interquartile range (box limits), and 1.5*interquartile range (whiskers) across 1,000 model simulations and parameter sets per country. Outliers are not shown. Note: y-axis scales differ between the upper and lower panel. CXR-CAD, chest X-ray with computer-aided detection.

Annual screening was projected to significantly reduce in-prison and population-wide tuberculosis incidence across countries. Compared to the base-case scenario, annual screening with CXR-CAD could reduce prison incidence in 2035 by 35.3% (95% UI [27.9,43.8]) in Brazil, 58.7% (95% UI [48.4,69.4]) in Colombia, and 32.3% (95% UI [24.2,39.7]) in Peru (Fig 2 and Table C in S1 Appendix). These correspond to population-level incidence reductions of 13.8% (95% UI [10.2,18.7]) in Brazil, 11.9% (95% UI [7.2,20.7]) in Colombia, and 10.6% (95% UI [7.4,14.2]) in Peru.

Biannual (twice-yearly) screening could yield even greater impacts than annual screening. Biannual screening with CXR-CAD was projected to reduce prison incidence in 2035 by 61.9% (95% UI [52.0,71.9]) in Brazil, 83.1% (95% UI [74.7,89.6]) in Colombia, and 55.0% (95% UI [45.7,63.9]) in Peru (Fig 2 and Table C in S1 Appendix). At the population level, biannual screening with CXR-CAD could reduce incidence by 24.0% (95% UI [18.1,31.2]) in Brazil, 16.8% (95% UI [10.4,28.4]) in Colombia, and 18.1% (95% UI [12.6,24.4]) in Peru. Compared to annual screening with CXR-CAD, biannual screening with CXR-CAD averted, on average, 58% more DALYs in Brazil, 35% more DALYs in Colombia, and 46% more DALYs in Peru (Table 3). Biannual screening even averted more DALYs than the combination of entry, exit, and annual screening, highlighting the favorable effectiveness of prison-wide screening (Table D in S1 Appendix).

**Table 3:** Costs, effects, and cost-effectiveness of strategies on the efficiency frontier.

Strategy (intervention, algorithm)	Cost (USD)	Effect (DALYs averted)	Increm. cost	Increm. effect	ICER
Brazil					
Base case	304,676	0	NA	NA	NA
Annual, CXR-CAD	342,428	132	37,752	132	286
Biannual, CXR-CAD	385,659	208	43,231	76	565
Entry + exit + biannual, CXR-CAD	500,133	247	114,474	38	2,984
Entry + exit + biannual, symptoms & CXR-CAD	660,055	257	159,922	10	15,416
Colombia					
Base case	233,696	0	NA	NA	NA
Annual, CXR-CAD	256,909	120	23,213	120	193
Biannual, CXR-CAD	284,616	163	27,707	42	654
Entry + exit + biannual, CXR-CAD	320,799	175	36,183	12	2,925
Entry + exit + biannual, symptoms & CXR-CAD	391,939	180	71,140	4	16,207
Peru					
Base case	1,017,841	0	NA	NA	NA
Annual, CXR-CAD	1,045,096	426	27,254	426	64
Biannual, CXR-CAD	1,060,596	621	15,501	195	79
Entry + exit + biannual, CXR-CAD	1,098,123	679	37,526	58	645
Entry + exit + biannual, symptoms & CXR-CAD	1,203,201	704	105,078	25	4,158

Mean estimates are shown, standardized per 100,000 population. All estimates are population-wide (i.e., they include costs and effects accrued by the entire population, not just those in prison). Costs are in 2023 US dollars (USD). Only strategies on the cost-effectiveness efficiency frontier are shown; all strategies are shown in Table D in S1 Appendix. DALYs, disability-adjusted life years; increm, incremental; ICER, incremental cost-effectiveness ratio; CXR-CAD, chest X-ray with computer-aided detection; NA, not applicable.

Combining entry, exit, and biannual screening was the most impactful strategy. Conducted with CXR-CAD, this combination was projected to reduce prison incidence by 73.5% (95% UI [63.6,81.9]) in Brazil, 86.5% (95% UI [82.7,91.7]) in Colombia, and 61.4% (95% UI [52.9,69.6]) in Peru (Fig 2 and Table C in S1 Appendix). This amounted to population tuberculosis incidence reductions of 28.4% (95% UI [21.6,36.5]) in Brazil, 17.8% (95% UI [11.0,29.7]) in Colombia, and 20.4% (95% UI [14.1,27.2]) in Peru.

After interventions ended, impacts were not sustained, with incidence returning to baseline levels within 5–10 years (Fig D in S1 Appendix).

### Comparative impacts of different screening algorithms

Algorithms involving CXR-CAD were most impactful in reducing prison and population incidence. When used for biannual screening, CXR-CAD was projected to yield 33%–58% greater reductions in prison incidence than symptom screening across countries (Table C in S1 Appendix). Projected impacts from screening with both symptoms and CXR-CAD were slightly greater than, but largely comparable to, those from screening with CXR-CAD alone (Fig 2 and Table C in S1 Appendix).

Following algorithms involving CXR-CAD, the next most impactful algorithm was GeneXpert with sputum pooling, which yielded 20%–28% greater projected reductions in prison incidence than symptom screening when used biannually (Table C in S1 Appendix).

Projected impacts of symptom screening were less than those of more sensitive algorithms but still substantial, especially with periodic prison-wide screening. In fact, biannual symptom screening had projected impacts comparable to those of annual screening with CXR-CAD (Table 3 and Table C in S1 Appendix).

### Costs and cost-effectiveness of screening strategies

**Fig 3:**
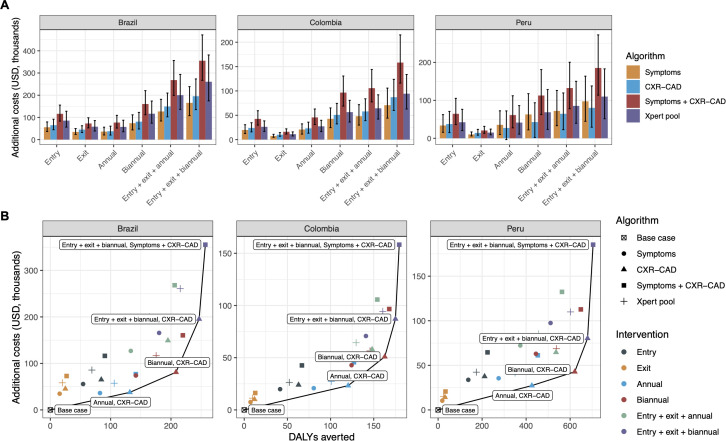
Costs and cost-effectiveness of screening interventions. **(A)** Mean additional cost of screening interventions relative to the base-case scenario. The error bars indicate the 95% uncertainty interval. **(B)** Cost-effectiveness planes for each country. The black line indicates the efficiency frontier. Costs (2023 USD) are additional relative to the base case scenario. Note: axis scales differ across panels to accommodate different ranges across countries. CXR-CAD, chest X-ray with computer-aided detection; DALYs, disability-adjusted life years.

Table D in S1 Appendix reports DALYs averted, total costs, and additional costs relative to the base-case scenario for all interventions and screening algorithms evaluated. Of all screening strategies assessed, four were on the efficiency frontier, exhibiting the greatest health benefit (i.e., averted the most DALYs) at a given cost (Fig 3 and Table 3). All other strategies were strictly dominated (i.e., offer less health benefit at equivalent or higher cost) or weakly (extended) dominated (i.e., an alternative exists that offers better value for money) (Table D in S1 Appendix).

The four non-dominated strategies on the efficiency frontier, ordered from least to most costly and effective, were:

1. Annual screening with CXR-CAD;
2. Biannual screening with CXR-CAD;
3. Entry, exit, and biannual screening with CXR-CAD; and
4. Entry, exit, and biannual screening with symptoms and CXR-CAD.

In Brazil and Colombia, strategy 3 (entry, exit, and biannual screening with CXR-CAD) was the optimal strategy, providing the greatest health benefit within each country’s cost-effectiveness threshold. Specifically, upgrading from biannual screening to the combination of entry, exit, and biannual screening with CXR-CAD had an ICER of $2,984 in Brazil and $2,925 in Colombia (Table 3). Moving from strategy 3 to 4 (i.e., screening with both symptoms and CXR-CAD rather than CXR-CAD alone) averted few additional DALYs at much higher cost, with ICERs of $15,416 in Brazil and $16,207 in Colombia, exceeding both countries’ cost-effectiveness thresholds. Examining the breakdown of intervention costs revealed that strategy’s 4 additional costs were driven by substantially higher costs of follow-up testing and unnecessary treatment, due to the algorithm’s lower specificity (Fig E in S1 Appendix).

In Peru, the ICER for moving from strategy 3 to 4 was $4,158, within the cost-effectiveness threshold, suggesting that, in the highest burden settings, the additional benefits justify the additional costs (Table 3).

Of note, the combination of entry, exit, and annual screening—the current WHO recommendation—was dominated by biannual screening in all three countries (Fig 3 and Table D in S1 Appendix). In other words, countries that have implemented annual screening would get more health benefit while spending less by increasing screening frequency to twice yearly, rather than by adding entry and exit screening.

False positives accounted for a substantial proportion of individuals diagnosed by all algorithms except Xpert on pooled sputum; this proportion generally increased with screening intensity (Fig F in S1 Appendix).

### Sensitivity analysis: Decision uncertainty

Probabilistic sensitivity analyses showed considerable joint uncertainty and overlap in costs and DALYs averted across strategies (Fig G in S1 Appendix). Nonetheless, cost-effectiveness acceptability curves indicated strong preferences for the combination of entry, exit, and biannual screening at country-specific willingness-to-pay thresholds (Fig H in S1 Appendix). Screening with CXR-CAD alone was most cost-effective in 95% of iterations in Brazil and 81% in Colombia, while in Peru the preferred strategy alternated between using CXR-CAD alone (36%) and using both symptoms and CXR-CAD (64%). Biannual screening with CXR-CAD became the preferred strategy at lower willingness-to-pay thresholds.

### Sensitivity analysis: Varying prison incidence

**Fig 4:**
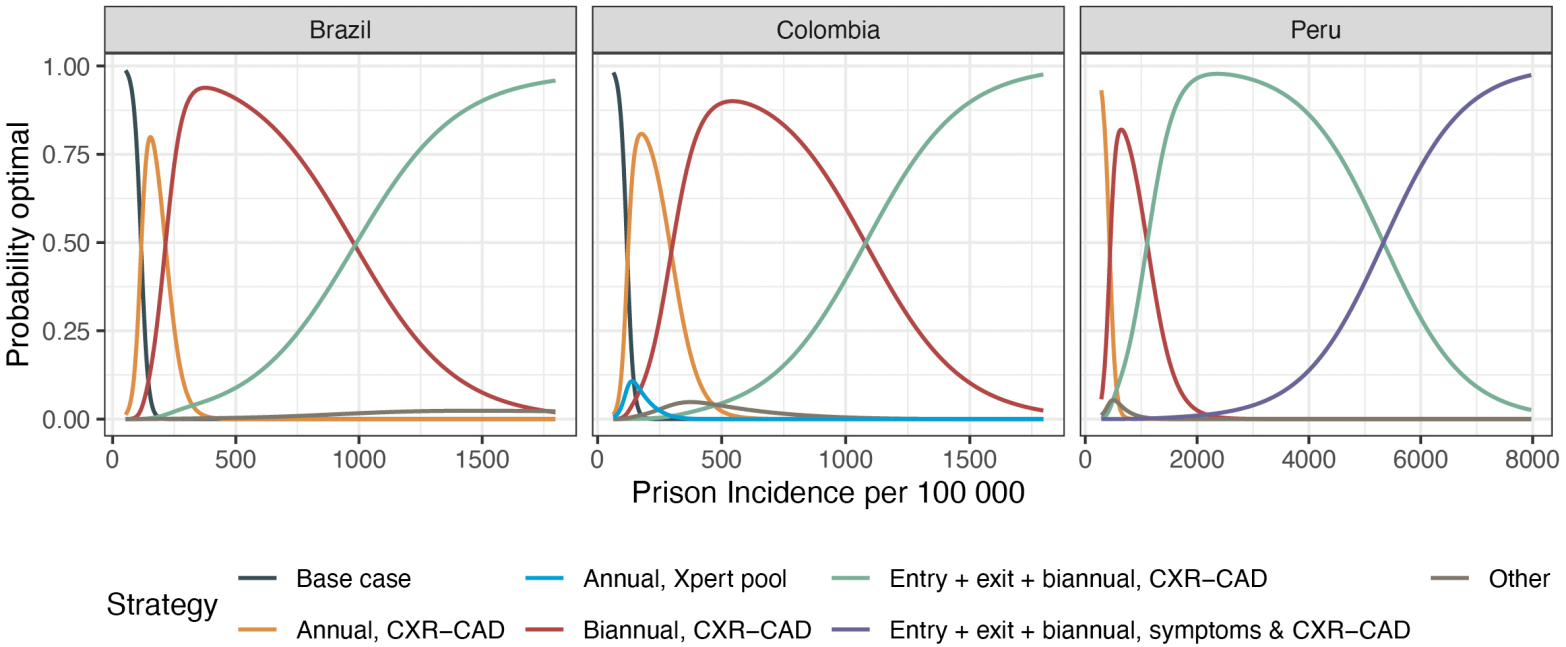
Optimal strategies under varying prison incidence scenarios. Prison incidence was varied by varying the rate of within-prison transmission. The probability that a given strategy was optimal across a range of prison incidence was modeled using multinomial logistic regression. Only strategies with modeled probabilities greater than or equal to 5% in at least one incidence quartile are shown; all other strategies are lumped into “other”. Prison incidence under approximately 300 per 100,000 was not modeled in Peru due to parameter constraints (see S1 Appendix, sensitivity analyses).

We examined how the optimal strategy would vary by prison incidence, by simulating scenarios with differing levels of within-prison transmission. Entry, exit, and biannual screening with CXR-CAD remained the optimal strategy at prison incidence levels above approximately 1,000 per 100,000 person-years (Fig 4 and Table E in S1 Appendix). At extraordinarily high prison incidence levels (>5,300 per 100,000 person-years), combining symptoms and CXR-CAD for entry, exit, and biannual screening became the preferred strategy.

At lower prison incidence levels, less intensive strategies were optimal. Annual screening with CXR-CAD was cost-effective at prison incidence levels greater than approximately 120 per 100,000 person-years (Fig 4 and Table E in S1 Appendix). Biannual screening with CXR-CAD was cost-effective at prison incidence levels above approximately 210 per 100,000 in Brazil, 300 per 100,000 in Colombia, and 440 per 100,000 in Peru.

### Sensitivity analysis: No CXR-CAD

If CXR-CAD were unavailable, the best alternative strategy would be entry, exit, and biannual screening using Xpert on pooled sputum (Table F in S1 Appendix). Compared to the next best strategy (biannual screening with pooled Xpert), the combination of entry, exit, and biannual screening with pooled Xpert had an ICER of $3,607 in Brazil, $2,551 in Colombia, and $642 in Peru. Other strategies on the efficiency frontier included annual and biannual screening with symptoms or pooled Xpert, depending on the country (Fig I in S1 Appendix).

### Sensitivity analysis: Declining test sensitivity

Assuming declining test positivity among individuals with tuberculosis due to a shift toward less severe disease, the combination of entry, exit, and biannual screening with CXR-CAD would avert 89% of DALYs in Brazil, 94% of DALYs in Colombia, and 90% of DALYs in Peru relative to the main analysis (Table G in S1 Appendix). Additional costs were 4% higher in Brazil, 2% higher in Colombia, and 22% higher in Peru, driven by smaller offsets from averted passive diagnosis costs.

## Discussion

Evidence is lacking to guide optimal strategies for systematic screening of tuberculosis in prisons. In this study, we used a dynamic transmission model to project the population-wide impacts and cost-effectiveness of different prison-based screening approaches in Brazil, Colombia, and Peru. We find that combined entry, exit, and biannual screening with CXR-CAD is the most impactful and cost-effective strategy in Brazil and Colombia, while a more sensitive algorithm (combining symptoms and CXR-CAD) is justified in Peru’s higher-burden prisons. These results support prioritizing intensive, periodic prison-based screening up to twice yearly, particularly in settings where prisons drive tuberculosis transmission.

The WHO recommends prison-wide screening at least annually and has stated that biannual mass screening, while “ideal,” may not be sustainable due to cost and logistical barriers [6,11]. As the first modeling study to evaluate screening more than once a year, we found that biannual prison-wide screening, alone or in combination with entry and exit screening, was highly cost-effective across heterogeneous countries and led to substantial reductions in tuberculosis incidence in prisons and in the general population. Importantly, these intensive strategies remained cost-effective at prison incidence levels far below national averages, with biannual CXR-CAD alone remaining cost-effective at levels as low as 200–400 per 100,000. These findings strongly support implementation and regional scale-up of twice-yearly screening in prisons, with investments justified by their projected population-wide benefits.

Regarding screening algorithms, strategies involving CXR-CAD were consistently the most impactful and cost-effective, aligning with empirical studies demonstrating its feasibility and cost-efficiency in prison settings [7,8,22–24]. Prior modeling work in other regions has also supported the value of CXR-based screening in prisons [13,25]. In Brazil and Colombia, entry, exit, and biannual screening with CXR-CAD alone was optimal. In Peru, where the model projected prison incidence to exceed 5,000 cases per 100,000 person-years without screening, combining symptoms and CXR-CAD was cost-effective. In the model, high prison incidence reflects a combination of high within-prison transmission, rapid disease progression, and limited passive diagnosis. Our results suggest that under extreme combinations of these conditions, a slight gain in screening sensitivity justifies the increased costs. Of note, in 2023 the Peru National Tuberculosis Program (DPCTB) began implementing entry and annual screening using symptoms and CXR-CAD, reaching nearly 40,000 PDL across 18 prisons [8]. Overall, our results strongly support using CXR-CAD for systematic screening in prisons. While implementation requires upfront investments in equipment, software, and training, we accounted for these costs in our analysis, and CXR-CAD remained the cost-effective algorithm. Moreover, advances in ultra-portable X-ray technology may further improve scalability and sustainability of intensive periodic screening by enabling coverage of multiple prisons with a single device [10].

In the absence of CXR-CAD, pooled Xpert was a favorable alternative, averting 87%–92% as many DALYs as CXR-CAD and remaining highly cost-effective across countries. While most symptom-based strategies were dominated, intensive (i.e., biannual) symptom screening nonetheless averted substantial DALYs—more than annual screening with CXR-CAD. These findings highlight the importance of immediately implementing prison-wide screening with available tools while developing capacity for more sensitive algorithms.

Entry or exit screening alone had modest impact compared to periodic prison-wide screening, and biannual screening dominated WHO-recommended combination of entry, exit, and annual screening. These results can be attributed to the region’s underlying epidemiology, reflected in the calibrated model, where tuberculosis prevalence and transmission intensity are substantially higher in prisons than in communities [12,26]. As a result, prison-wide screening generates greater yield per person screened than adding entry screening, and results in greater downstream transmission effects than exit screening. In settings with less disparity between prison and community burden and transmission rates, the impacts and cost-effectiveness of entry and exit screening may be relatively greater than observed here.

Notably, we project that if any screening intervention were to end without additional efforts to reduce within-prison transmission, tuberculosis rates would rebound rapidly to pre-intervention levels. To enable sustained progress toward tuberculosis elimination, intensive screening should be paired with complementary interventions like tuberculosis preventive therapy, as well as structural efforts to improve prison conditions and reduce incarceration rates [4,27,28].

There are several limitations to note. Our model did not include age, gender, or HIV, although HIV prevalence is relatively low in these countries [29]. We did not model the spectrum of tuberculosis disease states, which may be particularly relevant under repeated screening if the distribution of TB disease shifts towards less detectable forms over time. In a sensitivity analysis assuming declining test positivity among those with tuberculosis, intensive screening averted slightly fewer DALYs at similar or moderately higher cost. This suggests that our simplified representation of TB disease states may modestly underestimate ICERs but likely does not change conclusions regarding the cost-effectiveness of intensive screening with CXR-CAD. In extremely high incidence prisons where our main results suggest that screening with both symptoms and CXR-CAD would be justified, a shift toward predominantly asymptomatic disease would weaken this justification. Relatedly, we did not model bacteriologically negative tuberculosis, which accounts for approximately 10% of diagnoses in this setting [7]; future work might explore how inclusion of empirical treatment in prison screening affects impacts and cost-effectiveness. Our simplified approach to representing MDR-TB does not capture complexities of longer treatment regimens or diagnostic delays, which may influence preferred screening strategies. A study modeling tuberculosis screening in prisons in the former Soviet Union, where MDR-TB rates are high, found that annual screening with Xpert was more effective in reducing MDR-TB prevalence compared to annual radiographic screening [25]. Pooled Xpert may be a useful tool in high MDR-TB prisons but should be tested empirically. We assumed equivalent effective contact rates across community-based strata; if recently incarcerated individuals have higher contact rates, the downstream transmission effects of prison-based screening might be underestimated. We assumed that all false positives receive a full confirmatory work-up and treatment; in practice, some may be ruled out earlier (e.g., after a negative culture), and our approach may therefore overestimate costs. Furthermore, we did not explicitly model prison transfers, which could reduce effectiveness if screening is not coordinated across facilities. This may partly explain the discrepancy between our results and a recent empirical study in Brazil, which found no reduction in tuberculosis prevalence after three rounds of prison mass screening between 2017 and 2021 [30]. In that study, screening was limited to three of 29 male prisons in the state, and frequent transfers likely diluted impact. Future work might examine how inter-facility movement and variation in screening practices across facilities affect intervention effectiveness. Finally, we did not model heterogeneity in infectiousness. If a small fraction of individuals accounts for most transmission, imperfect screening coverage may allow continued transmission. Collectively, these factors highlight the complexities of real-world implementation. Empirical studies evaluating repeated rounds of more frequent prison-wide screening are ongoing in Brazil; future work should compare modeled findings against empirical evidence and evaluate feasible strategies for operationalization under programmatic conditions.

Altogether, our findings support the prioritization of combined entry, exit, and biannual screening with CXR-CAD in Brazil, Colombia, and Peru, and similar settings. This strategy is projected to be highly cost-effective and impactful in reducing tuberculosis morbidity and transmission in prisons and population-wide. Screening should begin immediately with available methods while infrastructure is developed for more impactful and cost-effective tools like CXR-CAD. Ultimately, sustained investments in intensive, prison-wide screening—alongside structural reforms to reduce incarceration and prison crowding—will be essential to advancing regionally toward tuberculosis elimination.

## Supporting information

S1 Appendix Methodological details. **Table A. Key compartmental model parameters.** Parameters were calibrated, fixed, or sampled. For calibrated parameters, medians and 95% intervals are shown; for sampled parameters, sampling distributions are shown. For time-varying parameters (e.g., baseline mortality rate), values for 2026 are shown. Subscripts denote the incarceration-related population stratum for which a parameter applies; parameters without subscripts are assumed to be equivalent across strata. All rates are annualized. For further details, see Liu and colleagues *Lancet Public Health* 2024 [4]. **Table B. Additional parameters and cost inputs for base-case scenario and screening interventions.** All parameters were sampled from triangle distributions, for which the mode (range) is listed. All costs are in 2023 US dollars (USD). Note: costs are for individual components, not entire screening algorithms (e.g., the CXR-CAD algorithm includes a symptom interview and CXR-CAD, and for individuals who screen positive, clinical evaluation and Xpert Ultra). **Table C. Impacts of screening interventions on prison and population tuberculosis incidence in 2035.** Median estimates and 95% uncertainty intervals are shown for the projected percent reduction in prison or population incidence in 2035, relative to the base-case scenario. **Table D. Health benefits and costs of screening interventions.** Mean estimates and 95% uncertainty intervals for disability-adjusted life years (DALYs) averted, total costs, and additional costs relative to the base-case scenario over the 10-year intervention period. All estimates are standardized per 100,000 population. Costs are in 2023 US dollars. The “status” column indicates whether a strategy is on the cost-efficient frontier or dominated through strict dominance (D) or extended dominance (ED). **Table E. Optimal strategies by prison incidence.** Ranges of prison incidence under which each strategy has the highest probability of being the optimal strategy. N/A indicates that a given strategy was not optimal at any tested incidence level. **Table F. Costs, effects, and cost-effectiveness of strategies on the efficiency frontier if CXR-CAD were unavailable.** Mean estimates are shown, standardized per 100,000 population. All estimates are population-wide (i.e., they include costs and effects accrued by the entire population, not just those in prison). Only strategies on the efficiency frontier are shown; all other strategies were dominated. Costs are in 2023 US dollars. DALYs, disability-adjusted life years; increm., incremental; ICER, incremental cost-effectiveness ratio. **Table G. Costs of and DALYs averted by combined entry, exit and biannual screening with CXR-CAD, assuming declining test sensitivity.** Test sensitivity was reduced linearly during years 0–5 of the intervention period to levels consistent with asymptomatic disease, for both the screening step with CXR-CAD and follow-up testing with Xpert Ultra. Means and 95% uncertainty intervals are shown. Costs are in 2023 US dollars. **Fig A. Model fit to incarceration-related data targets.** Black points and error bars represent data targets and 95% uncertainty bounds (if applicable), respectively. Dark blue lines and shaded bands represent median model fits and 95% uncertainty intervals, respectively. In Brazil, recidivism data was only available for one year (2013); the calibration target and uncertainty bounds are shown by the vertical black and dotted lines, respectively. Incarc prev, incarceration prevalence; 100k, 100,000 population age 15+. **Fig B. Model fit to tuberculosis-related data targets.** Black points and error bars represent calibration targets and 95% uncertainty bounds (if applicable), respectively. Dark blue lines and shaded bands represent median model fits and 95% uncertainty intervals, respectively. “Combined” indicates population-wide notifications and incidence estimates. 100k, 100,000 person-years. **Fig C. Carceral characteristics and projected tuberculosis incidence in included countries.** Model projections for carceral characteristics at baseline and tuberculosis incidence in 2035 under the base-case scenario. Incarceration rates are for the population aged 15 and older. Recidivism refers to the proportion of people entering prison who have a prior incarceration history. **Fig D. Tuberculosis incidence over time under base-case and intervention scenarios**. Only interventions employing CXR-CAD are shown. Prison incidence is depicted in the top row; population-level incidence is in the bottom row. The shaded gray band shows the intervention period. **Fig E. Intervention costs, broken down by component.** Follow-up testing includes costs of culture and DST for people diagnosed with TB. Passive diagnosis costs indicate costs averted by screening interventions, relative to the base-case scenario. Error bars show 95% uncertainty intervals. Costs are in 2023 US dollars (USD). **Fig F. Proportion of true and false positives among treated individuals under each screening strategy.** Mean proportions are shown. **Fig G. Uncertainty in costs and DALYs averted of each screening strategy.** Costs are in 2023 US dollars (USD) and are additional relative to the base case scenario. 95% uncertainty ellipses are shown for each strategy. **Fig H. Cost-effectiveness acceptability curves and frontier.** Curves show the proportion of all iterations in which each strategy yielded the greatest net monetary benefit across a range of willingness-to-pay thresholds. Open squares indicate the cost-effectiveness acceptability frontier, comprised of the optimal strategy at each willingness-to-pay threshold (based on the expectation of net monetary benefit across iterations). The vertical dashed line indicates each country’s cost-effectiveness threshold. **Fig I. Cost-effectiveness plane without algorithms using CXR-CAD.** Strategies on the efficient frontier are highlighted. Costs (2023 USD) are additional relative to the base case scenario. DALYs, disability-adjusted life years.
(DOCX)

S1 CHEERS Checklist CHEERS 2022 Checklist. *From:* Husereau D, Drummond M, Augustovski F, et al. Consolidated Health Economic Evaluation Reporting Standards 2022 (CHEERS 2022) Explanation and Elaboration: A Report of the ISPOR CHEERS II Good Practices Task Force. Value Health 2022;25. https://doi.org/10.1016/j.jval.2021.10.008.
(DOCX)

## Abbreviations

CEACs: cost-effectiveness acceptability curves
CXR-CAD: chest X-ray with computer-aided detection
DALYs: disability-adjusted life years
DOT: directly observed treatment
DST: drug susceptibility testing
ICERs: incremental cost-effectiveness ratios
MDR: multi-drug resistant
PDL: people deprived of liberty
USD: US dollars
WHO: World Health Organization
YLDs: years lived with disability

## References

[1] WHO. Global tuberculosis report 2024. 2024.

[2] Walter KS, Martinez L, Arakaki-Sanchez D, Sequera VG, Estigarribia Sanabria G, Cohen T, et al. The escalating tuberculosis crisis in central and South American prisons. Lancet. 2021;397(10284):1591–6. doi: 10.1016/S0140-6736(20)32578-2 33838724 PMC9393884

[3] Martinez L, Warren JL, Harries AD, Croda J, Espinal MA, Olarte RAL, et al. Global, regional, and national estimates of tuberculosis incidence and case detection among incarcerated individuals from 2000 to 2019: a systematic analysis. Lancet Public Health. 2023;8(7):e511–9. doi: 10.1016/S2468-2667(23)00097-X 37393090 PMC10323309

[4] Liu YE, Mabene Y, Camelo S, Rueda ZV, Pelissari DM, Dockhorn Costa Johansen F, et al. Mass incarceration as a driver of the tuberculosis epidemic in Latin America and projected effects of policy alternatives: a mathematical modelling study. Lancet Public Health. 2024;9(11):e841–51. doi: 10.1016/S2468-2667(24)00192-0 39419058 PMC11602220

[5] Charalambous S, Velen K, Rueda Z, Croda J, Herce ME, Shenoi SV, et al. Scaling up evidence-based approaches to tuberculosis screening in prisons. Lancet Public Health. 2023;8(4):e305–10. doi: 10.1016/S2468-2667(23)00002-6 36780916

[6] World Health Organization. WHO consolidated guidelines on tuberculosis. module 2: screening - systematic screening for tuberculosis disease. 2021. 33822560

[7] Santos A da S, de Oliveira RD, Lemos EF, Lima F, Cohen T, Cords O, et al. Yield, efficiency, and costs of mass screening algorithms for tuberculosis in Brazilian prisons. Clin Infect Dis. 2021;72(5):771–7. doi: 10.1093/cid/ciaa135 32064514 PMC7935388

[8] Jung E, Alarcón V, Solís Tupes WS, Avalos-Cruz T, Tovar M, Abregu E, et al. National active case-finding program for tuberculosis in Prisons, Peru, 2024. Emerging Infectious Diseases. 2025;31(3):564. doi: 10.3201/eid3103.241727 40023810 PMC11878315

[9] Dos Santos PCP, da Silva Santos A, de Oliveira RD, da Silva BO, Soares TR, Martinez L, et al. Pooling sputum samples for efficient mass tuberculosis screening in prisons. Clin Infect Dis. 2022;74(12):2115–21. doi: 10.1093/cid/ciab847 34718459 PMC9258923

[10] Salindri AD, Bampi JVB, Busatto C, da Silva AM, da Silva Santos A, Gonçalves IB, et al. Evaluation of an ultra-portable X-ray system with automated interpretation for tuberculosis active case finding in carceral settings: a diagnostic test accuracy study. Res Sq. 2025;:rs.3.rs-5578367. doi: 10.21203/rs.3.rs-5578367/v1 41184804 PMC12581292

[11] Dara M, Chorgoliani D, de Colombani P. TB prevention and control care in prisons. World Health Organization/Europe Health in Prisons Programme; 2014.

[12] Mabud TS, de Lourdes Delgado Alves M, Ko AI, Basu S, Walter KS, Cohen T, et al. Evaluating strategies for control of tuberculosis in prisons and prevention of spillover into communities: an observational and modeling study from Brazil. PLoS Med. 2019;16(1):e1002737. doi: 10.1371/journal.pmed.1002737 30677013 PMC6345418

[13] Legrand J, Sanchez A, Le Pont F, Camacho L, Larouze B. Modeling the impact of tuberculosis control strategies in highly endemic overcrowded prisons. PLoS One. 2008;3(5):e2100. doi: 10.1371/journal.pone.0002100 18461123 PMC2324198

[14] Coussens AK, Zaidi SMA, Allwood BW, Dewan PK, Gray G, Kohli M, et al. Classification of early tuberculosis states to guide research for improved care and prevention: an international Delphi consensus exercise. Lancet Respir Med. 2024;12(6):484–98. doi: 10.1016/S2213-2600(24)00028-6 38527485 PMC7616323

[15] Menzies NA, Quaife M, Allwood BW, Byrne AL, Coussens AK, Harries AD, et al. Lifetime burden of disease due to incident tuberculosis: a global reappraisal including post-tuberculosis sequelae. Lancet Glob Health. 2021;9(12):e1679–87. doi: 10.1016/S2214-109X(21)00367-3 34798027 PMC8609280

[16] World Bank. GDP deflator (base year varies by country). 2023.

[17] Sanders GD, Neumann PJ, Basu A, Brock DW, Feeny D, Krahn M, et al. Recommendations for conduct, methodological practices, and reporting of cost-effectiveness analyses: second panel on cost-effectiveness in health and medicine. JAMA. 2016;316(10):1093–103. doi: 10.1001/jama.2016.12195 27623463

[18] Ministerio de Salud DGdM. Estimación del umbral costo - efectividad para las evaluaciones económicas en salud. Perú: Ministerio de Salud DGdM; 2022.

[19] Ministério da Saúde. O uso de limiares de custo-efetividade nas decisões em saúde: recomendações da Comissão Nacional de Incorporação de Tecnologias no SUS. In: Secretaria de Ciência T, Inovação e Insumos Estratégicos em Saúde; Departamento de Gestão e Incorporação de Tecnologias em Saúde, editor. Brasilia/DF, Brazil; 2022.

[20] Espinosa O, Rodríguez-Lesmes P, Orozco L, Ávila D, Enríquez H, Romano G, et al. Estimating cost-effectiveness thresholds under a managed healthcare system: experiences from Colombia. Health Policy Plan. 2022;37(3):359–68. doi: 10.1093/heapol/czab146 34875689

[21] Barton GR, Briggs AH, Fenwick EAL. Optimal cost-effectiveness decisions: the role of the cost-effectiveness acceptability curve (CEAC), the cost-effectiveness acceptability frontier (CEAF), and the expected value of perfection information (EVPI). Value Health. 2008;11(5):886–97. doi: 10.1111/j.1524-4733.2008.00358.x 18489513

[22] Soares TR, Oliveira RDde, Liu YE, Santos A da S, Santos PCPD, Monte LRS, et al. Evaluation of chest X-ray with automated interpretation algorithms for mass tuberculosis screening in prisons: a cross-sectional study. Lancet Reg Health Am. 2022;17:100388. doi: 10.1016/j.lana.2022.100388 36776567 PMC9904090

[23] Machekera SM, Wilkinson E, Hinderaker SG, Mabhala M, Zishiri C, Ncube RT, et al. A comparison of the yield and relative cost of active tuberculosis case-finding algorithms in Zimbabwe. Public Health Action. 2019;9(2):63–8. doi: 10.5588/pha.18.0098 31417855 PMC6645451

[24] Smit GSA, Apers L, Arrazola de Onate W, Beutels P, Dorny P, Forier A-M, et al. Cost-effectiveness of screening for active cases of tuberculosis in Flanders, Belgium. Bull World Health Organ. 2017;95(1):27–35. doi: 10.2471/BLT.16.169383 28053362 PMC5180339

[25] Winetsky DE, Negoescu DM, DeMarchis EH, Almukhamedova O, Dooronbekova A, Pulatov D, et al. Screening and rapid molecular diagnosis of tuberculosis in prisons in Russia and Eastern Europe: a cost-effectiveness analysis. PLoS Med. 2012;9(11):e1001348. doi: 10.1371/journal.pmed.1001348 23209384 PMC3507963

[26] Sequera G, Estigarribia-Sanabria G, Aguirre S, Piñanez C, Martinez L, Lopez-Olarte R, et al. Excess tuberculosis risk during and following incarceration in Paraguay: a retrospective cohort study. Lancet Reg Health Am. 2024;31:100668. doi: 10.1016/j.lana.2023.100668 38500958 PMC10945421

[27] Urrego J, Ko AI, da Silva Santos Carbone A, Paião DSG, Sgarbi RVE, Yeckel CW, et al. The impact of ventilation and early diagnosis on tuberculosis transmission in Brazilian prisons. Am J Trop Med Hyg. 2015;93(4):739–46. doi: 10.4269/ajtmh.15-0166 26195459 PMC4596592

[28] Narayan A, Salindri AD, Keshavjee S, Muyoyeta M, Velen K, Rueda ZV, et al. Prioritizing persons deprived of liberty in global guidelines for tuberculosis preventive treatment. PLoS Med. 2023;20(10):e1004288. doi: 10.1371/journal.pmed.1004288 37788448 PMC10547494

[29] García PJ, Bayer A, Cárcamo CP. The changing face of HIV in Latin America and the Caribbean. Curr HIV/AIDS Rep. 2014;11(2):146–57. doi: 10.1007/s11904-014-0204-1 24824881 PMC4136548

[30] Pivetta de Araujo RC, Martinez L, da Silva Santos A, Ferreira Lemos E, Dias de Oliveira R, Croda M, et al. Serial Mass Screening for Tuberculosis Among Incarcerated Persons in Brazil. Clin Infect Dis. 2024;78(6):1669–76. doi: 10.1093/cid/ciae055 38324908 PMC11175667

[31] Pelissari DM, Kuhleis DC, Bartholomay P, Barreira D, Oliveira CLP, de Jesus RS, et al. Prevalence and screening of active tuberculosis in a prison in the South of Brazil. Int J Tuberc Lung Dis. 2018;22(10):1166–71. doi: 10.5588/ijtld.17.0526 30236184

[32] Telisinghe L, Fielding KL, Malden JL, Hanifa Y, Churchyard GJ, Grant AD, et al. High tuberculosis prevalence in a South African prison: the need for routine tuberculosis screening. PLoS One. 2014;9(1):e87262. doi: 10.1371/journal.pone.0087262 24498059 PMC3907552

[33] Van’t Hoog A, Viney K, Biermann O, Yang B, Leeflang MM, Langendam MW. Symptom- and chest-radiography screening for active pulmonary tuberculosis in HIV-negative adults and adults with unknown HIV status. Cochrane Database Syst Rev. 2022;3(3):CD010890. doi: 10.1002/14651858.CD010890.pub2 35320584 PMC9109771

[34] dos Santos PCP, da Silva Santos A, de Oliveira RD, da Silva BO, Soares TR, Martinez L, et al. Pooling sputum samples for efficient mass tuberculosis screening in prisons. Clin Infect Dis. 2021;74(12):2115–21. doi: 10.1093/cid/ciab847 PMC9258923 34718459

[35] Shapiro AE, Ross JM, Yao M, Schiller I, Kohli M, Dendukuri N, et al. Xpert MTB/RIF and Xpert Ultra assays for screening for pulmonary tuberculosis and rifampicin resistance in adults, irrespective of signs or symptoms. Cochrane Database Syst Rev. 2021;3(3):CD013694. doi: 10.1002/14651858.CD013694.pub2 33755189 PMC8437892

[36] Zifodya JS, Kreniske JS, Schiller I, Kohli M, Dendukuri N, Schumacher SG, et al. Xpert Ultra versus Xpert MTB/RIF for pulmonary tuberculosis and rifampicin resistance in adults with presumptive pulmonary tuberculosis. Cochrane Database Syst Rev. 2021;2(2):CD009593. doi: 10.1002/14651858.CD009593.pub5 33616229 PMC12045032

[37] IHME. Global Burden of Disease Study 2019 disability weights. 2021.

[38] Ragonnet R, Flegg JA, Brilleman SL, Tiemersma EW, Melsew YA, McBryde ES, et al. Revisiting the natural history of pulmonary tuberculosis: a bayesian estimation of natural recovery and mortality rates. Clin Infect Dis. 2021;73(1):e88–96. doi: 10.1093/cid/ciaa602 32766718

[39] World Health Organization. Life tables by country. Global Health Observatory data repository. 2020.

