# Supplementary material for: Comparative impacts and cost-effectiveness of tuberculosis systematic screening strategies in prisons in Brazil, Colombia, and Peru: A mathematical modeling study
Source: PLoS Med. 2026 Jun 4;23(6):e1004739. doi: 10.1371/journal.pmed.1004739 (PMC13258141; doi:10.1371/journal.pmed.1004739)
Supplement: S1 Appendix — Table A. Key compartmental model parameters. Parameters were calibrated, fixed, or sampled. For calibrated parameters, medians and 95% intervals are shown; for sampled parameters, sampling distributions are shown. For time-varying parameters (e.g., baseline mortality rate), values for 2026 are shown. Subscripts denote the incarceration-related population stratum for which a parameter applies; parameters without subscripts are assumed to be equivalent across strata. All rates are annualized. For further details, see Liu and colleagues Lancet Public Health 2024 [4]. Table B. Additional parameters and cost inputs for base-case scenario and screening interventions. All parameters were sampled from triangle distributions, for which the mode (range) is listed. All costs are in 2023 US dollars (USD). Note: costs are for individual components, not entire screening algorithms (e.g., the CXR-CAD algorithm includes a symptom interview and CXR-CAD, and for individuals who screen positive, clinical evaluation and Xpert Ultra). Table C. Impacts of screening interventions on prison and population tuberculosis incidence in 2035. Median estimates and 95% uncertainty intervals are shown for the projected percent reduction in prison or population incidence in 2035, relative to the base-case scenario. Table D. Health benefits and costs of screening interventions. Mean estimates and 95% uncertainty intervals for disability-adjusted life years (DALYs) averted, total costs, and additional costs relative to the base-case scenario over the 10-year intervention period. All estimates are standardized per 100,000 population. Costs are in 2023 US dollars. The “status” column indicates whether a strategy is on the cost-efficient frontier or dominated through strict dominance (D) or extended dominance (ED). Table E. Optimal strategies by prison incidence. Ranges of prison incidence under which each strategy has the highest probability of being the optimal strategy. N/A indicates that a given stra [file pmed.1004739.s001.docx]

**S1 Appendix**

**Supplement to: Comparative impacts and cost-effectiveness of tuberculosis active case-finding strategies in prisons in Brazil, Colombia, and Peru: a mathematical modeling study**

**Table of Contents**

**Methodological Details**

**Supplementary Tables**

Table A. Key compartmental model parameters.

Table B. Additional parameters and cost inputs for base-case scenario and screening interventions.

Table C. Impacts of screening interventions on prison and population TB incidence in 2035.

Table D. Health benefits and costs of screening interventions.

Table E. Optimal strategies by prison incidence.

Table F. Costs, effects, and cost-effectiveness of strategies on the efficiency frontier if CXR-CAD were unavailable.

Table G. Costs of and DALYs averted by combined entry, exit, and biannual screening with CXR-CAD, assuming declining test sensitivity.

**Supplementary Figures**

Fig A. Model fit to incarceration-related data targets.

Fig B. Model fit to tuberculosis-related data targets.

Fig C. Carceral characteristics and projected tuberculosis incidence in included countries.

Fig D. Tuberculosis incidence over time under base-case and intervention scenarios.

Fig E. Intervention costs, broken down by component.

Fig F. Proportion of true and false positives among treated individuals under each screening strategy.

Fig G. Uncertainty in costs and DALYs averted of each screening strategy.

Fig H. Cost-effectiveness acceptability curves and frontier.

Fig I. Cost-effectiveness plane without algorithms using CXR-CAD.

**Methodological Details**

*Model calibration*

Calibration targets for within-prison tuberculosis incidence were based on a recent study using Bayesian meta-regression modeling to generate national and regional estimates of prison incidence and the case detection ratio (CDR)[1]. For Brazil and Colombia, where country-specific data were included from active-case finding studies in prisons, we directly used posterior distributions of prison incidence from this study. For Peru, where no such active case-finding studies had been conducted (prior to 2023), we estimated the uncertainty distribution for the prison incidence calibration target by applying a regional CDR of 0.54 (95% UI [0.20, 0.96]) to Peru-specific prison notifications data.

The model was calibrated with *optim* in R using limited memory BFGS, a quasi-Newton algorithm, with box constraints. Incarceration-related parameters were calibrated first with a loss function that minimized the mean squared percentage error across incarceration prevalence, recidivism, and admissions. Remaining parameters were calibrated by minimizing the mean percentage error across prison TB incidence, population TB incidence, prison TB notifications, and population TB notifications. Calibrated parameters are indicated in **Table A in S1 Appendix**. Additional details on model calibration are provided in Liu et al. *Lancet Public Health* 2024[2], from which calibrated parameters were sourced directly.

*Probability of treatment success during and after incarceration*

To determine probability of treatment success, we consider probability of treatment completion as well as culture conversion among patients who do not complete the full treatment regimen, assuming that one third of patients who do not complete treatment are nonetheless cured[3]. A recent analysis in Mato Grosso do Sul, Brazil found that 67% of individuals newly diagnosed with tuberculosis during incarceration complete treatment[4]. Accounting for cure without full treatment completion results in a 78% probability of treatment success for individuals diagnosed during incarceration. The same analysis found that 61% of individuals diagnosed within two years post-release complete treatment, which we assume to be comparable for individuals diagnosed through exit screening. Accounting for possible cure without full treatment completion results in 74% treatment success for individuals diagnosed through exit screening.

*Impacts of COVID-19 pandemic*

Prior assumptions about the impact of the COVID-19 pandemic on tuberculosis were updated based on newly available data. Specifically, we assumed that tuberculosis diagnosis rates were less impacted in prisons than in the community[5], and that return to pre-COVID-19 pandemic diagnosis rates in all strata would occur gradually over several years.

*Passive diagnosis*

To estimate usage of various diagnostic methods under the base-case scenario, we use data from the WHO Global TB Report (and for Brazil, the Information System for Notifiable Diseases SINAN). We assume that patients undergo clinical evaluation and receive any combination of smear, molecular rapid diagnostics (i.e. GeneXpert), culture, and drug sensitivity testing, with the proportion of patients receiving each assay sampled from independent distributions.

To estimate costs of passive diagnosis and treatment, we estimated, per person cured: 1) the number of people evaluated for tuberculosis, 2) the number of people diagnosed with tuberculosis, and 3) the number of people initiated on treatment. According to data from the WHO Global TB Report 2023 for Brazil, 12% of individuals initially tested with a WHO-recommended rapid diagnostic test (WRD) had a positive test result, and 6.2 WRDs were used per person notified as a tuberculosis case. Since WRDs are not used for everyone who is evaluated (45% of people newly diagnosed with tuberculosis in Brazil in 2022 were initially tested with a WRD), the number of individuals evaluated per person diagnosed is likely higher. We therefore assume approximately 10 (range 5-15) individuals are evaluated for tuberculosis per person diagnosed. To adjust this ratio to be a function of the number of people evaluated per person cured, we use a recent study on the tuberculosis care cascade in Brazil which showed that among individuals diagnosed with tuberculosis, 90% initiate treatment and 81.4% are cured[6]. We assume the same ratios across countries.

*Systematic screening interventions*

Periodic prison-wide screening (annual or biannual) was implemented as a discrete pulse process, with screening activated during rounds of fixed duration (assumed to be two months each) and deactivated between rounds. For annual screening, one round occurs per year; for biannual screening, two rounds occur per year, with the start of each round spaced six months apart. Other than frequency of rounds, screening rates and per-round coverage and duration are assumed to be identical between annual and biannual screening.

Entry screening is implemented as a modifier on the flow of individuals entering prison. A fraction of infectious individuals entering prison (from community strata) are detected and treated at the point of entry, transitioning directly to $R_{p}.$ Entry screening also moves a fraction of individuals entering prison from $E$ compartments directly to $L_{p}$. Screening of individuals transferred between prisons is modeled analogously, as a continuous rate proportional to the transfer rate.

Exit screening is implemented similarly as a modifier on the flow of individuals leaving prison, with detected and treated individuals transitioning directly from $I_{p}$ to $R_{r}$, and from $E_{p}$ to $L_{r}$.

*False positives*

Our screening test accuracy parameters (sensitivity and specificity) are derived from empirical studies using bacteriologically confirmed active tuberculosis as the reference standard. Therefore, we define a false positive as any individual without active, infectious tuberculosis disease (compartment $I$) who tests positive through systematic screening, including individuals with latent infection.

Detecting and treating individuals in $E$ could confer an unintended benefit by clearing early latent infection. In the model, treated false positives in $E$ transition to $L$, reflecting the effect of completing a course of treatment for active disease. We chose to transition these individuals to $L$ rather than $R$ because, in our model structure, individuals in $R$ can relapse to $I$ without reinfection, which would not be appropriate for individuals who never developed active disease. Moving treated $E$ individuals to $L$ captures the benefit of reduced activation risk while avoiding this structural inconsistency.

*Test accuracy*

For each screening or diagnostic method (cough, CXR-CAD, Xpert Ultra), we simulated joint uncertainty distributions for sensitivity and specificity, with induced correlation of -0.5 to account for their inverse dependency.

*Costs*

Country-specific costs were not available for Colombia and, in some cases, Peru. For these costs, we borrowed parameters from Brazil, assuming the same cost of consumables and applying an adjustment factor for labor costs based on ratios of GDP per capita.

*Cost-effectiveness analysis*

Cost-effectiveness thresholds were drawn from recent economic evaluations commissioned by ministries of health in each of the selected countries and were similar to the crude estimate of GDP per capita often used for low- and middle-income countries[7-9]. Thresholds were updated for 2023 using time trends in GDP per capita. We did not project future healthcare costs beyond the ten-year analytic horizon, nor account for future infection, disease, or transmission occurring after the end of this period.

*Sensitivity analyses*

For the first sensitivity analysis, we generated a wide range of within-prison tuberculosis incidence values by multiplying the previously calibrated effective contact rate in prison by a sampled factor 0.1 < *f* < 1 for each of the 1000 parameter sets per country. A constraint was then imposed to ensure that the prison effective contact rate in prison remained greater than or equal to the community effective contact rate. After re-simulating screening interventions, we identified the optimal strategy, defined as the most impactful, non-dominated strategy with an incremental cost-effectiveness ratio (ICER) within the country’s cost-effectiveness threshold. For each country, using the results from 1000 model simulations, we fit a multinomial logistic regression model using multinom from the nnet package in R[10], with the optimal strategy as the dependent variable and within-prison incidence as the predictor variable. Each observation corresponded to one simulation (i.e., one parameter set with its associated prison incidence level and identified optimal strategy), yielding 1,000 observations per country. We then used this model to predict the probability of each strategy being optimal across the incidence range.

For the third sensitivity analysis, we focused on one intensive screening strategy: the combination of entry, exit, and biannual screening with CXR-CAD. To represent the shift toward less severe—and less detectable—TB disease over time, we allowed test sensitivity to decline linearly over the first five years of the intervention period, eventually reaching sensitivity values consistent with asymptomatic or subclinical TB. By year five, sensitivity of CXR-CAD screening declined to Triangle(62.6%, 71.2%, 67.7%), and sensitivity of Xpert Ultra follow-up testing declined to Triangle(67.6%, 85.6%, 77.5%)[11]. Quantile rank was preserved between sampled start and end values. We assumed that sensitivity decreased for all screening points (entry, exit, and periodic screening).

Each sensitivity analysis was conducted with 1000 sets of model parameters and inputs, combining uncertainty from existing model parameters and inputs with additional sampled parameters for the sensitivity variable(s) of interest. This ensured that uncertainty was propagated throughout the sensitivity analyses.

**Table A. Key compartmental model parameters.** Parameters were calibrated, fixed, or sampled. For calibrated parameters, medians and 95% intervals are shown; for sampled parameters, sampling distributions are shown. For time-varying parameters (e.g. baseline mortality rate), values for 2026 are shown. Subscripts denote the incarceration-related population stratum for which a parameter applies; parameters without subscripts are assumed to be equivalent across strata. All rates are annualized. For further details, see Liu et al. *Lancet Public Health* 2024[2].

| Parameter | Description | Brazil | Colombia | Peru | Source |
| --- | --- | --- | --- | --- | --- |
| $q_{r}$ | Entry rate into prison from **r** stratum | 0.24 (0.15-0.35) | 0.034 (0.018-0.05) | 0.057 (0.033-0.085) | Calibrated |
| $q_{d} , q_{n}$ | Entry rate into prison from **d** and **n** strata | 0.0016 (0.0011-0.0022) | 0.00085 (0.00069-0.0010) | 0.00078 (0.00063-0.00095) | Calibrated |
| $r$ | Release rate from prison | 0.78 (0.63-0.93) | 0.31 (0.25-0.93) | 0.20 (0.16-0.25) | Calibrated |
| $\omega$ | Rate of transition from **r** stratum to **d** stratum | 0.143 | | | [12, 13] |
| $\beta_{pp}$ | Effective contact rate for within-prison transmission | 62.8 (47.4-77.1) | 27.1 (16.5-40.0) | 75.0 (56.5-97.8) | Calibrated |
| $\beta_{cc}$ | Effective contact rate for transmission among individuals outside prison | 5.0 (3.0-8.8) | 11.2 (6.3-18.7) | 6.5 (3.0-14.0) | Calibrated |
| $\beta_{pc}$ | Effective contact rate for incarcerated individual exposed to non-incarcerated individual | Tri(0.25, 0.75, 0.5) | | | Assumed |
| $\beta_{cp}$ | Effective contact rate for non-incarcerated individual exposed to incarcerated individual | Calculated as $\beta_{pc}\frac{N_{p}}{N_{c}}$ | | | Calculated |
| $\tau_{p}$ | Fast progression rate from early latent to infectious in prison | 0.13 (0.10-0.15) | 0.12 (0.091-0.16) | 0.15 (0.11-0.20) | Calibrated |
| $\tau_{d} ,\tau_{n}$ | Fast progression rate from early latent to infectious in **d** and **n** strata | 0.060 (0.039-0.090) | 0.040 (0.020-0.076) | 0.093 (0.061-0.13) | Calibrated |
| $\tau_{r}$ | Fast progression rate from early latent to infectious in **r** stratum; calculated as weighted average of $\tau_{p}$ and $\tau_{d} ,\tau_{n}$ | Weight ~ Unif(0, 1) | | | Assumed |
| $\delta_{d} , \delta_{n}$ | Diagnosis rate in **d** and **n** strata | 1.9 (1.2-2.4) | 1.3 (0.7-1.8) | 1.9 (1.2-3.0) | Calibrated |
| $\delta_{p}$ | Diagnosis rate in prison | 1.2 (0.7-1.2) | 0.6 (0.2-1.0) | 0.7 (0.4-1.2) | Calibrated |
| $\delta_{r}$ | Diagnosis rate in **r** stratum; calculated as weighted average of $\delta_{p}$ and $\delta_{d} , \delta_{n}$ | Weight ~ Unif(0, 1) | | | Assumed |
| $b$ | Transition rate from early to late latent | Tri(0.75, 1, 0.875) | | | [14] |
| $e$ | Slow progression rate from late latent to infectious | 0.000594 | | | [14] |
| $\alpha$ | Relative risk of reinfection for late latent and recovered individuals | Lognormal(-1.56, 0.03) | | | [15] |
| $\gamma$ | Rate of relapse from R to I | 0.01 | | | [16] |
| ${mu}_{TB}$ | TB mortality rate, assuming 10-50% prevalence of smear-positive TB | Unif(0.061, 0.21) | | | [17] |
| $\sigma$ | Self-cure rate | Unif(0.14, 0.18) | | | [17] |
| ${mu}_{d} , {mu}_{n}$ | Mortality rate in **d** and **n** strata | 0.0160 | 0.0156 | 0.0157 | [18] |
| ${mu}_{p}$ | Mortality rate in prison; calculated as rate ratio of ${mu}_{d} , {mu}_{n}$ | Rate ratio ~ $N$(0.65, 0.11) | | | [13] |
| ${mu}_{r}$ | Mortality rate in **r** stratum; calculated as rate ratio of ${mu}_{d} , {mu}_{n}$ | Rate ratio ~ $N$(1.05, 0.18) | | | [13] |

**Table B. Additional parameters and cost inputs for base-case scenario and screening interventions.** All parameters were sampled from triangle distributions, for which the mode (range) is listed. All costs are in 2023 US dollars (USD). Note: costs are for individual components, not entire screening algorithms (e.g., the CXR-CAD algorithm includes a symptom interview and CXR-CAD, and for individuals who screen positive, clinical evaluation and Xpert Ultra).

| Parameter | Country | Value (Range) | Source |
| --- | --- | --- | --- |
| Base case scenario (passive diagnosis) | | | |
| Proportion evaluated with GeneXpert | Brazil | 0.44 (0.4-0.5) | [19, 20] |
|  | Colombia | 0.53 (0.45-0.65) | ^1^ |
|  | Peru | 0.3 (0.25-0.4) | ^1^ |
| Proportion evaluated with smear | Brazil | 0.7 (0.6-0.8) | [19, 20] |
|  | Colombia | 0.81 (0.7-0.9) | ^1^ |
|  | Peru | 0.8 (0.7-0.9) | ^1^ |
| Proportion of patients receiving culture | Brazil | 0.45 (0.4-0.5) | [19, 20] |
|  | Colombia | 0.42 (0.35-0.5) | ^1^ |
|  | Peru | 0.62 (0.5-0.75) | ^1^ |
| Proportion of patients receiving drug susceptibility testing | Brazil | 0.25 (0.1-0.33) | [19, 20] |
|  | Colombia | 0.6 (0.4-0.7) | ^1^ |
|  | Peru | 0.69 (0.6-0.8) | ^1^ |
| Average number of people evaluated per person cured | All | 12.29 (6.14-18.43) | [6, 19] |
| Average number of people diagnosed per person cured | All | 1.23 (1.17-1.31) | [6] |
| Average number of people initiated on treatment per person cured | All | 1.11 (1.05-1.2) | [6] |
| Screening interventions | | | |
| Screening coverage | All | 0.8 (0.7-0.9) | [21-23] |
| Duration of screening round for periodic screening (years) | All | 0.167 (0.083-0.25) | Assumed |
| Probability of DS-TB treatment completion in prison for individuals detected through ACF | All | 0.67 (0.6-0.75) | [4] |
| Probability of linkage to care post-release for individuals detected through exit screening | All | 0.65 (0.5-0.75) | Assumed |
| Probability of DS-TB treatment completion post-release following linkage to care | All | 0.61 (0.5-0.7) | Unpublished data from Brazil |
| Proportion of TB patients with RR-TB, general population | Brazil | 0.016 (0.006-0.025) | [19] |
|  | Colombia | 0.045 (0.02-0.06) | [19] |
|  | Peru | 0.0726 (0.06-0.085) | [24] |
| Proportion of TB patients with RR-TB, prison | Brazil | 0.016 (0.006-0.025) | Assumed same as general population |
|  | Colombia | 0.045 (0.02-0.06) | Assumed same as general population |
|  | Peru | 0.093 (0.08-0.12) | [24] |
| Probability of detection and treatment completion for individuals with RR-TB detected through ACF, relative to individuals with DS-TB | All | 0.4875 (0.4-0.6) | [19] |
| Cost-effectiveness analysis | | | |
| Per-unit cost of smear microscopy | Brazil | 3.9 (2-6) | [25] |
|  | Colombia | 3.01 (1.54-4.63) | [25] |
|  | Peru | 2.14 (1.5-5) | [26] |
| Per-unit cost of culture | Brazil | 10.43 (8-22) | [25] |
|  | Colombia | 8.84 (6.17-16.97) | [25] |
|  | Peru | 9.26 (7.1-19.53) | [25] |
| Per-unit cost of drug susceptibility testing (assuming 50/50 LPA vs. culture-based testing) | Brazil | 111.1 (55-165) | [27] |
|  | Colombia | 94.15 (42.42-127.25) | [27] |
|  | Peru | 154.49 (77.24-231.73) | [28] |
| Per-unit cost of Xpert MTB/RIF Ultra, accounting for recent cartridge price reduction | Brazil | 18.99 (17-35) | [21] |
|  | Colombia | 16.86 (14.25-26.75) | [21] |
|  | Peru | 17.42 (14.97-28.93) | [21] |
| Per-unit additional cost of pooled Xpert (50% additional human resources) | Brazil | 1.72 (1.5-2) | [21] |
|  | Colombia | 1.20 (1.04-1.39) | [21] |
|  | Peru | 1.33 (1.16-1.55) | [21] |
| Per-unit cost of CXR-CAD | Brazil | 7.5 (6-11) | [21] |
|  | Colombia | 6.83 (5.8-10) | [21] |
|  | Peru | 7.01 (5.5-10) | [21] |
| Per-unit cost of symptom interview | Brazil | 2.27 (2-3) | [21] |
|  | Colombia | 1.58 (1.39-2.08) | [21] |
|  | Peru | 1.76 (1.5-2.5) | [21] |
| Per-unit cost of clinical evaluation | Brazil | 3.11 (2-5) | [21] |
|  | Colombia | 2.16 (1.8-5) | [21] |
|  | Peru | 2.41 (2-6) | [21] |
| Cost of drug-sensitive treatment | Brazil | 567 (460-800) | [27] |
|  | Colombia | 497.81 (403.87-702.38) | [27] |
|  | Peru | 540.71 (440-800) | [28] |
| Cost of RR-TB treatment (assuming 50/50 standard 18-month regimen vs. BPaLM) | Brazil | 5301.5 (2650-8000) | [27] |
|  | Colombia | 4816.32 (2407.48-7267.86) | [27] |
|  | Peru | 5098.15 (3000-8000) | [28] |
| Relative cost of incomplete treatment | All | 0.5 | Assumed |

**Table C. Impacts of screening interventions on prison and population tuberculosis incidence in 2035.** Median estimates and 95% uncertainty intervals are shown for the projected percent reduction in prison or population incidence in 2035, relative to the base-case scenario.

| Country | Intervention | Population | Symptoms | CXR-CAD | Symptoms + CXR-CAD | Pooled Xpert |
| --- | --- | --- | --- | --- | --- | --- |
| Brazil | Entry | Prison | 13.7 (8.6-21.1) | 22 (15.3-30.4) | 23.8 (16.8-32.4) | 17.6 (12.1-24.9) |
|  |  | Population | 5.4 (3.2-8.6) | 8.7 (5.8-12.9) | 9.4 (6.3-13.9) | 7 (4.6-10.5) |
|  | Exit | Prison | 1.1 (0.5-2) | 1.7 (0.9-3) | 1.9 (1-3.4) | 1.4 (0.7-2.4) |
|  |  | Population | 0.7 (0.4-1.2) | 1.1 (0.7-1.8) | 1.2 (0.8-2) | 0.9 (0.5-1.5) |
|  | Annual | Prison | 20.7 (13.6-30.5) | 35.3 (27.9-43.8) | 38.5 (30.6-47.3) | 27.4 (21.2-35.2) |
|  |  | Population | 8.2 (5.1-12.7) | 13.8 (10.2-18.7) | 15 (11.2-20.1) | 10.8 (7.8-15) |
|  | Biannual | Prison | 39.2 (26.4-54.7) | 61.9 (52-71.9) | 66.2 (56.2-75.6) | 50.3 (40.6-61.7) |
|  |  | Population | 15.3 (9.8-22.6) | 24 (18.1-31.2) | 25.6 (19.5-33) | 19.5 (14.4-26.2) |
|  | Entry + exit + annual | Prison | 33.6 (22.4-47.3) | 53 (42.8-63.5) | 56.9 (46.8-66.9) | 42.8 (33.1-53.6) |
|  |  | Population | 13.3 (8.3-19.7) | 20.7 (15.3-27.7) | 22.1 (16.7-29.4) | 16.8 (12.2-23.3) |
|  | Entry + exit + biannual | Prison | 50.2 (34.4-66.9) | 73.5 (63.6-81.9) | 77.1 (67.9-84.4) | 62.4 (51.2-73.1) |
|  |  | Population | 19.6 (12.7-28.2) | 28.4 (21.6-36.5) | 29.7 (22.7-38) | 24.1 (18-32) |
| Colombia | Entry | Prison | 19.3 (10.3-25.5) | 28.3 (21.8-39.8) | 30.6 (24.3-42.8) | 23 (17-32.3) |
|  |  | Population | 3.3 (2.3-6.6) | 5.5 (4.4-10.4) | 5.8 (4.8-11.1) | 4.5 (3.6-8.5) |
|  | Exit | Prison | 0.1 (0.1-0.3) | 0.2 (0.1-0.5) | 0.3 (0.2-0.5) | 0.2 (0.1-0.4) |
|  |  | Population | 0.3 (0.1-0.6) | 0.5 (0.3-1) | 0.5 (0.3-1.1) | 0.4 (0.2-0.8) |
|  | Annual | Prison | 37.5 (23.2-48.4) | 57.6 (50.1-66.4) | 62.7 (54.8-70.3) | 48.1 (37-56) |
|  |  | Population | 6.8 (4.4-11.2) | 10.7 (8.9-17.4) | 11.6 (9.6-18.4) | 8.5 (6.9-14.4) |
|  | Biannual | Prison | 62.1 (42.6-75.2) | 82.8 (76.2-88.1) | 85.2 (80.1-90) | 74.4 (62.3-81.4) |
|  |  | Population | 11.3 (8-17.8) | 15.6 (12.8-23) | 16.3 (13.2-23.7) | 13.3 (11.4-21) |
|  | Entry + exit + annual | Prison | 51.3 (33.8-62.4) | 69.9 (65.6-80.5) | 73.7 (69.7-83.3) | 61 (52-71.5) |
|  |  | Population | 9.1 (6.6-15.5) | 13.7 (11.2-21.1) | 14.5 (11.8-21.9) | 11.2 (9.5-18.7) |
|  | Entry + exit + biannual | Prison | 70.4 (51.5-81.5) | 86.5 (82.7-91.7) | 88.9 (85.5-92.9) | 80.2 (71.2-87) |
|  |  | Population | 12.7 (9.7-20.2) | 16.9 (13.6-24) | 17.4 (13.8-24.5) | 14.9 (12.6-22.7) |
| Peru | Entry | Prison | 8.9 (4.7-14) | 14.1 (8.8-20.8) | 15.2 (9.6-22.1) | 11.4 (7-16.7) |
|  |  | Population | 2.9 (1.7-4.7) | 4.6 (3-6.7) | 5 (3.3-7.2) | 3.7 (2.4-5.5) |
|  | Exit | Prison | 0.1 (0-0.1) | 0.1 (0-0.2) | 0.1 (0-0.2) | 0.1 (0-0.2) |
|  |  | Population | 0.2 (0.1-0.6) | 0.4 (0.2-0.9) | 0.4 (0.2-1) | 0.3 (0.1-0.8) |
|  | Annual | Prison | 19.1 (13-28.1) | 32 (24.5-38.9) | 34.9 (26.8-41.3) | 25.3 (19.1-32) |
|  |  | Population | 6.2 (4.1-9.8) | 10.7 (7.8-13.8) | 11.8 (8.3-14.8) | 8.3 (6.2-11.2) |
|  | Biannual | Prison | 35.3 (25.3-48.6) | 55 (46.4-62.4) | 58.2 (50.4-66) | 45.3 (38.2-53.7) |
|  |  | Population | 11.8 (7.8-18) | 18.7 (12.8-24.6) | 20 (13.7-26.2) | 15.1 (10.5-20.9) |
|  | Entry + exit + annual | Prison | 27.1 (18.1-37.7) | 42.3 (34.8-50) | 45.5 (37.1-53) | 34.2 (27.7-42.3) |
|  |  | Population | 9 (6-13.7) | 14.3 (10.2-19) | 15.5 (11-20.1) | 11.6 (8.1-15.8) |
|  | Entry + exit + biannual | Prison | 42 (29.9-54.9) | 61.4 (52.9-69.6) | 64.6 (56.7-72.8) | 52 (43.4-60.5) |
|  |  | Population | 14 (9.6-21) | 20.6 (14.6-27.4) | 21.9 (15.4-28.8) | 17.4 (12-24) |

**Table D. Health benefits and costs of screening interventions.** Mean estimates and 95% uncertainty intervals for disability-adjusted life years (DALYs) averted, total costs, and additional costs relative to the base-case scenario over the ten-year intervention period. All estimates are standardized per 100,000 population. Costs are in 2023 US dollars. The “status” column indicates whether a strategy is on the cost-efficient frontier or dominated through strict dominance (D) or extended dominance (ED).

| Intervention | Algorithm | DALYs averted | Total costs | Additional costs relative to base case scenario | Status |
| --- | --- | --- | --- | --- | --- |
| Brazil | | | | |  |
| Base case | N/A | 0 | 304676 (244051-371439) | 0 | Frontier |
| Entry | Symptoms | 54.3 (23.9-112.5) | 360330 (291465-433634) | 55654 (36685-79699) | D |
|  | CXR-CAD | 84.5 (40.9-166.3) | 369771 (304744-439093) | 65095 (44548-91997) | D |
|  | Symptoms + CXR-CAD | 90.4 (43.9-176.7) | 420869 (344571-501427) | 116193 (83416-155554) | D |
|  | Pooled Xpert | 68.9 (32.6-137.9) | 390365 (318286-473837) | 85689 (56425-127858) | D |
| Exit | Symptoms | 15.4 (6-35.6) | 339512 (273935-408911) | 34836 (23244-49967) | ED |
|  | CXR-CAD | 24.9 (10.8-56.9) | 350021 (285794-419018) | 45344 (32170-62447) | D |
|  | Symptoms + CXR-CAD | 26.9 (11.7-62.2) | 377543 (306376-452778) | 72867 (52846-98408) | D |
|  | Pooled Xpert | 19.8 (8.4-44.7) | 362944 (292595-440935) | 58268 (38856-85934) | D |
| Annual | Symptoms | 82.3 (37.6-160.2) | 340920 (275923-408570) | 36244 (21106-56101) | ED |
|  | CXR-CAD | 131.8 (65.2-252.4) | 342428 (281164-406959) | 37752 (20646-60792) | Frontier |
|  | Symptoms + CXR-CAD | 141.7 (70.9-270.2) | 381612 (315921-451025) | 76936 (52247-109496) | D |
|  | Pooled Xpert | 105.9 (51.3-204) | 362100 (295456-435139) | 57424 (35420-87493) | D |
| Biannual | Symptoms | 141.8 (67.8-274.5) | 378660 (307448-451917) | 73984 (43838-111676) | ED |
|  | CXR-CAD | 208.3 (108.2-390.7) | 385659 (320256-452288) | 80983 (47912-122695) | Frontier |
|  | Symptoms + CXR-CAD | 219.7 (114.6-413) | 465089 (385809-548312) | 160413 (112005-220748) | ED |
|  | Pooled Xpert | 175.4 (87.8-332.4) | 421364 (344048-511423) | 116688 (74219-173785) | D |
| Entry + exit + annual | Symptoms | 133.4 (62.7-266) | 431555 (349463-522585) | 126879 (83816-181724) | D |
|  | CXR-CAD | 195 (99.3-373.2) | 453859 (375841-536257) | 149183 (102741-209703) | D |
|  | Symptoms + CXR-CAD | 206.1 (105.7-395.2) | 572795 (465338-694066) | 268119 (199214-356436) | D |
|  | Pooled Xpert | 164.2 (82.2-317.4) | 504948 (409779-626590) | 200272 (135848-293726) | D |
| Entry + exit + biannual | Symptoms | 180.3 (88.6-350) | 470270 (379962-573312) | 165594 (108120-238890) | D |
|  | CXR-CAD | 246.6 (128.3-464.3) | 500133 (413474-597984) | 195457 (135096-273287) | Frontier |
|  | Symptoms + CXR-CAD | 257 (135.5-482.5) | 660055 (537702-799550) | 355379 (267081-471627) | Frontier |
|  | Pooled Xpert | 215.3 (110.9-413.5) | 565428 (453874-713538) | 260752 (174496-381190) | D |
| Colombia | | | | |  |
| Base case | N/A | 0 | 233696 (185309-291875) | 0 | Frontier |
| Entry | Symptoms | 41.6 (12.9-105.2) | 253556 (203136-313984) | 19860 (12391-30622) | ED |
|  | CXR-CAD | 62.9 (20.9-156.6) | 257622 (207849-318121) | 23927 (15918-34728) | D |
|  | Symptoms + CXR-CAD | 66.8 (22.2-164.4) | 276311 (223881-336265) | 42616 (29882-59324) | D |
|  | Pooled Xpert | 52.2 (16.9-130.8) | 260030 (211149-320463) | 26334 (16820-38502) | D |
| Exit | Symptoms | 7.3 (1.8-21.6) | 241179 (191528-300764) | 7484 (4877-10969) | ED |
|  | CXR-CAD | 11.9 (3.2-33.7) | 243830 (194699-303375) | 10135 (6959-14424) | ED |
|  | Symptoms + CXR-CAD | 12.8 (3.5-35.7) | 249877 (199637-312008) | 16182 (11438-22144) | ED |
|  | Pooled Xpert | 9.5 (2.5-26.7) | 244941 (195721-303224) | 11246 (7507-15917) | D |
| Annual | Symptoms | 80.5 (26-194.5) | 254494 (205520-314281) | 20799 (11315-32395) | ED |
|  | CXR-CAD | 120.4 (42.1-288.6) | 256909 (209879-314280) | 23213 (13164-35929) | Frontier |
|  | Symptoms + CXR-CAD | 127 (44.4-300.5) | 279322 (230382-340202) | 45627 (31214-62910) | ED |
|  | Pooled Xpert | 100.8 (34.2-240.6) | 261038 (213087-319393) | 27343 (16432-39704) | D |
| Biannual | Symptoms | 124.3 (42.5-296.1) | 276515 (226881-339450) | 42819 (24600-65079) | ED |
|  | CXR-CAD | 162.8 (59.2-379.4) | 284616 (235236-344845) | 50920 (32287-74515) | Frontier |
|  | Symptoms + CXR-CAD | 168 (61.5-387.6) | 330300 (273776-396095) | 96605 (68795-130613) | D |
|  | Pooled Xpert | 145.7 (52.5-342.3) | 290181 (239029-351242) | 56486 (36091-80727) | D |
| Entry + exit + annual | Symptoms | 108.5 (36.8-259.8) | 281768 (230352-344882) | 48072 (29913-71828) | D |
|  | CXR-CAD | 148.1 (53.2-347.3) | 291655 (240461-352487) | 57959 (39010-83847) | D |
|  | Symptoms + CXR-CAD | 154.1 (55.5-357.1) | 339368 (280034-409019) | 105673 (77467-144019) | D |
|  | Pooled Xpert | 129.6 (46-305.7) | 298039 (243368-361263) | 64343 (42235-92000) | D |
| Entry + exit + biannual | Symptoms | 140.9 (49.4-325.9) | 304457 (248060-370646) | 70761 (44186-105811) | D |
|  | CXR-CAD | 175.1 (64.2-404.2) | 320799 (265685-387393) | 87104 (59913-123801) | Frontier |
|  | Symptoms + CXR-CAD | 179.5 (66.4-413) | 391939 (324151-470299) | 158243 (116039-215054) | Frontier |
|  | Pooled Xpert | 160.5 (58.1-378.4) | 328027 (268947-395029) | 94331 (62884-133815) | D |
| Peru | | | | |  |
| Base case | N/A | 0 | 1017841 (791234-1299987) | 0 | Frontier |
| Entry | Symptoms | 137.1 (36.9-329.1) | 1051581 (818771-1341385) | 33740 (15808-62365) | D |
|  | CXR-CAD | 211.2 (61-484.4) | 1055389 (824459-1344143) | 37548 (15183-70844) | D |
|  | Symptoms + CXR-CAD | 224.6 (66.1-512.5) | 1082400 (846666-1376055) | 64559 (36222-105301) | D |
|  | Pooled Xpert | 173.9 (49.5-405.2) | 1060174 (825542-1343333) | 42333 (20402-76436) | D |
| Exit | Symptoms | 19 (4.1-54) | 1028190 (800329-1312745) | 10349 (6163-16742) | ED |
|  | CXR-CAD | 31.2 (7.2-87.4) | 1032016 (804391-1316082) | 14175 (8199-23562) | ED |
|  | Symptoms + CXR-CAD | 33.6 (7.9-94.4) | 1038455 (808680-1324727) | 20614 (13120-31471) | ED |
|  | Pooled Xpert | 24.8 (5.7-68.9) | 1032982 (804583-1318178) | 15141 (9129-24301) | D |
| Annual | Symptoms | 275.1 (78.7-630.8) | 1053342 (816661-1340162) | 35500 (10089-72407) | D |
|  | CXR-CAD | 425.8 (132.8-954.1) | 1045096 (813419-1328612) | 27254 (-3512-72172) | Frontier |
|  | Symptoms + CXR-CAD | 452.9 (140.7-1016) | 1079097 (843251-1366883) | 61256 (27508-112081) | D |
|  | Pooled Xpert | 349.8 (106.1-789.4) | 1059282 (820309-1339507) | 41441 (10992-86095) | D |
| Biannual | Symptoms | 444.6 (132.1-1012.2) | 1080777 (843814-1372320) | 62936 (22277-117648) | D |
|  | CXR-CAD | 620.9 (199.8-1371.1) | 1060596 (835272-1341877) | 42755 (2133-93623) | Frontier |
|  | Symptoms + CXR-CAD | 648.6 (210.3-1433.8) | 1130588 (896735-1419920) | 112747 (62481-181052) | D |
|  | Pooled Xpert | 537.5 (167.6-1195.1) | 1086485 (851926-1366876) | 68643 (23427-128476) | D |
| Entry + exit + annual | Symptoms | 373.5 (109.4-866.6) | 1090072 (852274-1384766) | 72231 (33211-126147) | D |
|  | CXR-CAD | 535.9 (168.6-1192.4) | 1082419 (853899-1362927) | 64578 (22748-119507) | D |
|  | Symptoms + CXR-CAD | 563 (175.8-1241.3) | 1150246 (910081-1439005) | 132405 (77866-200403) | D |
|  | Pooled Xpert | 457.2 (139.1-1013.6) | 1103218 (863558-1391247) | 85377 (39630-150243) | D |
| Entry + exit + biannual | Symptoms | 511 (157.2-1168.4) | 1115344 (877428-1406670) | 97502 (44837-163716) | D |
|  | CXR-CAD | 679.1 (218.7-1494.1) | 1098123 (873835-1381391) | 80282 (29262-137914) | Frontier |
|  | Symptoms + CXR-CAD | 704.4 (230.5-1539.6) | 1203201 (966487-1496138) | 185359 (113691-272471) | Frontier |
|  | Pooled Xpert | 601.8 (188.1-1329.8) | 1127773 (893327-1411787) | 109932 (51529-182983) | D |

**Table E. Optimal strategies by prison incidence.** Ranges of prison incidence under which each strategy has the highest probability of being the optimal strategy. N/A indicates that a given strategy was not optimal at any tested incidence level.

| **Optimal strategy (intervention, algorithm)** | **Incidence range (per 100,000 person-years)** | | |
| --- | --- | --- | --- |
|  | **Brazil** | **Colombia** | **Peru** |
| Base case | ≤ 116 | ≤ 121 | N/A |
| Annual screening, CXR-CAD | 117 - 214 | 122 - 297 | ≤ 443 |
| Biannual screening, CXR-CAD | 215 - 983 | 298 - 1080 | 444 - 1108 |
| Entry + exit + biannual, CXR-CAD | ≥ 984 | ≥ 1081 | 1109 - 5336 |
| Entry + exit + biannual, symptoms & CXR-CAD | N/A | N/A | ≥ 5337 |

**Table F. Costs, effects, and cost-effectiveness of strategies on the efficiency frontier if CXR-CAD were unavailable.** Mean estimates are shown, standardized per 100,000 population. All estimates are population-wide (i.e., they include costs and effects accrued by the entire population, not just those in prison). Only strategies on the efficiency frontier are shown; all other strategies were dominated. Costs are in 2023 US dollars. DALYs, disability-adjusted life years; increm., incremental; ICER, incremental cost-effectiveness ratio.

| **Strategy (intervention, algorithm)** | **Cost (USD)** | **Effect (DALYs averted)** | **Increm. cost** | **Increm. effect** | **ICER** |
| --- | --- | --- | --- | --- | --- |
| Brazil | | | | | |
| Base case | 304676 | 0 | NA | NA | NA |
| Annual, symptoms | 340920 | 82 | 36244 | 82 | 440 |
| Biannual, symptoms | 378660 | 142 | 37739 | 59 | 634 |
| Biannual, pooled Xpert | 421364 | 175 | 42704 | 34 | 1270 |
| Entry + exit + biannual, pooled Xpert | 565428 | 215 | 144064 | 40 | 3607 |
| Colombia | | | | | |
| Base case | 233696 | 0 | NA | NA | NA |
| Annual, symptoms | 254494 | 81 | 20799 | 81 | 258 |
| Annual, pooled Xpert | 261038 | 101 | 6544 | 20 | 323 |
| Biannual, pooled Xpert | 290181 | 146 | 29143 | 45 | 649 |
| Entry + exit + biannual, pooled Xpert | 328027 | 161 | 37846 | 15 | 2551 |
| Peru | | | | | |
| Base case | 1017841 | 0 | NA | NA | NA |
| Annual, pooled Xpert | 1059282 | 350 | 41441 | 350 | 118 |
| Biannual, pooled Xpert | 1086485 | 538 | 27203 | 188 | 145 |
| Entry + exit + biannual, pooled Xpert | 1127773 | 602 | 41289 | 64 | 642 |

**Table G. Costs of and DALYs averted by combined entry, exit and biannual screening with CXR-CAD, assuming declining test sensitivity.** Test sensitivity was reduced linearly during years 0-5 of the intervention period to levels consistent with asymptomatic disease, for both the screening step with CXR-CAD and follow-up testing with Xpert Ultra. Means and 95% uncertainty intervals are shown. Costs are in 2023 US dollars.

| Country | Scenario | Additional cost (relative to base-case) | DALYs averted |
| --- | --- | --- | --- |
| Brazil | Constant sensitivity (main analysis) | 195457 (135096-273287) | 247 (128-464) |
|  | Declining sensitivity | 202353 (142037-279722) | 219 (113-414) |
| Colombia | Constant sensitivity (main analysis) | 87104 (59913-123802) | 175 (64-404) |
|  | Declining sensitivity | 88799 (61644-125400) | 165 (60-381) |
| Peru | Constant sensitivity (main analysis) | 80282 (29264-137913) | 679 (219-1494) |
|  | Declining sensitivity | 97823 (45370-160321) | 609 (194-1343) |

**Fig A. Model fit to incarceration-related data targets.** Black points and error bars represent data targets and 95% uncertainty bounds (if applicable), respectively. Dark blue lines and shaded bands represent median model fits and 95% uncertainty intervals, respectively. In Brazil, recidivism data was only available for one year (2013); the calibration target and uncertainty bounds are shown by the vertical black and dotted lines, respectively. Incarc prev, incarceration prevalence; 100k, 100,000 population age 15+.

**Fig B. Model fit to tuberculosis-related data targets.** Black points and error bars represent calibration targets and 95% uncertainty bounds (if applicable), respectively. Dark blue lines and shaded bands represent median model fits and 95% uncertainty intervals, respectively. “Combined” indicates population-wide notifications and incidence estimates. 100k, 100,000 person-years.

**Fig C. Carceral characteristics and projected tuberculosis incidence in included countries.** Model projections for carceral characteristics at baseline and tuberculosis incidence in 2035 under the base-case scenario. Incarceration rates are for the population aged 15 and older. Recidivism refers to the proportion of people entering prison who have a prior incarceration history. Country borders obtained from Natural Earth, accessed via the maps R package. Available at <https://www.naturalearthdata.com>.

**Fig D. Tuberculosis incidence over time under base-case and intervention scenarios**. Only interventions employing CXR-CAD are shown. Prison incidence is depicted in the top row; population-level incidence is in the bottom row. The shaded gray band shows the intervention period.

**Fig E. Intervention costs, broken down by component.** Follow-up testing includes costs of culture and DST for people diagnosed with TB. Passive diagnosis costs indicate costs averted by screening interventions, relative to the base-case scenario. Error bars show 95% uncertainty intervals. Costs are in 2023 US dollars (USD).

**Fig F. Proportion of true and false positives among treated individuals under each screening strategy.** Mean proportions are shown.

**Fig G. Uncertainty in costs and DALYs averted of each screening strategy.** Costs are in 2023 US dollars (USD) and are additional relative to the base case scenario. 95% uncertainty ellipses are shown for each strategy. The straight black line in each panel indicates the willingness-to-pay threshold.

**Fig H. Cost-effectiveness acceptability curves and frontier.** Curves show the proportion of all iterations in which each strategy yielded the greatest net monetary benefit across a range of willingness-to-pay thresholds. Open squares indicate the cost-effectiveness acceptability frontier, comprised of the optimal strategy at each willingness-to-pay threshold (based on the expectation of net monetary benefit across iterations). The vertical dashed line indicates each country’s cost-effectiveness threshold.

**Fig I. Cost-effectiveness plane without algorithms using CXR-CAD.** Strategies on the efficient frontier are highlighted. Costs (2023 USD) are additional relative to the base case scenario. DALYs, disability-adjusted life years.

**References**

1. Martinez L, Warren JL, Harries AD, Croda J, Espinal MA, Olarte RAL, et al. Global, regional, and national estimates of tuberculosis incidence and case detection among incarcerated individuals from 2000 to 2019: a systematic analysis. The Lancet Public Health. 2023;8(7):e511-e9. doi: 10.1016/S2468-2667(23)00097-X.

2. Liu YE, Mabene Y, Camelo S, Rueda ZV, Pelissari DM, Dockhorn Costa Johansen F, et al. Mass incarceration as a driver of the tuberculosis epidemic in Latin America and projected effects of policy alternatives: a mathematical modelling study. The Lancet Public Health. 2024;9(11):e841-e51. doi: 10.1016/S2468-2667(24)00192-0.

3. Calderwood CJ, Wilson JP, Fielding KL, Harris RC, Karat AS, Mansukhani R, et al. Dynamics of sputum conversion during effective tuberculosis treatment: A systematic review and meta-analysis. PLOS Medicine. 2021;18(4):e1003566. doi: 10.1371/journal.pmed.1003566.

4. Mabene Y, Bampi J, Lemos EF, de Oliveira R, Gonçalves C, Delgado Alves MdL, et al. Tuberculosis treatment outcomes after transfer or release from incarceration: A retrospective cohort study from Brazil. medRxiv. 2025:2025.04.19.25325982. doi: 10.1101/2025.04.19.25325982.

5. Faust L, Caceres-Cardenas G, Martinez L, Huddart S, Vidal JR, Corilloclla-Torres R, et al. Tuberculosis case notifications and outcomes in Peruvian prisons prior to and during the COVID-19 pandemic: a national-level interrupted time series analysis. Lancet Reg Health Am. 2024;33:100723. Epub 2024/05/27. doi: 10.1016/j.lana.2024.100723. PubMed PMID: 38800646; PubMed Central PMCID: PMCPMC11117008.

6. Emani S, Alves K, Alves LC, da Silva DA, Oliveira PB, Castro MC, et al. Quantifying gaps in the tuberculosis care cascade in Brazil: A mathematical model study using national program data. PLOS Medicine. 2024;21(3):e1004361. doi: 10.1371/journal.pmed.1004361.

7. Ministerio de Salud DGdM, Insumos y Drogas,. Estimación del umbral costo - efectividad para las evaluaciones económicas en salud. Informe técnico. Perú: 2022.

8. Ministério da Saúde. O uso de limiares de custo-efetividade nas decisões em saúde: recomendações da Comissão Nacional de Incorporação de Tecnologias no SUS. In: Secretaria de Ciência T, Inovação e Insumos Estratégicos em Saúde; Departamento de Gestão e Incorporação de Tecnologias em Saúde, editor. Brasilia/DF, Brazil2022.

9. Espinosa O, Rodríguez-Lesmes P, Orozco L, Ávila D, Enríquez H, Romano G, et al. Estimating cost-effectiveness thresholds under a managed healthcare system: experiences from Colombia. Health Policy and Planning. 2022;37(3):359-68. doi: 10.1093/heapol/czab146.

10. Ripley B, Venables W. nnet: Feed-Forward Neural Networks and Multinomial Log-Linear Models. 2025.

11. Schwalb A, Horton KC, Emery JC, Harker MJ, Goscé L, Veeken LD, et al. Potential impact, costs, and benefits of population-wide screening interventions for tuberculosis in Viet Nam: A mathematical modelling study. PLOS Glob Public Health. 2025;5(9):e0005050. Epub 20250910. doi: 10.1371/journal.pgph.0005050. PubMed PMID: 40929179; PubMed Central PMCID: PMCPMC12422431.

12. Mabud TS, de Lourdes Delgado Alves M, Ko AI, Basu S, Walter KS, Cohen T, et al. Evaluating strategies for control of tuberculosis in prisons and prevention of spillover into communities: An observational and modeling study from Brazil. PLOS Medicine. 2019;16(1):e1002737. doi: 10.1371/journal.pmed.1002737.

13. Liu YE, Lemos EF, Gonçalves CCM, de Oliveira RD, Santos AdS, do Prado Morais AO, et al. All-cause and cause-specific mortality during and following incarceration in Brazil: A retrospective cohort study. PLOS Medicine. 2021;18(9):e1003789. doi: 10.1371/journal.pmed.1003789.

14. Menzies NA, Wolf E, Connors D, Bellerose M, Sbarra AN, Cohen T, et al. Progression from latent infection to active disease in dynamic tuberculosis transmission models: a systematic review of the validity of modelling assumptions. Lancet Infect Dis. 2018;18(8):e228-e38. Epub 2018/04/15. doi: 10.1016/s1473-3099(18)30134-8. PubMed PMID: 29653698; PubMed Central PMCID: PMCPMC6070419.

15. Andrews JR, Noubary F, Walensky RP, Cerda R, Losina E, Horsburgh CR. Risk of progression to active tuberculosis following reinfection with Mycobacterium tuberculosis. Clin Infect Dis. 2012;54(6):784-91. Epub 2012/01/24. doi: 10.1093/cid/cir951. PubMed PMID: 22267721; PubMed Central PMCID: PMCPMC3284215.

16. Blower SM, McLean AR, Porco TC, Small PM, Hopewell PC, Sanchez MA, et al. The intrinsic transmission dynamics of tuberculosis epidemics. Nature Medicine. 1995;1(8):815-21. doi: 10.1038/nm0895-815.

17. Ragonnet R, Flegg JA, Brilleman SL, Tiemersma EW, Melsew YA, McBryde ES, et al. Revisiting the Natural History of Pulmonary Tuberculosis: A Bayesian Estimation of Natural Recovery and Mortality Rates. Clin Infect Dis. 2021;73(1):e88-e96. Epub 2020/08/09. doi: 10.1093/cid/ciaa602. PubMed PMID: 32766718.

18. World Population Prospects 2022. In: Nations U, editor. 27 ed2022.

19. WHO. Global tuberculosis report 2024. 2024.

20. Casos de Tuberculose - Desde 2001 (SINAN) [Internet]. 2024 [cited October 20, 2024]. Available from: <https://datasus.saude.gov.br/acesso-a-informacao/casos-de-tuberculose-desde-2001-sinan/>.

21. Santos AdS, de Oliveira RD, Lemos EF, Lima F, Cohen T, Cords O, et al. Yield, Efficiency, and Costs of Mass Screening Algorithms for Tuberculosis in Brazilian Prisons. Clinical Infectious Diseases. 2021;72(5):771-7. doi: 10.1093/cid/ciaa135.

22. Pivetta de Araujo RC, Martinez L, da Silva Santos A, Ferreira Lemos E, Dias de Oliveira R, Croda M, et al. Serial Mass Screening for Tuberculosis Among Incarcerated Persons in Brazil. Clin Infect Dis. 2024;78(6):1669-76. Epub 2024/02/07. doi: 10.1093/cid/ciae055. PubMed PMID: 38324908; PubMed Central PMCID: PMCPMC11175667.

23. Guerra J, Mogollón D, González D, Sanchez R, Rueda ZV, Parra-López CA, et al. Active and latent tuberculosis among inmates in La Esperanza prison in Guaduas, Colombia. PLOS ONE. 2019;14(1):e0209895. doi: 10.1371/journal.pone.0209895.

24. MINSA - DPCTB :: Portal de Información [Internet]. 2024 [cited October 20, 2024]. Available from: <http://www.tuberculosis.minsa.gob.pe/dashboardDPCTB/dashboard.aspx>.

25. Pinto M, Steffen RE, Cobelens F, van den Hof S, Entringer A, Trajman A. Cost-effectiveness of the Xpert® MTB/RIF assay for tuberculosis diagnosis in Brazil. Int J Tuberc Lung Dis. 2016;20(5):611-8. Epub 2016/04/17. doi: 10.5588/ijtld.15.0455. PubMed PMID: 27084814.

26. Shah L, Rojas M, Mori O, Zamudio C, Kaufman JS, Otero L, et al. Cost-effectiveness of active case-finding of household contacts of pulmonary tuberculosis patients in a low HIV, tuberculosis-endemic urban area of Lima, Peru. Epidemiology and Infection. 2017;145(6):1107-17. Epub 2017/02/06. doi: 10.1017/S0950268816003186.

27. Vesga JF, Mohamed MS, Shandal M, Jabbour E, Lomtadze N, Kubjane M, et al. The Return on Investment of Scaling Tuberculosis Screening and Preventive Treatment: A Modelling Study in Brazil, Georgia, Kenya, and South Africa. medRxiv. 2024:2024.03.12.24303930. doi: 10.1101/2024.03.12.24303930.

28. Resch SC, Salomon JA, Murray M, Weinstein MC. Cost-Effectiveness of Treating Multidrug-Resistant Tuberculosis. PLOS Medicine. 2006;3(7):e241. doi: 10.1371/journal.pmed.0030241.
