# Supplementary material for: Comparative impacts and cost-effectiveness of tuberculosis systematic screening strategies in prisons in Brazil, Colombia, and Peru: A mathematical modeling study
Source: PLoS Med. 2026 Jun 4;23(6):e1004739. doi: 10.1371/journal.pmed.1004739 (PMC13258141; doi:10.1371/journal.pmed.1004739)
Supplement: Attachment — Submitted filename: ResponseToReviewersEditors.docx [file pmed.1004739.s003.docx]

**Point-by-point responses to reviewers and editorial points**

Comments from the academic editor:

Reporting the composition of costs for different strategies would help contextualize these results from a planning perspective. A stacked bar chart showing the breakdown of costs on the efficiency frontier would be particularly useful- specifically, the proportion of costs attributable to screening (by screen), follow-up diagnostics, correct treatment, and 'unnecessary treatment' (only for those false positives on both screening and diagnostic tests). This breakdown would clarify which cost components increase or decrease across strategies, providing practical insights for resource allocation and implementation planning.

**RESPONSE:** We appreciate this astute suggestion. We have added a supplemental figure showing the breakdown of intervention costs for strategies on the efficiency frontier. We chose to include dodged rather than stacked bars to make it easier to compare each cost component across strategies.

**Figure S5. Intervention costs, broken down by component.** Follow-up testing includes costs of culture and DST for people diagnosed with TB. Passive diagnosis costs indicate costs averted by screening interventions, relative to the base-case scenario. Error bars show 95% uncertainty intervals. Costs are in 2023 US dollars (USD).

We refer to this in the text as follows (line 427): “Examining the breakdown of intervention costs revealed that strategy’s 4 additional costs were driven by substantially higher costs of follow-up testing and unnecessary treatment, due to the algorithm’s lower specificity (**Figure S5**).”

-----------------------------------------------------------

Comments from the reviewers:

Reviewer #1: This modelling study addresses a fundamentally important research question in tuberculosis control, is very well executed from a technical perspective, and is very nicely written. The results are likely to have immediate and important implications for both public and individual health. Beyond the tuberculosis community, the results will generate broad interest among the readership of PLOS Medicine as they draw attention to the importance of health among incarcerated populations, a vulnerable and neglected population, and show how the health of incarcerated populations matters to the health of the general population. The researchers clearly present very thorough projections based on plausible active case finding interventions. They account for uncertainty and different scenarios (e.g. different incidences, different screening algorithms) very completely. I think the discussion articulates well the key issues around incidence rebounding after the intervention period, the need to implement immediate screening with available tools, and the importance of structural reforms to reduce incarceration and crowding in prisons. I believe the key limitations and weaknesses are acknowledged, and future research priorities are appropriately articulated, including the need for empirical studies.

Congratulations on this important work and thank you for your attention to this matter.

**RESPONSE:** We are grateful to the reviewer for their thoughtful review of the study and for their positive feedback.

Reviewer #2: This modeling manuscript reports a highly relevant set of analyses predicting the impact and cost-effectiveness of potential screening interventions for TB in the prisons of Brazil, Colombia and Peru. It is clearly written; the analyses have been rigorously executed and provide important and actionable results. The consideration of population-wide epidemiologic impact is of major interest. The authors provide a thoughtful discussion of key points/implications, strengths and limitations of their work. I have relatively few suggestions for improvement.

**RESPONSE:** We thank the reviewer for their positive feedback and suggestions to improve the manuscript.

Major comments:

1. This work builds on earlier modeling, published by Liu et al (Lancet Public Health, 2024). While calibration is well described in the present manuscript, key elements of the model and some important underlying assumptions/parameters are missing. For example, I could not find the TB case fatality rate listed anywhere; this is obviously essential to DALY estimates and needs to be stated clearly in the main text and/or the main tables. My own preference would also be to show the key assumptions related to DALY weights and life expectancy in table form in the main paper, so that readers can understand the main elements of the DALY calculations without having to refer to the appendix. More generally, I think the structure and key flows/rates for the dynamic model should be shown in the appendix, even if this repeats some material in the 2024 paper.

**RESPONSE:** We thank the reviewer for highlighting the importance of clearly presenting these assumptions. We have now added a Table in the main manuscript (Table 2) with key parameters affecting DALY estimates, including the TB mortality rate, disability weights, and life expectancy:

**Table 2. Parameters for DALY estimation.** The TB mortality rate is an annualized rate assuming a prevalence of smear-positive TB ranging between 10-50%. Triangle distributions are specified as Triangle(mode, minimum, maximum).

| **Parameter** | **Country** | **Distribution** | **Source** |
| --- | --- | --- | --- |
| Disability weight for infectious active TB disease | All | Triangle(0.33, 0.25, 0.4) | ^35^ |
| Post-TB disability weight | All | Triangle (0.038, 0.02, 0.05) | ^14^ |
| TB mortality rate | All | Uniform(0.061, 0.21) | ^36^ |
| Remaining life expectancy at average age of developing TB among population aged 15 and older (years) | Brazil | Triangle (44, 39, 48) | ^37^ |
|  | Colombia | Triangle (42, 37, 47) | ^37^ |
|  | Peru | Triangle (47, 42, 52) | ^37^ |

We have moved the description of key assumptions related to DALY weights and life expectancy to the Methods section of the main text (line 222):

“DALY-related parameters are shown in **Table 2**. To project lifetime years of life lost (YLLs) for people who die from tuberculosis, we calculated the average age of people developing tuberculosis among the population 15 and older^1^ (37 in Brazil, 40 in Colombia, and 37 in Peru) and retrieved remaining life expectancy at that age (**Table 2**). We assumed similar life expectancy across population strata. To project years lived with disability (YLDs) due to post-tuberculosis sequelae, we applied a post-tuberculosis disability weight over the remaining life expectancy of individuals who recover from tuberculosis (**Table 2**). At the end of the analytic horizon, individuals with tuberculosis were assumed to either recover or die immediately from tuberculosis with probabilities based on competing risks. We did not include elevated all-cause mortality for tuberculosis survivors given uncertainty around the true causal risk ratio.”

Furthermore, we have added a model diagram to the main text (**Figure 1**) and included a table in the Appendix (**Table S1**) with parameter values and distributions for key model transitions:

**Figure 1. Compartmental transmission model schematic.** The model includes five tuberculosis disease states: susceptible (S), early latent infection (E), late latent infection (L), infectious active disease (I), recovered (R). These disease states are replicated across four incarceration-related population strata, denoted with subscripts: prison (p), recent history of incarceration (r), distant history of incarceration (d), never incarcerated (n). Solid black arrows represent tuberculosis natural history transitions (e.g. infection, progression to disease, recovery, relapse). Dashed black arrows represent transitions between population strata (e.g. incarceration, release, re-incarceration). Colored arrows indicate transitions resulting from systematic screening interventions, which move individuals from infectious active disease (I) to recovered (R). Entry screening (including screening upon inter-prison transfer) is shown in gold; exit screening is shown in purple, and prison-wide screening (annual or biannual) is shown in orange. For entry and exit screening, recovery from disease occurs concurrently with incarceration-related transitions.

**Table S1.** Key compartmental model parameters. Parameters were calibrated, fixed, or sampled. For calibrated parameters, medians and 95% intervals are shown; for sampled parameters, sampling distributions are shown. For time-varying parameters (e.g. baseline mortality rate), values for 2026 are shown. Subscripts denote the incarceration-related population stratum for which a parameter applies; parameters without subscripts are assumed to be equivalent across strata. All rates are annualized. For further details, see Liu et al. *Lancet Public Health* 2024^9^.

2. The analysis includes identification of the "optimal" screening strategy at varying prison TB incidence (Figure 3). This is very useful. However, how the "optimal" strategy is defined should be clearly spelled out for readers in the methods section—it is not really stated until lines 296-298 in the results section.

**RESPONSE:** We appreciate the reviewer's assessment of the utility of this analysis, and agree that how the “optimal” strategy is defined should be clearly spelled out in the Methods. We have now added this sentence to the Methods section (line 250): “For each country, the optimal strategy was defined as the strategy that provided the greatest health benefit (DALYs averted) while remaining within the cost-effectiveness threshold.”

3. While there is extensive attention to uncertainty with respect to epidemiologic impact of the various strategies, there is much less exploration of uncertainty with respect to costs and cost-effectiveness. It is reasonable to define the efficiency frontier using the mean values for incremental costs and DALYs as was done (e.g. figure 2). However, the cost-effectiveness planes shown in that figure do not display any ranges around the point estimates, and there are some alternative strategies with point estimates that fall very close to the frontier. Similarly, for the ICER estimates comparing strategies along the efficiency frontier, it would be appropriate to show probabilistic sensitivity analyses—in the form of cost-effectiveness planes graphing the incremental cost and effectiveness for each set of parameters and inputs, and/or cost-effectiveness acceptability curves. These could be added to the appendix.

**RESPONSE:** We are grateful to the reviewer for raising this important point. We have now added supplementary figures depicting the joint uncertainty in costs and DALYs averted for each strategy on the cost-effectiveness planes, as well as cost-effectiveness acceptability curves. We describe these additional analyses in the Methods as follows (line 261):

“For the cost-effectiveness analysis, we assessed joint uncertainty in additional costs (relative to the base case scenario) and DALYs averted for each strategy. We also conducted a probabilistic sensitivity analysis and generated cost-effectiveness acceptability curves, depicting the probability each strategy yields the greatest net monetary benefit at varying willingness-to-pay thresholds. We additionally plot the cost-effectiveness acceptability frontier, indicating the optimal strategy across willingness-to-pay thresholds, which may differ from the strategy with the highest probability of being cost-effective[21].”

We describe the results as follows (line 452):

*“Sensitivity analysis: decision uncertainty*

Probabilistic sensitivity analyses showed considerable joint uncertainty and overlap in costs and DALYs averted across strategies (**Figure S7**). Nonetheless, cost-effectiveness acceptability curves indicated strong preferences for the combination of entry, exit, and biannual screening at country-specific willingness-to-pay thresholds (**Figure S8**). Screening with CXR-CAD alone was most cost-effective in 95% of iterations in Brazil and 81% in Colombia, while in Peru the preferred strategy alternated between using CXR-CAD alone (36%) and using both symptoms and CXR-CAD (64%). Biannual screening with CXR-CAD became the preferred strategy at lower willingness-to-pay thresholds.”

**Figure S7. Uncertainty in costs and DALYs averted of each screening strategy.** Costs are in 2023 US dollars (USD) and are additional relative to the base case scenario. 95% uncertainty ellipses are shown for each strategy.

**Figure S8. Cost-effectiveness acceptability curves and frontier.** Curves show the proportion of all iterations in which each strategy yielded the greatest net monetary benefit across a range of willingness-to-pay thresholds. Open squares indicate the cost-effectiveness acceptability frontier, comprised of the optimal strategy at each willingness-to-pay threshold (based on the expectation of net monetary benefit across iterations). The vertical dashed line indicates each country’s cost-effectiveness threshold.

Minor comment:

In figure 2, some graphs have axes with costs in scientific notation i.e. in exponential form, while some have axes with costs in whole numbers. I'd suggest you convert all to the latter.

**RESPONSE:** We have now replaced scientific notation with whole numbers in all graphs as suggested.

Reviewer #3: Dear editor,

This is an important and thorough modelling study around the potential and shape of systematic screening in prisons in Latin America. The work is overall well described, and should help influence important policy decisions in the region. While this is certainly worth publishing and has a good place in PMED, there are a few areas I think the paper could be improved by revision or more explanation, which I outline below.

**RESPONSE:** We thank the reviewer for their positive comments and for their suggestions to improve the manuscript.

Abstract:

Slight disconnect between results and conclusions - results do not state that any strategy (including just symptom followed by Xpert) was CE, but conclusion suggest just use any technique. I think the authors mean use pooled Xpert at minimum, then move to CXR-CAD, but it is not clear, and should be. Suggest adding clarification in findings or conclusion in abstract. I think l366-370 cover the relevant results, but the abstract should be self-contained.

**RESPONSE:** We appreciate the reviewer pointing out this inconsistency. We have edited the Conclusions of the Abstract to the following:

“These modeling results support immediate national-level adoption of prison-wide tuberculosis screening twice-yearly and at entry and exit, using CXR-CAD or pooled Xpert.”

Methods

Strongly urge the authors to include a model diagramme in the main text to support the reader's understanding, including the core model and relevant subdivisions.

**RESPONSE:** We agree and have added a model diagram to the main text illustrating the model structure (disease states and population strata), transitions, and intervention pathways.

**Figure 1. Compartmental transmission model schematic.** The model includes five tuberculosis disease states: susceptible (S), early latent infection (E), late latent infection (L), infectious active disease (I), recovered (R). These disease states are replicated across four incarceration-related population strata, denoted with subscripts: prison (p), recent history of incarceration (r), distant history of incarceration (d), never incarcerated (n). Solid black arrows represent tuberculosis natural history transitions (e.g. infection, progression to disease, recovery, relapse). Dashed black arrows represent transitions between population strata (e.g. incarceration, release, re-incarceration). Colored arrows indicate transitions resulting from systematic screening interventions, which move individuals from infectious active disease (I) to recovered (R). Entry screening (including screening upon inter-prison transfer) is shown in gold; exit screening is shown in purple, and prison-wide screening (annual or biannual) is shown in orange. For entry and exit screening, recovery from disease occurs concurrently with incarceration-related transitions.

Regarding model structure, the authors assume a single TB state, with constant test performance in each round of screening (Table 1). That single state summarises a lot of complexity and assumptions (e.g. what proportion is symptomatic, has CXR changes, is bacteriologically positive?) and also assumes that the distribution of severity (in terms of CXR change, symptoms or Xpert) among those 'with TB' remains the same each round over 10 years of intensive systematic screening and substantially dropping burden (figure S4), In reality, I would expect that make up to shift toward less severe disease, and worry that this simplification of TB natural history is a major limitation in the context of examining the impact of repeated screening with different tools, likely overestimating the number of diagnoses and the overall impact. Perhaps not much of an issue in the entry screening, it does affect the other target groups. While it is not reasonable to change the model structure at this point, this issue does need addressing by the authors. I would suggest two approaches: 1) explore a shift in the test positivity values over time to reflect a change in the make up of the screened population 2) discuss this choice and limitation explicitly in the methods, describing the assumed make up of the 'disease' state in terms of bacteriology, symptoms and radiology, and discussion (where it is not currently mentioned in the limitations).

**RESPONSE:** We appreciate this thoughtful suggestion from the reviewer and have added a sensitivity analysis to assess how a shift toward less severe TB over time would affect our results. We describe this sensitivity analysis in the Methods as follows (line 274):

“Third, repeated intensive screening may lead to earlier detection and treatment, such that the clinical and bacteriological profile of TB in the screened population may shift over time toward less severe disease (i.e., higher proportions of asymptomatic and/or bacteriologically negative TB). To assess how this shift might affect projected health impacts and cost-effectiveness, we gradually decreased test sensitivity for both screening and follow-up testing over the intervention period.”

We further elaborate in the Appendix:

“For the third sensitivity analysis, we focused on one intensive screening strategy: the combination of entry, exit, and biannual screening with CXR-CAD. To represent the shift toward less severe—and less detectable—TB disease over time, we allowed test sensitivity to decline linearly over the first five years of the intervention period, eventually reaching sensitivity values consistent with asymptomatic or subclinical TB. By year five, sensitivity of CXR-CAD screening declined to Triangle(62.6%, 71.2%, 67.7%), and sensitivity of Xpert Ultra follow-up testing declined to Triangle(67.6%, 85.6%, 77.5%)^9^. Quantile rank was preserved between sampled start and end values. We assumed that sensitivity decreased for all screening points (entry, exit, and periodic screening).”

We describe the Results as follows (line 480):
“*Sensitivity analysis: declining test sensitivity*

Assuming declining test positivity among individuals with tuberculosis due to a shift toward less severe disease, the combination of entry, exit, and biannual screening with CXR-CAD would avert 89% of DALYs in Brazil, 94% of DALYs in Colombia, and 90% of DALYs in Peru relative to the main analysis (**Table S7**). Additional costs were 4% higher in Brazil, 2% higher in Colombia, and 22% higher in Peru, driven by smaller offsets from averted passive diagnosis costs.”

**Table S7. Costs of and DALYs averted by combined entry, exit and biannual screening with CXR-CAD, assuming declining test sensitivity.** Test sensitivity was reduced linearly during years 0-5 of the intervention period to levels consistent with asymptomatic disease, for both the screening step with CXR-CAD and follow-up testing with Xpert Ultra. Means and 95% uncertainty intervals are shown. Costs are in 2023 US dollars.

| **Country** | **Scenario** | **Additional cost (relative to base-case)** | **DALYs averted** |
| --- | --- | --- | --- |
| **Brazil** | Constant sensitivity (main analysis) | 195457 (135096-273287) | 247 (128-464) |
|  | Declining sensitivity | 202353 (142037-279722) | 219 (113-414) |
| **Colombia** | Constant sensitivity (main analysis) | 87104 (59913-123802) | 175 (64-404) |
|  | Declining sensitivity | 88799 (61644-125400) | 165 (60-381) |
| **Peru** | Constant sensitivity (main analysis) | 80282 (29264-137913) | 679 (219-1494) |
|  | Declining sensitivity | 97823 (45370-160321) | 609 (194-1343) |

We also make explicit our modeling choice in the Methods (line 155):

“We simplify the documented spectrum of TB disease states^14^, assuming a single disease state (**I**) representing infectious, bacteriologically positive tuberculosis, of which 51% (range 30-70%) is assumed to be symptomatic (**Table 1**).”

And we discuss limitations in the Discussion (line 554):

“We did not model the spectrum of tuberculosis disease states, which may be particularly relevant under repeated screening if the distribution of TB disease shifts towards less detectable forms over time. In a sensitivity analysis assuming declining test positivity among those with tuberculosis, intensive screening averted slightly fewer DALYs at similar or moderately higher cost. This suggests that our simplified representation of TB disease states may modestly underestimate ICERs but likely does not change conclusions regarding the cost-effectiveness of intensive screening with CXR-CAD. In extremely high incidence prisons where our main results suggest that screening with both symptoms and CXR-CAD would be justified, a shift toward predominantly asymptomatic disease would weaken this justification.”

For Table 1 - I have not read through all the references, but it would be good if the authors clarified what the reference standard was in the studies that provided sens and spec. In particular, whether the definition of 'disease' aligns with that used in the model. E.g. was the reference standard culture positive, Xpert positive, did it include clinical assessment in absence of bacteriological test? If they align, then it is helpful to be clear. This is important, as the key results and dominant strategy is almost entirely driven by the assumed high sensitivity (and specificity) of CXR/CAD as compared to other tools (table 1).

**RESPONSE:** We appreciate the opportunity to clarify. We have added the following to the Table legend (line 609): “Sensitivity and specificity estimates were derived from studies that used bacteriologically confirmed tuberculosis as the reference standard, consistent with the model’s representation of tuberculosis disease.”

L190: is there any consideration of diagnosis of bacteriologically negative individual, e.g. based on CXR abnormalities +/- symptoms. These numbers reach ~50% of all treatment initiations in other screening efforts (e.g. doi 10.1136/bmjgh-2025-019133).

**RESPONSE:** We did not explicitly model this; we now describe this in the Limitations (line 563):

“Relatedly, we did not model bacteriologically negative tuberculosis, which accounts for approximately 10% of diagnoses in this setting[7]; future work might explore how inclusion of empirical treatment in prison screening affects impacts and cost-effectiveness.”

Results

Figure 3 is interesting. Squinting a little it seems the figures are mostly the same, just with more spread out X-axes. It is interesting that the purple line starts to dominate in the very high prison incidences, nice to comment on what model behaviour is driving that.

**RESPONSE:** We have now added the following commentary to the Discussion (line 515):
“In the model, high prison incidence reflects a combination of high within-prison transmission, rapid disease progression, and limited passive diagnosis. Our results suggest that under extreme combinations of these conditions, a slight gain in screening sensitivity justifies the increased costs.”

L309-311 - this annual vs bi-annual is an interesting observation, and less dependent (I think) on the assumptions around test performance. Good to reflect on a bit more in discussion, where it is merely repeated as a finding, but not explained through either the model setup, or how this reflects reality.

**RESPONSE:** We appreciate the reviewer's interest in this finding and now comment on it more in the Discussion (line 532):
“Entry or exit screening alone had modest impact compared to periodic prison-wide screening, and biannual screening dominated the WHO-recommended combination of entry, exit, and annual screening. These results can be attributed to the region's underlying epidemiology, reflected in the calibrated model, where tuberculosis prevalence and transmission intensity are substantially higher in prisons than in communities^12,25^. As a result, prison-wide screening generates greater yield per person screened than adding entry screening, and results in greater downstream transmission effects than exit screening. In settings with less disparity between prison and community burden and transmission rates, the impacts and cost-effectiveness of entry and exit screening may be relatively greater than observed here.”

There appear no results reported around false positive diagnoses, and how the FP/TP ratio changes between different algorithms, or how they are counted in DALY and costs. Given that they are tracked (l174-176), these results should be reported I think.

**RESPONSE:** We agree with the reviewer and have added the following clarifications to the Methods (line 231; line 242):
“Treatment was assumed to have no disability weight for true and false positives…Intervention costs included treatment costs for false positives detected through systematic screening. False positives under passive diagnosis were not accounted for.”

We have included an analysis of the proportion of true and false positives, described in Results (line 449):
“False positives accounted for a substantial proportion of individuals diagnosed by all algorithms except Xpert on pooled sputum; this proportion generally increased with screening intensity (**Figure S6**).”

**Figure S6. Proportion of true and false positives among treated individuals under each screening strategy.** Mean proportions are shown.

Discussion:

Discuss the empirical evidence for this impact. I am aware there are studies ongoing, but good to discuss to what extent these modelled findings can or will be compared against empirical data.

**RESPONSE:** We thank the reviewer for this suggestion. We have added the following to the Discussion (line 581): “Empirical studies evaluating repeated rounds of systematic screening are ongoing in Brazil; future work should compare modeled findings against empirical evidence and evaluate feasible strategies for operationalization under programmatic conditions.”

Minor:

Language issue - use of the word 'case'-finding in title and throughout - WHO and StopTB suggest alternative terms, e.g. systematic screening (as used in l108), throughout.

**RESPONSE:** We have revised the manuscript to replace “active case finding” with “systematic screening” where appropriate, in line with WHO and Stop TB Partnership terminology.

Any attachments provided with reviews can be seen via the following link: ********

--------------------------------------------------------- ---

General editorial requests:

(Note: not all will apply to your paper, but please check each item carefully)

* We ask every co-author listed on the manuscript to fill in a contributing author statement, making sure to declare all competing interests. If any of the co-authors have not filled in the statement, we will remind them to do so when the paper is revised. If all statements are not completed in a timely fashion this could hold up the re-review process. If new competing interests are declared later in the revision process, this may also hold up the submission. Should there be a problem getting one of your co-authors to fill in a statement we will be in contact. Please do not add or remove authors without first discussing this with the handling editor. You can see our competing interests policy here: <http://journals.plos.org/plosmedicine/s/competing-interests>.

**RESPONSE:** We will assist with securing contributing author statements from all co-authors as needed.

* Please upload any figures associated with your paper as individual TIF or EPS files with 300dpi resolution at resubmission; please read our figure guidelines for more information on our requirements: http://journals.plos.org/plosmedicine/s/figures. While revising your submission, we strongly recommend that you use PLOS's NAAS tool (https://ngplosjournals.pagemajik.ai/artanalysis) to test your figure files. NAAS can convert your figure files to the TIFF file type and meet basic requirements (such as print size, resolution), or provide you with a report on issues that do not meet our requirements and that NAAS cannot fix.

After uploading your figures to PLOS's NAAS tool - https://ngplosjournals.pagemajik.ai/artanalysis, NAAS will process the files provided and display the results in the "Uploaded Files" section of the page as the processing is complete.

If the uploaded figures meet our requirements (or NAAS is able to fix the files to meet our requirements), the figure will be marked as "fixed" above. If NAAS is unable to fix the files, a red "failed" label will appear above.

When NAAS has confirmed that the figure files meet our requirements, please download the file via the download option, and include these NAAS processed figure files when submitting your revised manuscript.

**RESPONSE:** We have used the NAAS tool to regenerate our figures.

* Please ensure that the paper adheres to the PLOS Data Availability Policy (see http://journals.plos.org/plosmedicine/s/data-availability), which requires that all data underlying the study's findings be provided in a repository or as Supporting Information. For data residing with a third party, authors are required to provide instructions with contact information (web or email address) for obtaining the data. Please note that a study author cannot be the contact person for the data. PLOS journals do not allow statements supported by "data not shown" or "unpublished results." For such statements, authors must provide supporting data or cite public sources that include it.

**RESPONSE:** We have reviewed the policy and will provide all data underlying the study's findings in a public repository upon manuscript acceptance.

* We expect all researchers with submissions to PLOS in which author-generated code underpins the findings in the manuscript to make all author-generated code available without restrictions upon publication of the work. In cases where code is central to the manuscript, we may require the code to be made available as a condition of publication. Authors are responsible for ensuring that the code is reusable and well documented. Please make any custom code available, either as part of your data deposition or as a supplementary file. Please add a sentence to your data availability statement regarding any code used in the study, e.g. "The code used in the analysis is available from Github [URL] and archived in Zenodo [DOI link]" Please review our guidelines at https://journals.plos.org/plosmedicine/s/materials-software-and-code-sharing and ensure that your code is shared in a way that follows best practice and facilitates reproducibility and reuse. Because Github depositions can be readily changed or deleted, we encourage you to make a permanent DOI'd copy (e.g. in Zenodo) and provide the URL.

**RESPONSE:** We will deposit all code in a public repository upon manuscript acceptance.

* Please ensure that the study is reported according to the appropriate guideline and include the completed checklist as Supporting Information. When completing the checklist, please use section and paragraph numbers, rather than page numbers. Please add the following statement, or similar, to the Methods: "This study is reported as per [XXXX] guideline (S1 Checklist)."

**RESPONSE:** We report the study using the appropriate guideline for modeling and health economic studies (see below).

FORMATTING - GENERAL

* Abstract: Please structure your abstract using the PLOS Medicine headings (Background, Methods and Findings, Conclusions). Please combine the Methods and Findings sections into one section.

**RESPONSE:** The abstract is structured as requested.

* At this stage, we ask that you include a short, non-technical Author Summary of your research to make findings accessible to a wide audience that includes both scientists and non-scientists. The Author Summary should immediately follow the Abstract in your revised manuscript. This text is subject to editorial change and should be distinct from the scientific abstract. Ideally each sub-heading should contain 2-3 single sentence, concise bullet points containing the most salient points from your study. In the final bullet point of 'What Do These Findings Mean?', please include the main limitations of the study in non-technical language. Please see our author guidelines for more information: <https://journals.plos.org/plosmedicine/s/revising-your-manuscript#loc-author-summary>.

**RESPONSE:** An Author Summary is included.

* Please express the main results with 95% CIs as well as p values. When reporting p values please report as p<0.001 and where higher as the exact p value p=0.002, for example. Throughout, suggest reporting statistical information as follows to improve clarity for the reader "22% (95% CI [13%,28%]; p</=)". Please be sure to define all numerical values at first use.

**RESPONSE:** We report 95% UIs (uncertainty intervals) as requested.

* When reporting ranges, please use a comma instead of a hyphen to separate values.

**RESPONSE:** We have changed hyphens to commas in numerical ranges.

* Please include page numbers and line numbers in the manuscript file. Use continuous line numbers (do not restart the numbering on each page).

**RESPONSE:** The manuscript file includes continuous page and line numbers.

* Please cite the reference numbers in square brackets. Citations should precede punctuation.

**RESPONSE:** References have been reformatted as requested.

FIGURES AND TABLES

* Please provide titles and legends for all figures and tables (including those in Supporting Information files).

**RESPONSE:** We have now included titles and legends for all figures and tables, including those in Supporting Information files.

* Please define all abbreviations used in each figure/table (including those in Supporting Information files).

**RESPONSE:** We define all abbreviations used in each figure/table.

* Please consider avoiding the use of red and green in order to make your figure more accessible to those with color blindness.

**RESPONSE:** Our figures limit use of red and green in combination.

SUPPLEMENTARY MATERIAL

* Please note that supplementary material will be posted as supplied by the authors. Therefore, please amend it according to the relevant comments outlined here.

**RESPONSE:** We have amended it according to the following comments.

* Please cite your Supporting Information as outlined here: <https://journals.plos.org/plosmedicine/s/supporting-information>

**RESPONSE:** We cite our Supporting Information files as requested.

REFERENCES

* PLOS uses the numbered citation (citation-sequence) method and first six authors, et al.

**RESPONSE:** The references are formatted as requested.

* Please ensure that journal name abbreviations match those found in the National Center for Biotechnology Information (NCBI) databases (http://www.ncbi.nlm.nih.gov/nlmcatalog/journals), and are appropriately formatted and capitalised.

**RESPONSE:** Journal name abbreviations are correct.

* Where website addresses are cited, please include the complete URL and specify the date of access (e.g. [accessed: 12/06/2023]).

**RESPONSE:** We include full URLs and access dates where applicable.

* Please also see https://journals.plos.org/plosmedicine/s/submission-guidelines#loc-references for further details on reference formatting.

STUDY TYPE-SPECIFIC REQUESTS

MODELLING STUDIES

The following list is derived from Geoffrey P Garnett, Simon Cousens, Timothy B Hallett, Richard Steketee, Neff Walker. Mathematical models in the evaluation of health programmes. (2011) Lancet DOI:10.1016/S0140-6736(10)61505-X:

* Please provide a diagram that shows the model structure, including how the natural history of the disease is represented, the process and determinants of disease acquisition, and how the putative intervention could affect the system.

**RESPONSE:** The model diagram is included in Figure 1.

* Please provide a complete list of model parameters, including clear and precise descriptions of the meaning of each parameter, together with the values or ranges for each, with justification or the primary source cited and important caveats about the use of these values noted.

**RESPONSE:** Model parameters are described in Tables 1-2 and Tables S1-S2.

* Please provide a clear statement about how the model was fitted to the data, including goodness-of-fit measure, the numerical algorithm used, which parameter varied, constraints imposed on parameter values, and starting conditions.

**RESPONSE:** We include model fitting information in the main Methods (line 161):
“The model was separately calibrated for Brazil, Colombia, and Peru to country-specific data from 1990-2023. Data targets included prevalence of incarceration, prison admission rates, recidivism, population-wide tuberculosis notifications and incidence, and within-prison tuberculosis notifications and incidence[4]. Calibration targets for prison incidence were based on a previous study using Bayesian meta-regression modeling to estimate within-prison case-detection ratios (**Appendix S1**)[3].”

We elaborate in the Appendix: “The model was calibrated with *optim* in R using limited memory BFGS, a quasi-Newton algorithm, with box constraints. Incarceration-related parameters were calibrated first with a loss function that minimized the mean squared percentage error across incarceration prevalence, recidivism, and admissions. Remaining parameters were calibrated by minimizing the mean percentage error across prison TB incidence, population TB incidence, prison TB notifications, and population TB notifications. Calibrated parameters are indicated in **Table S1**. Additional details on model calibration are provided in Liu et al. *Lancet Public Health* 2024[2], from which calibrated parameters were sourced directly.”

* For uncertainty analyses, please state the sources of uncertainties quantified and not quantified [can include parameter, data, and model structure].

**RESPONSE:** We describe the sources of uncertainties quantified and not quantified in the main Methods (line 253):

“**Uncertainty and sensitivity analyses**

We sampled 1000 sets of intervention parameters and cost inputs from uncertainty distributions using Latin Hypercube sampling. These sets were randomly paired with 1000 sets of fitted model parameters, calibrated to data targets also sampled from uncertainty distributions[4]. We repeated the analysis for each set of parameters and inputs, generating a distribution for each outcome, from which we report summary values (mean or median) and 95% uncertainty intervals. For the cost-effectiveness analysis, we assessed joint uncertainty in additional costs (relative to the base case scenario) and DALYs averted for each strategy. We also conducted a probabilistic sensitivity analysis and generated cost-effectiveness acceptability curves, depicting the probability each strategy yields the greatest net monetary benefit at varying willingness-to-pay thresholds. Uncertainty in model structure was not quantified.”

* Please provide sensitivity analyses to identify which parameter values are most important in the model. Uncertainty estimates seek to derive a range of credible results on the basis of an exploration of the range of reasonable parameter values. The choice of method should be presented and justified.

**RESPONSE:** We provide several sensitivity analyses as described below:

“We performed several additional sensitivity analyses. First, we examined how variation in within-prison tuberculosis incidence could affect the optimal screening strategy. We generated scenarios with lower prison incidence by varying within-prison transmission rates, representing variation in prison conditions (i.e. crowding, ventilation). We then conducted multinomial logistic regression and estimated the probability that each strategy would be optimal across the range of prison incidence levels. Second, we re-examined the efficiency frontier and preferred strategy in a scenario without access to CXR-CAD technology, which remains the current reality for most prisons in low- and middle-income countries. Third, repeated intensive screening may lead to earlier detection and treatment, such that the clinical and bacteriological profile of TB in the screened population may shift over time toward less severe disease (i.e., higher proportions of asymptomatic and/or bacteriologically negative TB). To account for this, we gradually decreased test sensitivity for both screening and follow-up testing over the first half of the intervention period, assuming that after five years of intensive screening, test sensitivity converges to values consistent with asymptomatic TB. Details on sensitivity analyses are provided in the **Appendix S1**.”

* Please discuss the scientific rationale for the choice of model structure and identify points where this choice could influence conclusions drawn. Please also describe the strength of the scientific basis underlying the key model assumptions.

**RESPONSE:** We describe model structure explicitly and cite literature supporting assumptions throughout the Methods (line 141):

“**Transmission model and key assumptions**

We extended a published compartmental transmission model simulating tuberculosis and incarceration dynamics (**Figure 1**)[4]. The model includes a simple representation of tuberculosis natural history for the population 15 years of age and older…The model is not stratified by age, gender/sex, or HIV. We simplify the documented spectrum of TB disease states[14], assuming a single disease state (**I**) representing infectious, bacteriologically positive tuberculosis, of which 51% (range 30-70%) is assumed to be symptomatic (**Table 1**). Multi-drug resistant (MDR) tuberculosis was not modeled explicitly but was accounted for by adjusting treatment outcomes and costs based on relative proportions of drug-sensitive and drug-resistant tuberculosis…The elevated risk of tuberculosis in prisons is operationalized through higher effective contact rates, higher disease progression rates, and lower diagnosis rates in prisons than in the community[4]. Individuals with recent incarceration history are assumed to have similar contact rates as never incarcerated individuals, but higher disease progression rates and lower diagnosis rates[4]. The transition from recent to distant incarceration history occurs on average at seven years following release, after which rates of contact, progression, and diagnosis match those of never incarcerated individuals[12].”

We also discuss how model structure decisions could affect conclusions in the limitations section of the Discussion (line 606):

“There are several limitations to note. Our model did not include age, gender, or HIV, although HIV prevalence is relatively low in these countries[28]. We did not model the spectrum of tuberculosis disease states, which may be particularly relevant under repeated screening if the distribution of TB disease shifts towards less detectable forms over time. In a sensitivity analysis assuming declining test positivity among those with tuberculosis, intensive screening averted slightly fewer DALYs at similar or moderately higher cost. This suggests that our simplified representation of TB disease states may modestly underestimate ICERs but likely does not change conclusions regarding the cost-effectiveness of intensive screening with CXR-CAD. In extremely high incidence prisons where our main results suggest that screening with both symptoms and CXR-CAD would be justified, a shift toward predominantly asymptomatic disease would weaken this justification. Relatedly, we did not account for bacteriologically negative tuberculosis, which constitutes a substantial proportion of diagnoses in some settings[1, 29]; future work might explore how inclusion of empirical treatment in prison screening affects impacts and cost-effectiveness. Our simplified approach to representing MDR-TB does not capture complexities of longer treatment regimens or diagnostic delays, which may influence preferred screening strategies. A study modeling tuberculosis screening in prisons in the former Soviet Union, where MDR-TB rates are high, found that annual screening with Xpert was more effective in reducing MDR-TB prevalence compared to annual radiographic screening[24]. Pooled Xpert may be a useful tool in high MDR-TB prisons but should be tested empirically. Furthermore, we did not explicitly model prison transfers, which could reduce effectiveness if screening is not coordinated across facilities. This may partly explain the discrepancy between our results and a recent empirical study in Brazil, which found no reduction in tuberculosis prevalence after three rounds of prison mass screening between 2017 and 2021[30]. In that study, screening was limited to three of 29 male prisons in the state, and frequent transfers likely diluted impact. Future work might examine how inter-facility movement and variation in screening practices across facilities affect intervention effectiveness. Finally, we did not model heterogeneity in infectiousness. If a small fraction of individuals accounts for most transmission, imperfect screening coverage may allow continued transmission.”

* For studies that develop a prediction model or evaluate its performance, please ensure that the study is reported according to the TRIPOD statement (https://www.equator-network.org/reporting-guidelines/tripod-statement) and include the completed checklist as Supporting Information. Please add the following statement, or similar, to the Methods: "This study is reported as per the Transparent Reporting of a Multivariable Prediction Model for Individual Prognosis Or Diagnosis (TRIPOD) statement (S1 Checklist)." For studies using machine learning, please use the TRIPOD-AI checklist. When completing the checklist, please use section and paragraph numbers, rather than page numbers.

**RESPONSE:** N/A, this study does not involve a prediction model.

HEALTH ECONOMICS / COST-EFFECTIVENESS STUDIES

* Please ensure that the study is reported according to the CHEERS guideline (available from: https://www.equator-network.org/reporting-guidelines/cheers) and include the completed checklist as Supporting Information. Please add the following statement, or similar, to the Methods: "This study is reported as per the Strengthening the Consolidated Health Economic Evaluation Reporting Standards 2022 (CHEERS 2022) Statement (S1 Checklist)." When completing the checklist, please use section and paragraph numbers, rather than page numbers.

**RESPONSE:** We now report the study according to the CHEERS guideline, included as Checklist S2.

Ref: Comparative impacts and cost-effectiveness of tuberculosis active case-finding strategies in prisons in Brazil, Colombia, and Peru: a mathematical modeling study

PMEDICINE-D-25-02987R1

Dear Ms. Liu,

Thank you for your patience while we performed technical checks on your submission. Please see below for a list of requirements for you to address while conducting your revisions.

Journal Requirements:

1) Please provide separate figure files in .tif or .eps format. For more information about figure files please see our guidelines:

<https://urldefense.com/v3/__https://journals.plos.org/plosmedicine/s/figures__;!!G92We9drHetJ8EofZw!dlOeRWrO6mn9aHr9xMOPjAo1vUbibWGrvR0HxP4se58G976avOzSA0bVpEafxEOFp3zVxAQuDcH9TPRe1XAc$>

**RESPONSE:** We have converted figure files to .tif using the NAAS tool.

2) Figure S3: please (a) provide a direct link to the base layer of the map (i.e., the country or region border shape) and ensure this is also included in the figure legend; and (b) provide a link to the terms of use / license information for the base layer image or shapefile. We cannot publish proprietary or copyrighted maps (e.g. Google Maps, Mapquest) and the terms of use for your map base layer must be compatible with our CC BY 4.0 license.

Note: if you created the map in a software program like R or ArcGIS, please locate and indicate the source of the basemap shapefile onto which data has been plotted.

If your map was obtained from a copyrighted source please amend the figure so that the base map used is from an openly available source. Alternatively, please provide explicit written permission from the copyright holder granting you the right to publish the material under our CC BY 4.0 license.

If you are unsure whether you can use a map or not, please do reach out and we will be able to help you. The following websites are good examples of where you can source open access or public domain maps:

* U.S. Geological Survey (USGS) - All maps are in the public domain. (https://urldefense.com/v3/__http://www.usgs.gov__;!!G92We9drHetJ8EofZw!dlOeRWrO6mn9aHr9xMOPjAo1vUbibWGrvR0HxP4se58G976avOzSA0bVpEafxEOFp3zVxAQuDcH9TFxjLzWU$ )

* PlaniGlobe - All maps are published under a Creative Commons license so please cite “PlaniGlobe, <https://urldefense.com/v3/__http://www.planiglobe.com__;!!G92We9drHetJ8EofZw!dlOeRWrO6mn9aHr9xMOPjAo1vUbibWGrvR0HxP4se58G976avOzSA0bVpEafxEOFp3zVxAQuDcH9TG21QhA7$> , CC BY 2.0” in the image credit after the caption. (https://urldefense.com/v3/__http://www.planiglobe.com/?lang=enl__;!!G92We9drHetJ8EofZw!dlOeRWrO6mn9aHr9xMOPjAo1vUbibWGrvR0HxP4se58G976avOzSA0bVpEafxEOFp3zVxAQuDcH9TFEQeend$ )

* Natural Earth - All maps are public domain. (https://urldefense.com/v3/__http://www.naturalearthdata.com/about/terms-of-use/__;!!G92We9drHetJ8EofZw!dlOeRWrO6mn9aHr9xMOPjAo1vUbibWGrvR0HxP4se58G976avOzSA0bVpEafxEOFp3zVxAQuDcH9TDT8irU_$ )

**RESPONSE:** We have added the following to the Figure S3 legend: “Country borders obtained from Natural Earth, accessed via the maps R package. Available at<https://www.naturalearthdata.com>.”

3) We have noticed that you have uploaded Supporting Information files, but you have not included a list of legends. Please add a full list of legends for your Supporting Information files after the references list.

**RESPONSE:** We have now included a list of legends for the Supporting Information files, after the References.

If you have any questions or require assistance, please don't hesitate to get in touch via return email. To help us minimize inbox volumes, we kindly ask that you not reply to this email simply to confirm you have resubmitted your revisions.

Kind regards,

Heba Abdelsabour

Publications Assistant

PLOS Medicine

[plosmedicine@plos.org](mailto:plosmedicine@plos.org)
