# Supplementary material for: Comparative impacts and cost-effectiveness of tuberculosis systematic screening strategies in prisons in Brazil, Colombia, and Peru: A mathematical modeling study
Source: PLoS Med. 2026 Jun 4;23(6):e1004739. doi: 10.1371/journal.pmed.1004739 (PMC13258141; doi:10.1371/journal.pmed.1004739)
Supplement: Attachment — Submitted filename: ResponseToReviewersEditors_Round2.docx [file pmed.1004739.s004.docx]

Requests from Editors:

Abstract and Author Summary
* Please ensure that the Introduction ends with a clear description of the study question or hypothesis.

**Response:** We have added the following sentence at the end of the Background section of the Abstract: “We examined the population-wide health impacts and cost-effectiveness of systematic screening in prisons in Brazil, Colombia, and Peru, comparing different timepoints, frequencies, and screening algorithms.” We have also modified the last sentence of the introduction of the Author Summary to more clearly state the study question: “The World Health Organization strongly recommends systematic screening for tuberculosis in prisons, but evidence is needed to inform how often to screen, which methods to use, and whether these approaches are cost-effective across different country contexts.”

* Please ensure that all abbreviations are defined at first use throughout the text.

**Response**: We have reviewed the manuscript and ensured that all abbreviations are defined at first use in the text.

* Please confirm that all numbers presented in the abstract are present and identical to numbers presented in the main manuscript text.

**Response**: We have confirmed that all numbers in the Abstract are consistent with numbers in the main text. We corrected the range of prison incidence reduction from "62-87%" to "61-87%" to match the point estimate of 61.4% for Peru (Table S2). All other numbers were verified to be identical or consistent with appropriate rounding.

* In the last sentence of the Abstract Methods and Findings section, please describe the main limitation(s) of the study's methodology.

**Response**: We have added this sentence to the end of the Abstract Methods and Findings section: “Key limitations include the model's simplified representation of tuberculosis disease states and lack of stratification by age, gender/sex, HIV, or drug resistance.**”**

* In the author summary, in the final bullet point of 'What Do These Findings Mean?', please include the main limitations of the study in non-technical language.

**Response**: We have added the following: “The main limitations of the study include a simplified representation of how tuberculosis develops in individuals and spreads across populations. Modeling results should be interpreted alongside empirical evidence that incorporates real-world complexities.**”**

Line 94: suggest to replace 'algorithm' with 'screening strategy'.

**Response**: We have replaced “algorithm” with “screening method”.

Figures and Tables
There is a formatting error in Table 3, upper right

**Response**: We have corrected the formatting error.

Please add footnotes to Tables defining all abbreviations used.

**Response**: We have added footnotes to Tables defining all abbreviations used.

If it is not feasible to make the y-axes the same among panels within the same figure (e.g. Fig. 3A, 3B (x and y axes in B), and the upper and lower panels of Fig.2), please acknowledge the discrepancies in the figure legends.

**Response**: We have added a note to the legends of Figures 2 and 3 noting the discrepancies in axis scales.

* Please define all elements of box plots in the figure caption - center line, box limits and whiskers.

**Response:** We have added this to the Figure 2 legend: “Boxplots show the median (center line), interquartile range (box limits), and 1.5*interquartile range (whiskers) across 1000 model simulations and parameter sets per country.”

* Where data points are discrete, please ensure that they are depicted in the figures as discrete data and not as a continuous line.

**Response**: We have reviewed all figures and ensured that discrete data points are depicted as such. In the cost-effectiveness planes (Figure 3B), individual strategies are shown as discrete points, with only the efficiency frontier connecting non-dominated strategies. We have clarified this in the legend as follows: “The black line indicates the efficiency frontier”. Note, in Figure 4, continuous probability curves are derived from multinomial logistic regression with prison incidence as a continuous predictor; the smooth lines therefore represent the fitted model’s predicted probabilities.

* Please provide the unadjusted comparisons as well as the adjusted comparisons in all relevant Tables.

**Response:** As this is a modeling study rather than an observational analysis, there are no adjusted versus unadjusted statistical comparisons in the traditional sense. If the editors intended a different comparison, we would welcome further clarification.

Methods
Please add a Statistical Analyses subsection to the Methods.

**Response:** We appreciate this suggestion. As this is a mathematical modeling study rather than an empirical analysis, the analytical methods are inherently integrated with the model structure, intervention design, and economic evaluation. We believe that consolidating these into a single "Statistical Analyses" subsection would disrupt the logical flow of the Methods, which is currently organized to follow modeling best practices (per Garnett et al., Lancet 2011). Instead, we have ensured that all relevant statistical details—including the calibration approach, sampling methodology, summary statistics reported, discounting, ICER estimation, and probabilistic sensitivity analysis—are clearly described within the existing subsections (Transmission model, Costs and outcomes, and Uncertainty and sensitivity analyses).

* Please follow this guidance for Statistical reporting:
Please report statistical information as follows to improve clarity for the reader "22% (95% CI [13,28]; p</=)".

- Please separate upper and lower bounds with commas instead of hyphens as the latter can be confused with reporting of negative values.

- Please repeat statistical definitions (HR, CI etc.) for each set of parentheses.

**Response:** We have revised all statistical reporting to follow the requested format: using commas to separate upper and lower bounds of intervals (replacing hyphens), and repeating the statistical term (95% UI) in each set of parentheses. As this is a modeling study, we report 95% uncertainty intervals (UIs) derived from probabilistic uncertainty analysis rather than confidence intervals or p-values from hypothesis testing.

Please include a clean version of the supporting information.

**Response:** We now include a clean version of the supporting information.

Please move the Disclaimer out of the funding statement and add a Disclaimer subsection.

**Response:** Done.

Please create a DOI for your data and code and add to the Data Availability Statement.

**Response:** We have created a DOI for the data and code and added it to the Data Availability Statement: “Data and code for the analyses in the manuscript can be found at <https://doi.org/10.5281/zenodo.19636053>.”

Competing interests:
* Please add this statement to the manuscript's Competing Interests: "JG-F is an Academic Editor on PLOS Medicine's editorial board."
* All authors must declare their relevant competing interests per the PLOS policy, which can be seen here: https://journals.plos.org/plosmedicine/s/competing-interests For authors with ties to industry, please indicate whether any of the interests has a financial stake in the results of the current study.
Authors MCCA, BM, MAG, JTT, CAMR, AFMR, CB, PAJ, DMP, FDCJ have not completed their COI questionnaire.

**Response:** We have added that statement to the Competing Interests statement, and have contacted all the listed authors to urge them to complete their COI questionnaire. If it is possible to re-send the link, that would be appreciated.

Other:
* Please review your manuscript and edit to ensure compliance with our inclusive language requirements <https://journals.plos.org/plosmedicine/s/human-subjects-research#loc-categorization>

**Response:** We have reviewed the manuscript against PLOS Medicine's inclusive language guidelines and confirm compliance throughout.

* Please include an Acknowledgments section in your manuscript.

**Response:** Added.

Please ensure that statements in the manuscript that also appear in the manuscript metadata are identical.

**Response:** We have ensured that the statements are identical.

For modeling papers, we ask that you follow the guidance from Geoffrey P Garnett, Simon Cousens, Timothy B Hallett, Richard Steketee, Neff Walker. Mathematical models in the evaluation of health programmes. (2011) Lancet DOI:10.1016/S0140-6736(10)61505-X:
* Please provide a diagram that shows the model structure, including how the disease natural history is represented, the process and determinants of disease acquisition, and how the putative intervention could affect the system.

**Response:** This is provided in Figure 1.

* Please provide a complete list of model parameters, including clear and precise descriptions of the meaning of each parameter, together with the values or ranges for each, with justification or the primary source cited, and important caveats about the use of these values noted.

**Response:** These are provided in Tables 1-2 and Tables S1-S2.

* Please provide a clear statement about how the model was fitted to the data including where relevant goodness-of-fit measure, the numerical algorithm used, which parameter varied, constraints imposed on parameter values, and starting conditions.

**Response:** This is included in the Methods and in the Appendix:

“Calibrated parameters were sourced directly from a prior study[4], wherein the model was separately calibrated for Brazil, Colombia, and Peru to country-specific data from 1990-2023. Data targets included prevalence of incarceration, prison admission rates, recidivism, population-wide tuberculosis notifications and incidence, and within-prison tuberculosis notifications and incidence[4]. Calibration targets for prison incidence were based on a previous study using Bayesian meta-regression modeling to estimate within-prison case-detection ratios (**Appendix S1**)[3]. Model fit to data is shown in **Figures S1-S2**.”

“The model was calibrated with *optim* in R using limited memory BFGS, a quasi-Newton algorithm, with box constraints. Incarceration-related parameters were calibrated first with a loss function that minimized the mean squared percentage error across incarceration prevalence, recidivism, and admissions. Remaining parameters were calibrated by minimizing the mean percentage error across prison TB incidence, population TB incidence, prison TB notifications, and population TB notifications. Calibrated parameters are indicated in **Table S1**. Additional details on model calibration are provided in Liu et al. *Lancet Public Health* 2024[2], from which calibrated parameters were sourced directly.”

* For uncertainty analyses, please state the sources of uncertainties quantified and not quantified this can include parameter, data, and model structure.

**Response**: We have added this statement to the Methods to clarify: “We quantified uncertainty in data targets, epidemiologic parameters, intervention parameters, cost inputs, and model assumptions, but not in model structure.”

* Please provide sensitivity analyses to identify which parameter values are most important in the model. Uncertainty estimates seek to derive a range of credible results on the basis of an exploration of the range of reasonable parameter values. The choice of method should be presented and justified.

**Response:** We have conducted several sensitivity analyses. Rather than a traditional one-way sensitivity analysis, which would be difficult with the extensive list of parameters in this analysis, we conducted scenario-based sensitivity analyses targeting the parameters and assumptions identified as most likely to influence conclusions: within-prison transmission rates (which drive prison incidence, the single most important determinant of cost-effectiveness), availability of CXR-CAD technology, and test sensitivity under repeated screening. These were selected based on their policy relevance and their expected influence on model outcomes, as well as in response to reviewers’ comments. The probabilistic sensitivity/uncertainty analysis additionally captures the joint influence of all uncertain parameters through 1,000 sampled parameter sets.

* Please discuss the scientific rationale for this choice of model structure and identify points where this choice could influence conclusions drawn. Please also describe the strength of the scientific basis underlying the key model assumptions.

**Response:** We believe the revised manuscript addresses this point substantively. The Methods section describes the model structure and justifies key design choices. The Discussion's limitations paragraph identifies specific points where structural choices could influence conclusions, including the absence of age/sex/HIV stratification, the simplified representation of TB disease states and MDR-TB, the lack of explicit prison transfer dynamics, and the absence of heterogeneity in infectiousness. Additionally, we conducted sensitivity analyses directly targeting the structural assumptions most likely to affect conclusions (declining test sensitivity under repeated screening, varying prison incidence levels). The model structure and calibration approach are further detailed in Appendix S1.

Comments from Reviewers:

Reviewer #2: Thank you for providing this excellent update of your manuscript. I think you have addressed my and the other reviewer comments thoroughly and thoughtfully.

I have 2 trivial comments related to figures in the appendix.

**Response:** We thank the reviewer for their helpful comments to improve the manuscript.

Figure S7: Suggest you indicate in the legend that the straight lines in each panel reflect the WTP threshold.

**Response:** Thanks to the reviewer for catching this omission. We have now added that clarification to the Figure S7 legend: “The straight black line in each panel indicates the willingness-to-pay threshold.”

Figure S8: The legend mentions open squares, but I didn't see these in the figure.

**Response:** We apologize for this error. The updated version of Figure S8 now includes the open squares indicating the cost-effectiveness acceptability frontier.

**Figure S8. Cost-effectiveness acceptability curves and frontier.** Curves show the proportion of all iterations in which each strategy yielded the greatest net monetary benefit across a range of willingness-to-pay thresholds. Open squares indicate the cost-effectiveness acceptability frontier, comprised of the optimal strategy at each willingness-to-pay threshold (based on the expectation of net monetary benefit across iterations). The vertical dashed line indicates each country’s cost-effectiveness threshold.

Reviewer #3: I want to thank the authors for the thoughtful and thorough response to our comments. It is an excellent paper. Good to go for me.

**Response:** We thank the reviewer for their helpful comments to improve the manuscript.

Reviewer #4: Here Liu et al. extend a previously developed mathematical model of TB incidence in prison populations in Brazil, Colombia, and Peru. I was asked to review this manuscript as a statistical reviewer during revision.
My overall impression is that the analysis and modeling are thorough, handling of key limitations is pragmatic and addressed in the discussion. The results are interesting and could be of important use for implementation of such interventions. Implicit in this work is how diagnostic specificity/sensitivity for TB detection impacts the cost and epidemiology of managing TB.

**Response:** We thank the reviewer for their positive feedback and constructive comments to improve the manuscript.

My comments are fairly minor.
My main comment with the model is regarding the assumption that contact rates are the same between recently incarcerated and never incarcerated. This is a strong assumption and it is plausible that recently incarcerated populations are particularly sensitive and could be at a higher risk. This is very briefly discussed in [4], but the implications of this assumption could be compounded here since the impact of screening lags into this cohort. This should at least be mentioned in the discussion.

**Response:** We appreciate the reviewer pointing this out and have added this sentence to the limitations: “We assumed equivalent effective contact rates across community-based strata; if recently incarcerated individuals have higher contact rates, the downstream transmission effects of prison-based screening might be underestimated.” (line 513)

How are the interventions implemented into the structural model simulation? For example, is the bi-annual screening treated as a continuous process (constant rate) or discrete (pulse process, stiff system)?
- I would find it helpful if the appendix explained the technical differences between interventions; for example, by comparing the annual vs. biannual implementations.

**Response:** We appreciate the opportunity to clarify. We have added details about the implementation of screening interventions in the Appendix:
“Periodic prison-wide screening (annual or biannual) was implemented as a discrete pulse process, with screening activated during rounds of fixed duration (assumed to be two months each) and deactivated between rounds. For annual screening, one round occurs per year; for biannual screening, two rounds occur per year, with the start of each round spaced six months apart. Other than frequency of rounds, screening rates and per-round coverage and duration are assumed to be identical between annual and biannual screening.

Entry screening is implemented as a modifier on the flow of individuals entering prison. A fraction of infectious individuals entering prison (from community strata) are detected and treated at the point of entry, transitioning directly to $R_{p}.$ Entry screening also moves a fraction of individuals entering prison from $E$ compartments directly to $L_{p}$. Screening of individuals transferred between prisons is modeled analogously, as a continuous rate proportional to the transfer rate.

Exit screening is implemented similarly as a modifier on the flow of individuals leaving prison, with detected and treated individuals transitioning directly from $I_{p}$ to $R_{r}$, and from $E_{p}$ to $L_{r}$.”

Explicitly state your definition of a false positive. I assumed a false positive is any non-infected individual (specifically S) who screens positive.
- Related, why would test-positive individuals in the early latent (E) state who are treated be classified as a false positive? It seems like an unintended benefit to catch TB in these populations with mass screening

**Response:** We appreciate these points from the reviewer. We have added some clarification to specify that a false positive is any individual without active, infectious disease, including those with latent infection: “Individuals in **E** who test false positive for infectious tuberculosis disease through systematic screening and complete treatment transition to **L** (**Appendix S1**).” We have also added additional clarification to the Appendix:
“*False positives*

Our screening test accuracy parameters (sensitivity and specificity) are derived from empirical studies using bacteriologically confirmed active tuberculosis as the reference standard. Therefore, we define a false positive as any individual without active, infectious tuberculosis disease (compartment $I$) who tests positive through systematic screening, including individuals with latent infection.

Detecting and treating individuals in $E$ could confer an unintended benefit by clearing early latent infection. In the model, treated false positives in $E$ transition to $L$, reflecting the effect of completing a course of treatment for active disease. We chose to transition these individuals to $L$ rather than $R$ because, in our model structure, individuals in $R$ can relapse to $I$ without reinfection, which would not be appropriate for individuals who never developed active disease. Moving treated $E$ individuals to $L$ captures the benefit of reduced activation risk while avoiding this structural inconsistency.”

- Discussion point: There's an implicit assumption that false positives go through a full confirmatory work-up, but it's possible that could be stopped early (eg, after a culture negative test). The approach may overestimate costs in this algorithm.

**Response:** This is a good point, which we have now addressed in the Limitations (line 516): “We assumed that all false positives receive a full confirmatory work-up and treatment; in practice, some may be ruled out earlier (e.g., after a negative culture), and our approach may therefore overestimate costs.”

I inferred that the Xpert pool algorithm is hampered by the loss of sensitivity due to pooling 8 samples. Is that a reasonable conclusion? Could the authors comment on using smaller pool sizes. Perhaps it's not cost effective but might be worth stating that.

**Response:** We have commented briefly on the pool size in the Methods as follows (line 205:

“For sputum pooling, samples from eight individuals (the most efficient pool size based on prior work) are combined and tested using a single Xpert cartridge; we assume that pooling reduces Xpert sensitivity but does not affect specificity[9]. Smaller pools may yield increased sensitivity at greater cost.”

Figure 1, the color arrow intervention implies only I -> R, but I assume any treated infected (E, L) individual moves to R?

**Response:** We apologize for the confusion. We assume the intervention also moves individuals in E who test positive and receive treatment to L; we have now added arrows indicating these transitions, and we have added this to the Figure 1 legend to clarify (see below).

**Figure 1. Compartmental transmission model schematic.** The model includes five tuberculosis disease states: susceptible (S), early latent infection (E), late latent infection (L), infectious active disease (I), recovered (R). These disease states are replicated across four incarceration-related population strata, denoted with subscripts: prison (p), recent history of incarceration (r), distant history of incarceration (d), never incarcerated (n). Solid black arrows represent tuberculosis natural history transitions (e.g. infection, progression to disease, recovery, relapse). Dashed black arrows represent transitions between population strata (e.g. incarceration, release, re-incarceration). Solid colored arrows indicate transitions resulting from systematic screening interventions, which move individuals from infectious active disease (I) to recovered (R). Hollow (double-lined) colored arrows indicate additional transitions resulting from screening wherein individuals in early latent infection (E) who test false positive and receive treatment for infectious, active disease move to late latent infection (L). Entry screening (including screening upon inter-prison transfer) is shown in gold; exit screening is shown in purple, and prison-wide screening (annual or biannual) is shown in orange. For entry and exit screening, recovery from disease occurs concurrently with incarceration-related transitions.

Figure 2, what do the boxplot distributions represent (e.g., each model simulation, with what N), and were outliers present?

**Response:** We appreciate the opportunity to clarify. We have added the following description to the Figure legend:

“**Figure 2. Impacts of screening interventions on within-prison and population-wide tuberculosis incidence in 2035.** Boxplots show the median (center line), interquartile range (box limits), and 1.5*interquartile range (whiskers) across 1000 model simulations and parameter sets per country. Outliers are not shown. Note: y-axis scales differ between the upper and lower panel. CXR-CAD, chest X-ray with computer-aided detection.”

The calibration was somewhat unclear to me: The methods read as if the models were re-calibrated for this manuscript using the methodology in [4], while the appendix states the calibrated parameters were sourced directly from [4]. Please clarify which parameters were re-used and which were re-calibrated, this could be simply annotated with minor editing to Table S1.

**Response:** Apologies for the lack of clarity. The parameters were indeed sourced from the prior published work. We have added this to the Methods: “Calibrated parameters were sourced directly from a prior study[4], wherein the model was separately calibrated for Brazil, Colombia, and Peru to country-specific data from 1990-2023.” (line 163)

I found the following sentence too dense: "We then conducted multinomial logistic regression and estimated the probability that each strategy would be optimal across the range of prison incidence levels." I suggest expanding on this in the Appendix so that it is reproducible. What is the input data (n and what each observation unit represents), the dependent multinomial variable, the predictors, key quantitative outcomes extracted from the model, and how it was fit (software)?

**Response:** We have modified this sentence in the main text to improve clarity (line 277): “We then conducted multinomial logistic regression and estimated the probability that each strategy would be optimal under varying prison incidence levels.”

We then expand on this analysis in the Appendix as follows: “For the first sensitivity analysis, we generated a wide range of within-prison tuberculosis incidence values by multiplying the previously calibrated effective contact rate in prison by a sampled factor 0.1 < *f* < 1 for each of the 1000 parameter sets per country. A constraint was then imposed to ensure that the prison effective contact rate in prison remained greater than or equal to the community effective contact rate. After re-simulating screening interventions, we identified the optimal strategy, defined as the most impactful, non-dominated strategy with an incremental cost-effectiveness ratio (ICER) within the country’s cost-effectiveness threshold. For each country, using the results from 1000 model simulations, we fit a multinomial logistic regression model using multinom from the nnet package in R[10], with the optimal strategy as the dependent variable and within-prison incidence as the predictor variable. Each observation corresponded to one simulation (i.e., one parameter set with its associated prison incidence level and identified optimal strategy), yielding 1,000 observations per country. We then used this model to predict the probability of each strategy being optimal across the incidence range.”
