# Supplementary material for: Comparative impacts and cost-effectiveness of tuberculosis systematic screening strategies in prisons in Brazil, Colombia, and Peru: A mathematical modeling study
Source: PLoS Med. 2026 Jun 4;23(6):e1004739. doi: 10.1371/journal.pmed.1004739 (PMC13258141; doi:10.1371/journal.pmed.1004739)
Supplement: Peer review history

# Editor report: Decision Letter 0

**DOI:** 10.1371/journal.pmed.1004739.r001

**Authors:** Alison Farrell

26 Aug 2025

Dear Dr Liu,

Thank you for submitting your manuscript entitled "Comparative impacts and cost-effectiveness of tuberculosis active case-finding strategies in prisons in Brazil, Colombia, and Peru: a mathematical modeling study" for consideration by PLOS Medicine.

Your manuscript has now been evaluated by the PLOS Medicine editorial staff as well as by an academic editor with relevant expertise and I am writing to let you know that we would like to send your submission out for external peer review.

However, before we can send your manuscript to reviewers, we need you to complete your submission by providing the metadata that is required for full assessment. To this end, please login to Editorial Manager where you will find the paper in the 'Submissions Needing Revisions' folder on your homepage. Please click 'Revise Submission' from the Action Links and complete all additional questions in the submission questionnaire.

For clinical studies, please upload a copy of your trial study protocol as a supporting information file. The study protocol should be the version submitted for approval to the institutional review board or ethics committee, should include any amendments to the study protocol, as well as the date of their approval by the institutional review or ethics committee. Please also detail any deviations from the study protocol in the Methods section of your manuscript. The editors will consider the protocol and study conduct prior to a final decision for external review.

Please re-submit your manuscript within two working days, i.e. by Aug 28 2025 11:59PM.

Login to Editorial Manager here: https://www.editorialmanager.com/pmedicine

Once your full submission is complete, your paper will undergo a series of checks in preparation for peer review. Once your manuscript has passed all checks it will be sent out for review.

Feel free to email us at plosmedicine@plos.org if you have any queries relating to your submission.

Kind regards,

Alison Farrell, Ph.D.

Senior Editor

PLOS Medicine

# Peer review: Decision Letter 1

**DOI:** 10.1371/journal.pmed.1004739.r002

**Authors:** Alison Farrell

29 Jan 2026

Dear Dr Liu,

Many thanks for submitting your manuscript "Comparative impacts and cost-effectiveness of tuberculosis active case-finding strategies in prisons in Brazil, Colombia, and Peru: a mathematical modeling study" (PMEDICINE-D-25-02987R1) to PLOS Medicine. The paper has been reviewed by subject experts and a statistician; their comments are included below and can also be accessed here: [LINK]

As you will see, the reviewers find the study interesting and potentially important, and have made suggestions to improve the presentation, clarity and strength of the findings. After discussing the paper with the editorial team and an academic editor with relevant expertise, I'm pleased to invite you to revise the paper in response to the reviewers' and editors' comments. We plan to send the revised paper to some or all of the original reviewers, and we cannot provide any guarantees at this stage regarding publication.

In addition to these revisions, you may need to complete some formatting changes, which you will receive in a follow up email. A member of our team will be in touch with a set of requests shortly. If you do not receive a separate email within a few days, please assume that checks have been completed, and no additional changes are required.

When you upload your revision, please include a point-by-point response that addresses all of the reviewer and editorial points, indicating the changes made in the manuscript and either an excerpt of the revised text or the location (eg: page and line number) where each change can be found. Please also be sure to check the general editorial comments at the end of this letter and include these in your point-by-point response. When you resubmit your paper, please include a clean version of the paper as the main article file and a version with changes tracked as a marked-up manuscript. It may also be helpful to check the guidelines for revised papers at http://journals.plos.org/plosmedicine/s/revising-your-manuscript for any that apply to your paper.

We ask that you submit your revision by Feb 19 2026 11:59PM. However, if this deadline is not feasible, please contact me by email, and we can discuss a suitable alternative.

Don't hesitate to contact me directly with any questions (afarrell@plos.org).

Best regards,

Alison

Alison Farrell, Ph.D.

Senior Editor

PLOS Medicine

afarrell@plos.org

-----------------------------------------------------------

Comments from the academic editor:

Reporting the composition of costs for different strategies would help contextualize these results from a planning perspective. A stacked bar chart showing the breakdown of costs on the efficiency frontier would be particularly useful- specifically, the proportion of costs attributable to screening (by screen), follow-up diagnostics, correct treatment, and 'unnecessary treatment' (only for those false positives on both screening and diagnostic tests). This breakdown would clarify which cost components increase or decrease across strategies, providing practical insights for resource allocation and implementation planning.

-----------------------------------------------------------

Comments from the reviewers:

Reviewer #1: This modelling study addresses a fundamentally important research question in tuberculosis control, is very well executed from a technical perspective, and is very nicely written. The results are likely to have immediate and important implications for both public and individual health. Beyond the tuberculosis community, the results will generate broad interest among the readership of PLOS Medicine as they draw attention to the importance of health among incarcerated populations, a vulnerable and neglected population, and show how the health of incarcerated populations matters to the health of the general population. The researchers clearly present very thorough projections based on plausible active case finding interventions. They account for uncertainty and different scenarios (e.g. different incidences, different screening algorithms) very completely. I think the discussion articulates well the key issues around incidence rebounding after the intervention period, the need to implement immediate screening with available tools, and the importance of structural reforms to reduce incarceration and crowding in prisons. I believe the key limitations and weaknesses are acknowledged, and future research priorities are appropriately articulated, including the need for empirical studies.

Congratulations on this important work and thank you for your attention to this matter.

Reviewer #2: This modeling manuscript reports a highly relevant set of analyses predicting the impact and cost-effectiveness of potential screening interventions for TB in the prisons of Brazil, Colombia and Peru. It is clearly written; the analyses have been rigorously executed and provide important and actionable results. The consideration of population-wide epidemiologic impact is of major interest. The authors provide a thoughtful discussion of key points/implications, strengths and limitations of their work. I have relatively few suggestions for improvement.

Major comments:

1. This work builds on earlier modeling, published by Liu et al (Lancet Public Health, 2024). While calibration is well described in the present manuscript, key elements of the model and some important underlying assumptions/parameters are missing. For example, I could not find the TB case fatality rate listed anywhere; this is obviously essential to DALY estimates and needs to be stated clearly in the main text and/or the main tables. My own preference would also be to show the key assumptions related to DALY weights and life expectancy in table form in the main paper, so that readers can understand the main elements of the DALY calculations without having to refer to the appendix. More generally, I think the structure and key flows/rates for the dynamic model should be shown in the appendix, even if this repeats some material in the 2024 paper.

2. The analysis includes identification of the "optimal" screening strategy at varying prison TB incidence (Figure 3). This is very useful. However, how the "optimal" strategy is defined should be clearly spelled out for readers in the methods section—it is not really stated until lines 296-298 in the results section.

3. While there is extensive attention to uncertainty with respect to epidemiologic impact of the various strategies, there is much less exploration of uncertainty with respect to costs and cost-effectiveness. It is reasonable to define the efficiency frontier using the mean values for incremental costs and DALYs as was done (e.g. figure 2). However, the cost-effectiveness planes shown in that figure do not display any ranges around the point estimates, and there are some alternative strategies with point estimates that fall very close to the frontier. Similarly, for the ICER estimates comparing strategies along the efficiency frontier, it would be appropriate to show probabilistic sensitivity analyses—in the form of cost-effectiveness planes graphing the incremental cost and effectiveness for each set of parameters and inputs, and/or cost-effectiveness acceptability curves. These could be added to the appendix.

Minor comment:

In figure 2, some graphs have axes with costs in scientific notation i.e. in exponential form, while some have axes with costs in whole numbers. I'd suggest you convert all to the latter.

Reviewer #3: Dear editor,

This is an important and thorough modelling study around the potential and shape of systematic screening in prisons in Latin America. The work is overall well described, and should help influence important policy decisions in the region. While this is certainly worth publishing and has a good place in PMED, there are a few areas I think the paper could be improved by revision or more explanation, which I outline below.

Abstract:

Slight disconnect between results and conclusions - results do not state that any strategy (including just symptom followed by Xpert) was CE, but conclusion suggest just use any technique. I think the authors mean use pooled Xpert at minimum, then move to CXR-CAD, but it is not clear, and should be. Suggest adding clarification in findings or conclusion in abstract. I think l366-370 cover the relevant results, but the abstract should be self-contained.

Methods

Strongly urge the authors to include a model diagramme in the main text to support the reader's understanding, including the core model and relevant subdivisions.

Regarding model structure, the authors assume a single TB state, with constant test performance in each round of screening (Table 1). That single state summarises a lot of complexity and assumptions (e.g. what proportion is symptomatic, has CXR changes, is bacteriologically positive?) and also assumes that the distribution of severity (in terms of CXR change, symptoms or Xpert) among those 'with TB' remains the same each round over 10 years of intensive systematic screening and substantially dropping burden (figure S4), In reality, I would expect that make up to shift toward less severe disease, and worry that this simplification of TB natural history is a major limitation in the context of examining the impact of repeated screening with different tools, likely overestimating the number of diagnoses and the overall impact. Perhaps not much of an issue in the entry screening, it does affect the other target groups. While it is not reasonable to change the model structure at this point, this issue does need addressing by the authors. I would suggest two approaches: 1) explore a shift in the test positivity values over time to reflect a change in the make up of the screened population 2) discuss this choice and limitation explicitly in the methods, describing the assumed make up of the 'disease' state in terms of bacteriology, symptoms and radiology, and discussion (where it is not currently mentioned in the limitations).

For Table 1 - I have not read through all the references, but it would be good if the authors clarified what the reference standard was in the studies that provided sens and spec. In particular, whether the definition of 'disease' aligns with that used in the model. E.g. was the reference standard culture positive, Xpert positive, did it include clinical assessment in absence of bacteriological test? If they align, then it is helpful to be clear. This is important, as the key results and dominant strategy is almost entirely driven by the assumed high sensitivity (and specificity) of CXR/CAD as compared to other tools (table 1).

L190: is there any consideration of diagnosis of bacteriologically negative individual, e.g. based on CXR abnormalities +/- symptoms. These numbers reach ~50% of all treatment initiations in other screening efforts (e.g. doi 10.1136/bmjgh-2025-019133).

Results

Figure 3 is interesting. Squinting a little it seems the figures are mostly the same, just with more spread out X-axes. It is interesting that the purple line starts to dominate in the very high prison incidences, nice to comment on what model behaviour is driving that.

L309-311 - this annual vs bi-annual is an interesting observation, and less dependent (I think) on the assumptions around test performance. Good to reflect on a bit more in discussion, where it is merely repeated as a finding, but not explained through either the model setup, or how this reflects reality.

There appear no results reported around false positive diagnoses, and how the FP/TP ratio changes between different algorithms, or how they are counted in DALY and costs. Given that they are tracked (l174-176), these results should be reported I think.

Discussion:

Discuss the empirical evidence for this impact. I am aware there are studies ongoing, but good to discuss to what extent these modelled findings can or will be compared against empirical data.

Minor:

Language issue - use of the word 'case'-finding in title and throughout - WHO and StopTB suggest alternative terms, e.g. systematic screening (as used in l108), throughout.

Any attachments provided with reviews can be seen via the following link: [LINK]

--------------------------------------------------------- ---

General editorial requests:

(Note: not all will apply to your paper, but please check each item carefully)

* We ask every co-author listed on the manuscript to fill in a contributing author statement, making sure to declare all competing interests. If any of the co-authors have not filled in the statement, we will remind them to do so when the paper is revised. If all statements are not completed in a timely fashion this could hold up the re-review process. If new competing interests are declared later in the revision process, this may also hold up the submission. Should there be a problem getting one of your co-authors to fill in a statement we will be in contact. Please do not add or remove authors without first discussing this with the handling editor. You can see our competing interests policy here: http://journals.plos.org/plosmedicine/s/competing-interests.

* Please upload any figures associated with your paper as individual TIF or EPS files with 300dpi resolution at resubmission; please read our figure guidelines for more information on our requirements: http://journals.plos.org/plosmedicine/s/figures. While revising your submission, we strongly recommend that you use PLOS's NAAS tool (https://ngplosjournals.pagemajik.ai/artanalysis) to test your figure files. NAAS can convert your figure files to the TIFF file type and meet basic requirements (such as print size, resolution), or provide you with a report on issues that do not meet our requirements and that NAAS cannot fix.

After uploading your figures to PLOS's NAAS tool - https://ngplosjournals.pagemajik.ai/artanalysis, NAAS will process the files provided and display the results in the "Uploaded Files" section of the page as the processing is complete.

If the uploaded figures meet our requirements (or NAAS is able to fix the files to meet our requirements), the figure will be marked as "fixed" above. If NAAS is unable to fix the files, a red "failed" label will appear above.

When NAAS has confirmed that the figure files meet our requirements, please download the file via the download option, and include these NAAS processed figure files when submitting your revised manuscript.

* Please ensure that the paper adheres to the PLOS Data Availability Policy (see http://journals.plos.org/plosmedicine/s/data-availability), which requires that all data underlying the study's findings be provided in a repository or as Supporting Information. For data residing with a third party, authors are required to provide instructions with contact information (web or email address) for obtaining the data. Please note that a study author cannot be the contact person for the data. PLOS journals do not allow statements supported by "data not shown" or "unpublished results." For such statements, authors must provide supporting data or cite public sources that include it.

* We expect all researchers with submissions to PLOS in which author-generated code underpins the findings in the manuscript to make all author-generated code available without restrictions upon publication of the work. In cases where code is central to the manuscript, we may require the code to be made available as a condition of publication. Authors are responsible for ensuring that the code is reusable and well documented. Please make any custom code available, either as part of your data deposition or as a supplementary file. Please add a sentence to your data availability statement regarding any code used in the study, e.g. "The code used in the analysis is available from Github [URL] and archived in Zenodo [DOI link]" Please review our guidelines at https://journals.plos.org/plosmedicine/s/materials-software-and-code-sharing and ensure that your code is shared in a way that follows best practice and facilitates reproducibility and reuse. Because Github depositions can be readily changed or deleted, we encourage you to make a permanent DOI'd copy (e.g. in Zenodo) and provide the URL.

* Please ensure that the study is reported according to the appropriate guideline and include the completed checklist as Supporting Information. When completing the checklist, please use section and paragraph numbers, rather than page numbers. Please add the following statement, or similar, to the Methods: "This study is reported as per [XXXX] guideline (S1 Checklist)."

FORMATTING - GENERAL

* Abstract: Please structure your abstract using the PLOS Medicine headings (Background, Methods and Findings, Conclusions). Please combine the Methods and Findings sections into one section.

* At this stage, we ask that you include a short, non-technical Author Summary of your research to make findings accessible to a wide audience that includes both scientists and non-scientists. The Author Summary should immediately follow the Abstract in your revised manuscript. This text is subject to editorial change and should be distinct from the scientific abstract. Ideally each sub-heading should contain 2-3 single sentence, concise bullet points containing the most salient points from your study. In the final bullet point of 'What Do These Findings Mean?', please include the main limitations of the study in non-technical language. Please see our author guidelines for more information: https://journals.plos.org/plosmedicine/s/revising-your-manuscript#loc-author-summary.

* Please express the main results with 95% CIs as well as p values. When reporting p values please report as p<0.001 and where higher as the exact p value p=0.002, for example. Throughout, suggest reporting statistical information as follows to improve clarity for the reader "22% (95% CI [13%,28%]; p</=)". Please be sure to define all numerical values at first use.

* When reporting ranges, please use a comma instead of a hyphen to separate values.

* Please include page numbers and line numbers in the manuscript file. Use continuous line numbers (do not restart the numbering on each page).

* Please cite the reference numbers in square brackets. Citations should precede punctuation.

FIGURES AND TABLES

* Please provide titles and legends for all figures and tables (including those in Supporting Information files).

* Please define all abbreviations used in each figure/table (including those in Supporting Information files).

* Please consider avoiding the use of red and green in order to make your figure more accessible to those with color blindness.

SUPPLEMENTARY MATERIAL

* Please note that supplementary material will be posted as supplied by the authors. Therefore, please amend it according to the relevant comments outlined here.

* Please cite your Supporting Information as outlined here: https://journals.plos.org/plosmedicine/s/supporting-information

REFERENCES

* PLOS uses the numbered citation (citation-sequence) method and first six authors, et al.

* Please ensure that journal name abbreviations match those found in the National Center for Biotechnology Information (NCBI) databases (http://www.ncbi.nlm.nih.gov/nlmcatalog/journals), and are appropriately formatted and capitalised.

* Where website addresses are cited, please include the complete URL and specify the date of access (e.g. [accessed: 12/06/2023]).

* Please also see https://journals.plos.org/plosmedicine/s/submission-guidelines#loc-references for further details on reference formatting.

STUDY TYPE-SPECIFIC REQUESTS

MODELLING STUDIES

The following list is derived from Geoffrey P Garnett, Simon Cousens, Timothy B Hallett, Richard Steketee, Neff Walker. Mathematical models in the evaluation of health programmes. (2011) Lancet DOI:10.1016/S0140-6736(10)61505-X:

* Please provide a diagram that shows the model structure, including how the natural history of the disease is represented, the process and determinants of disease acquisition, and how the putative intervention could affect the system.

* Please provide a complete list of model parameters, including clear and precise descriptions of the meaning of each parameter, together with the values or ranges for each, with justification or the primary source cited and important caveats about the use of these values noted.

* Please provide a clear statement about how the model was fitted to the data, including goodness-of-fit measure, the numerical algorithm used, which parameter varied, constraints imposed on parameter values, and starting conditions.

* For uncertainty analyses, please state the sources of uncertainties quantified and not quantified [can include parameter, data, and model structure].

* Please provide sensitivity analyses to identify which parameter values are most important in the model. Uncertainty estimates seek to derive a range of credible results on the basis of an exploration of the range of reasonable parameter values. The choice of method should be presented and justified.

* Please discuss the scientific rationale for the choice of model structure and identify points where this choice could influence conclusions drawn. Please also describe the strength of the scientific basis underlying the key model assumptions.

* For studies that develop a prediction model or evaluate its performance, please ensure that the study is reported according to the TRIPOD statement (https://www.equator-network.org/reporting-guidelines/tripod-statement) and include the completed checklist as Supporting Information. Please add the following statement, or similar, to the Methods: "This study is reported as per the Transparent Reporting of a Multivariable Prediction Model for Individual Prognosis Or Diagnosis (TRIPOD) statement (S1 Checklist)." For studies using machine learning, please use the TRIPOD-AI checklist. When completing the checklist, please use section and paragraph numbers, rather than page numbers.

HEALTH ECONOMICS / COST-EFFECTIVENESS STUDIES

* Please ensure that the study is reported according to the CHEERS guideline (available from: https://www.equator-network.org/reporting-guidelines/cheers) and include the completed checklist as Supporting Information. Please add the following statement, or similar, to the Methods: "This study is reported as per the Strengthening the Consolidated Health Economic Evaluation Reporting Standards 2022 (CHEERS 2022) Statement (S1 Checklist)." When completing the checklist, please use section and paragraph numbers, rather than page numbers.

# Author comment: Author response to Decision Letter 2

**DOI:** 10.1371/journal.pmed.1004739.r003

12 Mar 2026

Attachment Submitted filename: ResponseToReviewersEditors.docx

# Peer review: Decision Letter 2

**DOI:** 10.1371/journal.pmed.1004739.r004

**Authors:** Alison Farrell

7 Apr 2026

Dear Dr. Liu,

Thank you very much for re-submitting your manuscript "Comparative impacts and cost-effectiveness of tuberculosis systematic screening strategies in prisons in Brazil, Colombia, and Peru: a mathematical modeling study" (PMEDICINE-D-25-02987R2) for review by PLOS Medicine.

I have discussed the paper with my colleagues and the academic editor and it was also seen again by four reviewers. I am pleased to say that provided the remaining editorial and production issues are dealt with we are planning to accept the paper for publication in the journal.

We ask you to respond to the remaining comments of reviewer #2 and to the comments of the statistical reviewer that we engaged in this round of review (reviewer #4).

The remaining issues that need to be addressed are listed at the end of this email. Any accompanying reviewer attachments can be seen via the link below. Please take these into account before resubmitting your manuscript:

[LINK]

***Please note while forming your response, if your article is accepted, you may have the opportunity to make the peer review history publicly available. The record will include editor decision letters (with reviews) and your responses to reviewer comments. If eligible, we will contact you to opt in or out.***

In revising the manuscript for further consideration here, please ensure you address the specific points made by each reviewer and the editors. In your rebuttal letter you should indicate your response to the reviewers' and editors' comments and the changes you have made in the manuscript. Please submit a clean version of the paper as the main article file. A version with changes marked must also be uploaded as a marked up manuscript file.

Please also check the guidelines for revised papers at http://journals.plos.org/plosmedicine/s/revising-your-manuscript for any that apply to your paper. If you haven't already, we ask that you provide a short, non-technical Author Summary of your research to make findings accessible to a wide audience that includes both scientists and non-scientists. The Author Summary should immediately follow the Abstract in your revised manuscript. This text is subject to editorial change and should be distinct from the scientific abstract.

In addition to these revisions, you may need to complete some formatting changes, which you will receive in a follow up email. A member of our team will be in touch with a set of requests shortly. If you do not receive a separate email within a few days, please assume that checks have been completed, and no additional changes are required.

We expect to receive your revised manuscript within 1 week. Please email us (plosmedicine@plos.org) if you have any questions or concerns.

We ask every co-author listed on the manuscript to fill in a contributing author statement. If any of the co-authors have not filled in the statement, we will remind them to do so when the paper is revised. If all statements are not completed in a timely fashion this could hold up the re-review process. Should there be a problem getting one of your co-authors to fill in a statement we will be in contact. YOU MUST NOT ADD OR REMOVE AUTHORS UNLESS YOU HAVE ALERTED THE EDITOR HANDLING THE MANUSCRIPT TO THE CHANGE AND THEY SPECIFICALLY HAVE AGREED TO IT.

Please ensure that the paper adheres to the PLOS Data Availability Policy (see http://journals.plos.org/plosmedicine/s/data-availability), which requires that all data underlying the study's findings be provided in a repository or as Supporting Information. For data residing with a third party, authors are required to provide instructions with contact information for obtaining the data. PLOS journals do not allow statements supported by "data not shown" or "unpublished results." For such statements, authors must provide supporting data or cite public sources that include it.

To enhance the reproducibility of your results, we recommend that you deposit your laboratory protocols in protocols.io, where a protocol can be assigned its own identifier (DOI) such that it can be cited independently in the future. Additionally, PLOS ONE offers an option to publish peer-reviewed clinical study protocols. Read more information on sharing protocols at https://plos.org/protocols?utm_medium=editorial-email&utm_source=authorletters&utm_campaign=protocols

Please review your reference list to ensure that it is complete and correct. If you have cited papers that have been retracted, please include the rationale for doing so in the manuscript text, or remove these references and replace them with relevant current references. Any changes to the reference list should be mentioned in the rebuttal letter that accompanies your revised manuscript.

Please note, when your manuscript is accepted, an uncorrected proof of your manuscript will be published online ahead of the final version, unless you've already opted out via the online submission form. If, for any reason, you do not want an earlier version of your manuscript published online or are unsure if you have already indicated as such, please let the journal staff know immediately at plosmedicine@plos.org.

If you have any questions in the meantime, please contact me or the journal staff on plosmedicine@plos.org.

We look forward to receiving the revised manuscript by Apr 14 2026 11:59PM.

Sincerely,

Alison Farrell, Ph.D.

Senior Editor

PLOS Medicine

plosmedicine.org

------------------------------------------------------------

Requests from Editors:

Abstract and Author Summary

* Please ensure that the Introduction ends with a clear description of the study question or hypothesis.

* Please ensure that all abbreviations are defined at first use throughout the text.

* Please confirm that all numbers presented in the abstract are present and identical to numbers presented in the main manuscript text.

* In the last sentence of the Abstract Methods and Findings section, please describe the main limitation(s) of the study's methodology.

* In the author summary, in the final bullet point of 'What Do These Findings Mean?', please include the main limitations of the study in non-technical language.

Line 94: suggest to replace 'algorithm' with 'screening strategy'.

Figures and Tables

There is a formatting error in Table 3, upper right

Please add footnotes to Tables defining all abbreviations used.

If it is not feasible to make the y-axes the same among panels within the same figure (e.g. Fig. 3A, 3B (x and y axes in B), and the upper and lower panels of Fig.2), please acknowledge the discrepancies in the figure legends.

* Please define all elements of box plots in the figure caption - center line, box limits and whiskers.

* Where data points are discrete, please ensure that they are depicted in the figures as discrete data and not as a continuous line.

* Please provide the unadjusted comparisons as well as the adjusted comparisons in all relevant Tables.

Methods

Please add a Statistical Analyses subsection to the Methods.

* Please follow this guidance for Statistical reporting:

Please report statistical information as follows to improve clarity for the reader "22% (95% CI [13,28]; p</=)".

- Please separate upper and lower bounds with commas instead of hyphens as the latter can be confused with reporting of negative values.

- Please repeat statistical definitions (HR, CI etc.) for each set of parentheses.

Please include a clean version of the supporting information.

Please move the Disclaimer out of the funding statement and add a Disclaimer subsection.

Please create a DOI for your data and code and add to the Data Availability Statement.

Competing interests:

* Please add this statement to the manuscript's Competing Interests: "JG-F is an Academic Editor on PLOS Medicine's editorial board."

* All authors must declare their relevant competing interests per the PLOS policy, which can be seen here: https://journals.plos.org/plosmedicine/s/competing-interests For authors with ties to industry, please indicate whether any of the interests has a financial stake in the results of the current study.

Authors MCCA, BM, MAG, JTT, CAMR, AFMR, CB, PAJ, DMP, FDCJ have not completed their COI questionnaire.

Other:

* Please review your manuscript and edit to ensure compliance with our inclusive language requirements https://journals.plos.org/plosmedicine/s/human-subjects-research#loc-categorization

* Please include an Acknowledgments section in your manuscript.

Please ensure that statements in the manuscript that also appear in the manuscript metadata are identical.

For modeling papers, we ask that you follow the guidance from Geoffrey P Garnett, Simon Cousens, Timothy B Hallett, Richard Steketee, Neff Walker. Mathematical models in the evaluation of health programmes. (2011) Lancet DOI:10.1016/S0140-6736(10)61505-X:

* Please provide a diagram that shows the model structure, including how the disease natural history is represented, the process and determinants of disease acquisition, and how the putative intervention could affect the system.

* Please provide a complete list of model parameters, including clear and precise descriptions of the meaning of each parameter, together with the values or ranges for each, with justification or the primary source cited, and important caveats about the use of these values noted.

* Please provide a clear statement about how the model was fitted to the data including where relevant goodness-of-fit measure, the numerical algorithm used, which parameter varied, constraints imposed on parameter values, and starting conditions.

* For uncertainty analyses, please state the sources of uncertainties quantified and not quantified this can include parameter, data, and model structure.

* Please provide sensitivity analyses to identify which parameter values are most important in the model. Uncertainty estimates seek to derive a range of credible results on the basis of an exploration of the range of reasonable parameter values. The choice of method should be presented and justified.

* Please discuss the scientific rationale for this choice of model structure and identify points where this choice could influence conclusions drawn. Please also describe the strength of the scientific basis underlying the key model assumptions.

Comments from Reviewers:

Reviewer #2: Thank you for providing this excellent update of your manuscript. I think you have addressed my and the other reviewer comments thoroughly and thoughtfully.

I have 2 trivial comments related to figures in the appendix.

Figure S7: Suggest you indicate in the legend that the straight lines in each panel reflect the WTP threshold.

Figure S8: The legend mentions open squares, but I didn't see these in the figure.

Reviewer #3: I want to thank the authors for the thoughtful and thorough response to our comments. It is an excellent paper. Good to go for me.

Reviewer #4: Here Liu et al. extend a previously developed mathematical model of TB incidence in prison populations in Brazil, Colombia, and Peru. I was asked to review this manuscript as a statistical reviewer during revision.

My overall impression is that the analysis and modeling are thorough, handling of key limitations is pragmatic and addressed in the discussion. The results are interesting and could be of important use for implementation of such interventions. Implicit in this work is how diagnostic specificity/sensitivity for TB detection impacts the cost and epidemiology of managing TB.

My comments are fairly minor.

My main comment with the model is regarding the assumption that contact rates are the same between recently incarcerated and never incarcerated. This is a strong assumption and it is plausible that recently incarcerated populations are particularly sensitive and could be at a higher risk. This is very briefly discussed in [4], but the implications of this assumption could be compounded here since the impact of screening lags into this cohort. This should at least be mentioned in the discussion.

How are the interventions implemented into the structural model simulation? For example, is the bi-annual screening treated as a continuous process (constant rate) or discrete (pulse process, stiff system)?

- I would find it helpful if the appendix explained the technical differences between interventions; for example, by comparing the annual vs. biannual implementations.

Explicitly state your definition of a false positive. I assumed a false positive is any non-infected individual (specifically S) who screens positive.

- Related, why would test-positive individuals in the early latent (E) state who are treated be classified as a false positive? It seems like an unintended benefit to catch TB in these populations with mass screening

- Discussion point: There's an implicit assumption that false positives go through a full confirmatory work-up, but it's possible that could be stopped early (eg, after a culture negative test). The approach may overestimate costs in this algorithm.

I inferred that the Xpert pool algorithm is hampered by the loss of sensitivity due to pooling 8 samples. Is that a reasonable conclusion? Could the authors comment on using smaller pool sizes. Perhaps it's not cost effective but might be worth stating that.

Figure 1, the color arrow intervention implies only I -> R, but I assume any treated infected (E, L) individual moves to R?

Figure 2, what do the boxplot distributions represent (e.g., each model simulation, with what N), and were outliers present?

The calibration was somewhat unclear to me: The methods read as if the models were re-calibrated for this manuscript using the methodology in [4], while the appendix states the calibrated parameters were sourced directly from [4]. Please clarify which parameters were re-used and which were re-calibrated, this could be simply annotated with minor editing to Table S1.

I found the following sentence too dense: "We then conducted multinomial logistic regression and estimated the probability that each strategy would be optimal across the range of prison incidence levels." I suggest expanding on this in the Appendix so that it is reproducible. What is the input data (n and what each observation unit represents), the dependent multinomial variable, the predictors, key quantitative outcomes extracted from the model, and how it was fit (software)?

Any attachments provided with reviews can be seen via the following link:

[LINK]

# Author comment: Author response to Decision Letter 3

**DOI:** 10.1371/journal.pmed.1004739.r005

17 Apr 2026

Attachment Submitted filename: ResponseToReviewersEditors_Round2.docx

# Editor report: Decision Letter 3

**DOI:** 10.1371/journal.pmed.1004739.r006

**Authors:** Alison Farrell

21 Apr 2026

Dear Dr Liu,

On behalf of my colleagues and the Academic Editor, Brooke Nichols, I am pleased to inform you that we have agreed to publish your manuscript "Comparative impacts and cost-effectiveness of tuberculosis systematic screening strategies in prisons in Brazil, Colombia, and Peru: a mathematical modeling study" (PMEDICINE-D-25-02987R3) in PLOS Medicine.

Before your manuscript can be formally accepted you will need to complete some formatting changes, which you will receive in a follow up email. Please be aware that it may take several days for you to receive this email; during this time no action is required by you. Once you have received these formatting requests, please note that your manuscript will not be scheduled for publication until you have made the required changes.

Please note that the hollow arrows in gold and purple are difficult to discern in Figure 1. If possible, I suggest using a wider space between the two lines.

In the meantime, please log into Editorial Manager at http://www.editorialmanager.com/pmedicine/, click the "Update My Information" link at the top of the page, and update your user information to ensure an efficient production process.

PRESS

We frequently collaborate with press offices. If your institution or institutions have a press office, please notify them about your upcoming paper at this point, to enable them to help maximise its impact. If the press office is planning to promote your findings, we would be grateful if they could coordinate with medicinepress@plos.org. If you have not yet opted out of the early version process, we ask that you notify us immediately of any press plans so that we may do so on your behalf.

We also ask that you take this opportunity to read our Embargo Policy regarding the discussion, promotion and media coverage of work that is yet to be published by PLOS. As your manuscript is not yet published, it is bound by the conditions of our Embargo Policy. Please be aware that this policy is in place both to ensure that any press coverage of your article is fully substantiated and to provide a direct link between such coverage and the published work. For full details of our Embargo Policy, please visit http://www.plos.org/about/media-inquiries/embargo-policy/.

To enhance the reproducibility of your results, we recommend that you deposit your laboratory protocols in protocols.io, where a protocol can be assigned its own identifier (DOI) such that it can be cited independently in the future. Additionally, PLOS ONE offers an option to publish peer-reviewed clinical study protocols. Read more information on sharing protocols at https://plos.org/protocols?utm_medium=editorial-email&utm_source=authorletters&utm_campaign=protocols

Thank you again for submitting to PLOS Medicine. We look forward to publishing your paper.

Sincerely,

Alison Farrell, Ph.D.

Senior Editor

PLOS Medicine
